# Enhancing winter wheat prediction with genomics, phenomics and environmental data

**DOI:** 10.1186/s12864-024-10438-4

**Published:** 2024-05-31

**Authors:** Osval A. Montesinos-López, Andrew W. Herr, José Crossa, Abelardo Montesinos-López, Arron H. Carter

**Affiliations:** 1 Facultad de Telemática, Universidad de Colima, Colima, 28040 México; 2 Department of Crop and Soil Sciences, Washington State University, Pullman, WA 99164 USA; 3 International Maize and Wheat Improvement Center (CIMMYT), Km 45, Carretera México- Veracruz, Edo. de México, CP 52640 México; 4 Universidad de Guadalajara, Montecillos, Edo. de México, CP 56230 México

**Keywords:** Genomic prediction, Integrating additional inputs, Phenomics, Environmental information, Genomics, Multi-environment trails

## Abstract

In the realm of multi-environment prediction, when the goal is to predict a complete environment using the others as a training set, the efficiency of genomic selection (GS) falls short of expectations. Genotype by environment interaction poses a challenge in achieving high prediction accuracies. Consequently, current efforts are focused on enhancing efficiency by integrating various types of inputs, such as phenomics data, environmental information, and other omics data. In this study, we sought to evaluate the impact of incorporating environmental information into the modeling process, in addition to genomic and phenomics information. Our evaluation encompassed five data sets of soft white winter wheat, and the results revealed a significant improvement in prediction accuracy, as measured by the normalized root mean square error (NRMSE), through the integration of environmental information. Notably, there was an average gain in prediction accuracy of 49.19% in terms of NRMSE across the data sets. Moreover, the observed prediction accuracy ranged from 5.68% (data set 3) to 60.36% (data set 4), underscoring the substantial effect of integrating environmental information. By including genomic, phenomic, and environmental data in prediction models, plant breeding programs can improve selection efficiency across locations.

**Supplementary Information:**

The online version contains supplementary material available at 10.1186/s12864-024-10438-4.

## Background

Increasing agricultural productivity is crucial for food security, efficient resource use, economic development, and climate change adaptation to meet the needs of a growing global population. The main challenges to increasing agricultural productivity include limited resources, climate change, pests and diseases, soil degradation, technological gaps, smallholder farming, sustainability concerns, and access to knowledge and extension services [1]. Addressing these challenges requires sustainable resource use, climate adaptation strategies, effective pest and disease management, soil conservation practices, bridging technological gaps, supporting smallholder farmers, balancing productivity with environmental sustainability, and strengthening knowledge dissemination and extension services [1].

Plant breeding techniques like genomic selection (GS) are crucial for increasing food production as they expedite plant breeding efforts by enhancing trait selection in crops [2]. By utilizing genetic markers, GS enables breeders to identify high-yielding and disease-resistant varieties more efficiently. This technique accelerates the development of superior crops, saving time and resources compared to traditional plant breeding methods [3]. GS’s precision and efficiency in trait selection contribute to the development of crops adapted to environmental changes, including climate resilience. Ultimately, it optimizes the genetic potential of crops, enhances productivity, and plays a vital role in global food security by producing improved varieties with higher yield, quality, and resilience [3].

Implementing GS in plant breeding programs faces challenges such as lack of high-quality genomic data, computational expertise requirements, and the need for high quality phenotypic data [2, 4]. Multi-environment trials (MET) play a crucial role in the context of plant breeding programs. MET involves testing plant varieties across multiple environments to assess their performance under diverse conditions, allowing researchers to identify genotypes that exhibit consistent superiority across varying settings [5, 6]. In MET, GS can enhance the efficiency of variety selection by providing accurate predictions of genotype performance across different environments. However, the application of GS in MET is not without challenges. One limitation lies in the complex genotype-by-environment interactions, where the performance of genotypes varies across different environmental conditions. GS models may struggle to accurately capture and predict these interactions. Previous attempts to apply genomic selection in MET have encountered difficulties in achieving robust predictions due to the intricate nature of genotype-environment interplay [7–9]. Additionally, limitations in the availability and representativeness of training data, as well as the need for sophisticated statistical methodologies, pose further challenges to the successful implementation of GS in MET. Addressing these issues is essential for realizing the full potential of GS in improving crop performance across diverse environments. For this reason, transferring prediction models across different environments and genetic backgrounds is complex due to strong genotype-by-environment interactions, which often produce low prediction accuracies [2]. It is also essential to foster a close collaboration among breeders, geneticists, statisticians and bioinformaticians to implement GS successfully.

As mentioned previously, high prediction accuracies are crucial for the successful implementation of the GS methodology for several reasons [10]. First, accurate predictions enable breeders to identify and select individuals with the highest genetic potential for desired traits, improving the efficiency and effectiveness of breeding programs [11]. This leads to faster genetic progress and the development of improved varieties with desired traits, such as higher yields or disease resistance. Additionally, high prediction accuracies reduce the costs and time associated with phenotypic evaluations by allowing breeders to prioritize individuals for further testing based on their predicted performance. Accurate predictions also minimize the risk of selecting individuals with false positive or false negative results, ensuring that resources are allocated to individuals with the highest breeding value. Ultimately, high prediction accuracies contribute to the overall success and impact of GS in driving genetic improvement in crops and increasing agricultural productivity [12].

For this reason, there is a lot of empirical evidence suggesting that to increase the prediction accuracy of the GS methodology, it is important to integrate more than one type of input, like genomic information, phenomics data, and environmental information [7, 13–22]. First, genomic information provides insights into the underlying genetic variations that influence complex traits in plants, enabling breeders to make informed selections based on desired genetic profiles [11]. Second, phenomics data, obtained through unmanned aerial systems (UAS) or other advanced technologies, captures detailed information about plant traits and their responses to environmental conditions, allowing for a more comprehensive assessment of plant performance [23, 24]. By integrating these two types of data, breeders can better understand the genotype by environment interactions and identify individuals with superior performance across diverse environments. Additionally, incorporating environmental information, such as climate data, soil characteristics, or field management practices, helps account for the environmental variability that affects trait expression [10, 18, 21]. This integration enables breeders to develop predictive models that consider the complex interactions between genotypes, phenotypes, and environments, resulting in more accurate predictions of plant performance and selection of individuals with higher breeding values. Moreover, the integration of these data sources facilitates the identification of genetic markers associated with specific traits of interest, allowing for more precise GS and targeted breeding efforts. It also enables the development of predictive models that can adapt to changing environmental conditions, thereby enhancing the resilience and adaptability of cultivated crops. Overall, the integration of genomic information, phenomics data (obtained through UAS or other advanced techniques) and environmental information has the potential to provide a comprehensive and multi-dimensional approach to genomic selection, leading to improved accuracy, efficiency, and effectiveness in crop improvement programs [7, 13–22].

The integration of environmental information, also referred to as enviromic data, into genomic prediction models has produced diverse outcomes [10, 13, 16, 18, 25–26]. Certain studies, such as those conducted by [10, 25], and [26] have demonstrated notable enhancements by incorporating this information. However, other investigations, including those by [18] and [13] have reported modest or negligible improvements. These mixed findings underscore the lack of a robust and precise method for the effective integration of environmental information into genomic prediction models. To address this gap [10], proposed the use of feature selection to identify optimal environmental predictors. Also, continuously growing is the use of UAS data as inputs to improve the prediction performance of traits of interest in GS [14, 15, 17, 20, 27, 28]. However, the incorporation of UAS data for genomic prediction is challenging. For example, the spatial resolution may not always meet fine-scale needs, and data quality can be affected by weather or technical issues. Temporal constraints and the need for substantial computational resources pose challenges. Standardization and compatibility issues, regulatory restrictions, and data privacy concerns add complexity. Additionally, UAS data sensitivity to environmental conditions may impact reliability. Addressing these limitations is crucial for optimizing the use of UAS data in accurate genomic predictions in agriculture. More recently there already exist some studies incorporating genomic data, environmental information, and phenomics data for GS [19, 28, 29]. These publications show some empirical evidence that integrating genomic data, environmental information, and phenomics data enhances genomic prediction by providing a holistic view of the genotype-environment-phenotype relationship. This approach improves predictive accuracy by capturing real-time phenotypic traits and dynamic interactions between genes and the environment. The synergy of these data sets holds great potential for more precise and effective genomic predictions in various fields, including agriculture, medicine, and conservation. However, the goal of our study is to increase empirical evidence that the method of feature selection for incorporating environmental information with genomic data, proposed by [10], helps improve prediction performance.

With the aim of further substantiating the benefits of integrating diverse inputs to enhance the prediction accuracy of the GS methodology, this study focused on looking for a more optimal integration of environmental data with genomics and phenomics information for the prediction of grain yield (GY), plant height (PH) and heading date (HD) traits in soft white winter wheat. However, now since our goal is to evaluate different approaches for integrating environmental information under a more optimal fashion, we did not also evaluate if there are significant improvements regarding including or not including UAS information, as was done in our previous publication [15]. For these reasons, in this research 14 different ways for integrating environmental information with genomic and phenomic information was evaluated with real data obtained from Washington State University, spanning 2019 to 2022. To assess the predictive performance, a cross-validation scheme involving partially tested lines in untested environments was implemented, specifically employing the leave-one-environment-out (LOEO) approach. Through this comprehensive approach, the study seeks to highlight the value of integrating multiple data sources to improve the accuracy of GS predictions.

## Results

The results are given for data sets 2, 3, 4 and 5. Data set 5 contains the information of data sets 1–4. Results for data set 1 are not given since it only contains two environments. The results are presented only in terms of Normalized Root Mean Squared Error (NRMSE). This metric is first used for one-to-one comparison of all models to subsequently determine the count of times a model outperforms the others. This process is carried out for both environments and traits. Then directly we compare the average of each model and calculate the percentage of improvement of all models relative to the model with the highest average NRMSE (worst model) using relative efficiency (RE). We also computed the relative efficiency of each model regarding model M0 denoted as RE_M0, since model M0 denotes the model without environmental covariates. It was noted that at times RE_M0 produced negative values since some models were worse than model M0.

### Data set 2 (2020)

Regarding the overall count per environment, model M7 outperformed all the others (144/225; this model wins in 144 out of 225 possible combinations). The second-best model was M6 (140/225), followed by M2 as the third-best model (124/225). Conversely, M0 turned out to be the worst-performing model (63/225), followed by M9 (72/225) and M10 (73/225). When contrasting by traits, the maximum number of times a model could outperform the others was 45. This means that, for this data set, there are a total of 45 possible combinations. Therefore, models M6 and M7 emerged as the best models compared to all the others (40/45), with M2 as the second-best model (28/45). On the other hand, M0 proved to be the worst model without winning at least once (0/45). As the second and third worst models, we have M10 and M9 (7/45 and 11/45, respectively).

**Fig. 1:**
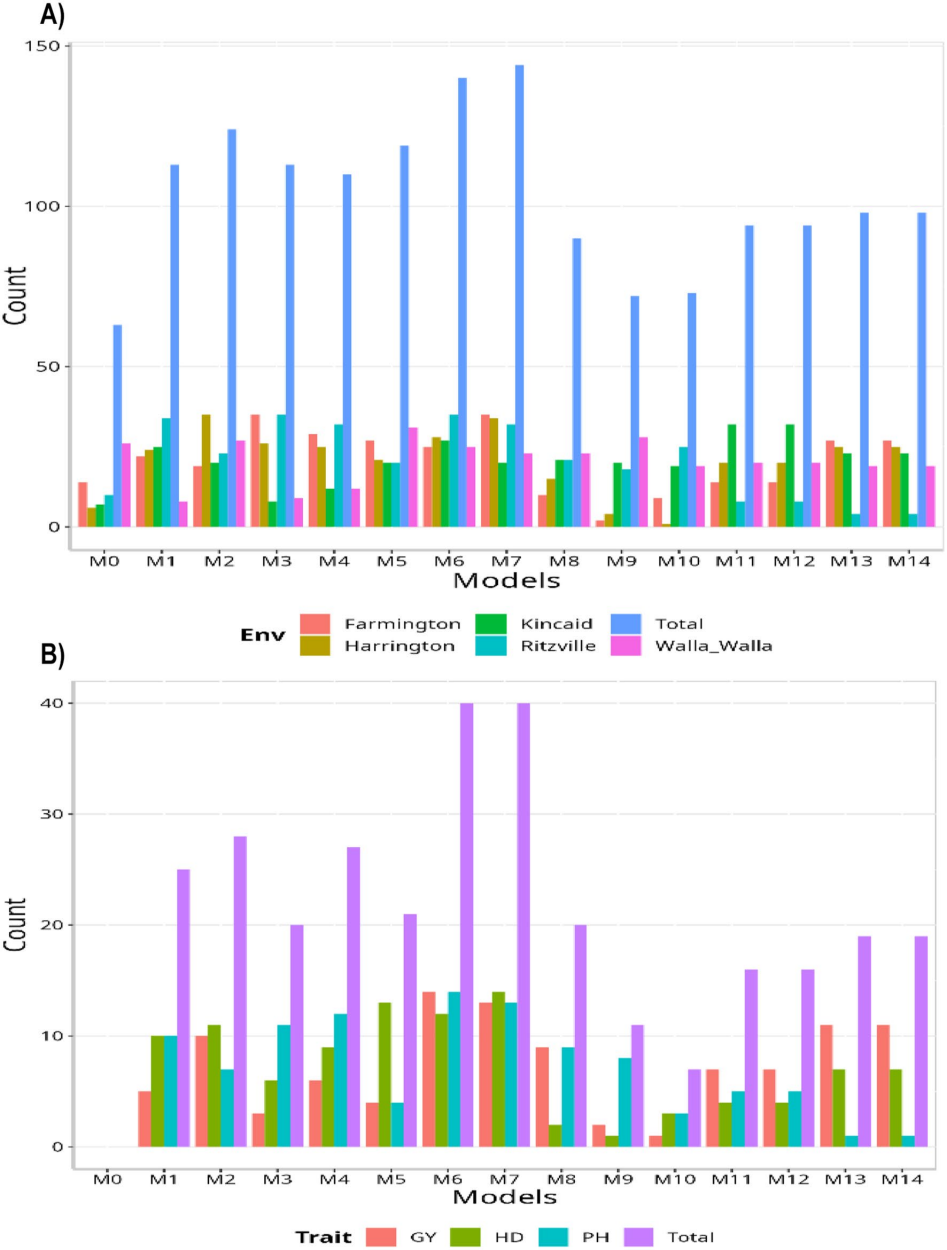
Data set 2 (2020). (**A**) Count of the number of times a model is better than another, by environment. (**B**) Count of the number of times a model is better than another, by trait

**Fig. 2:**
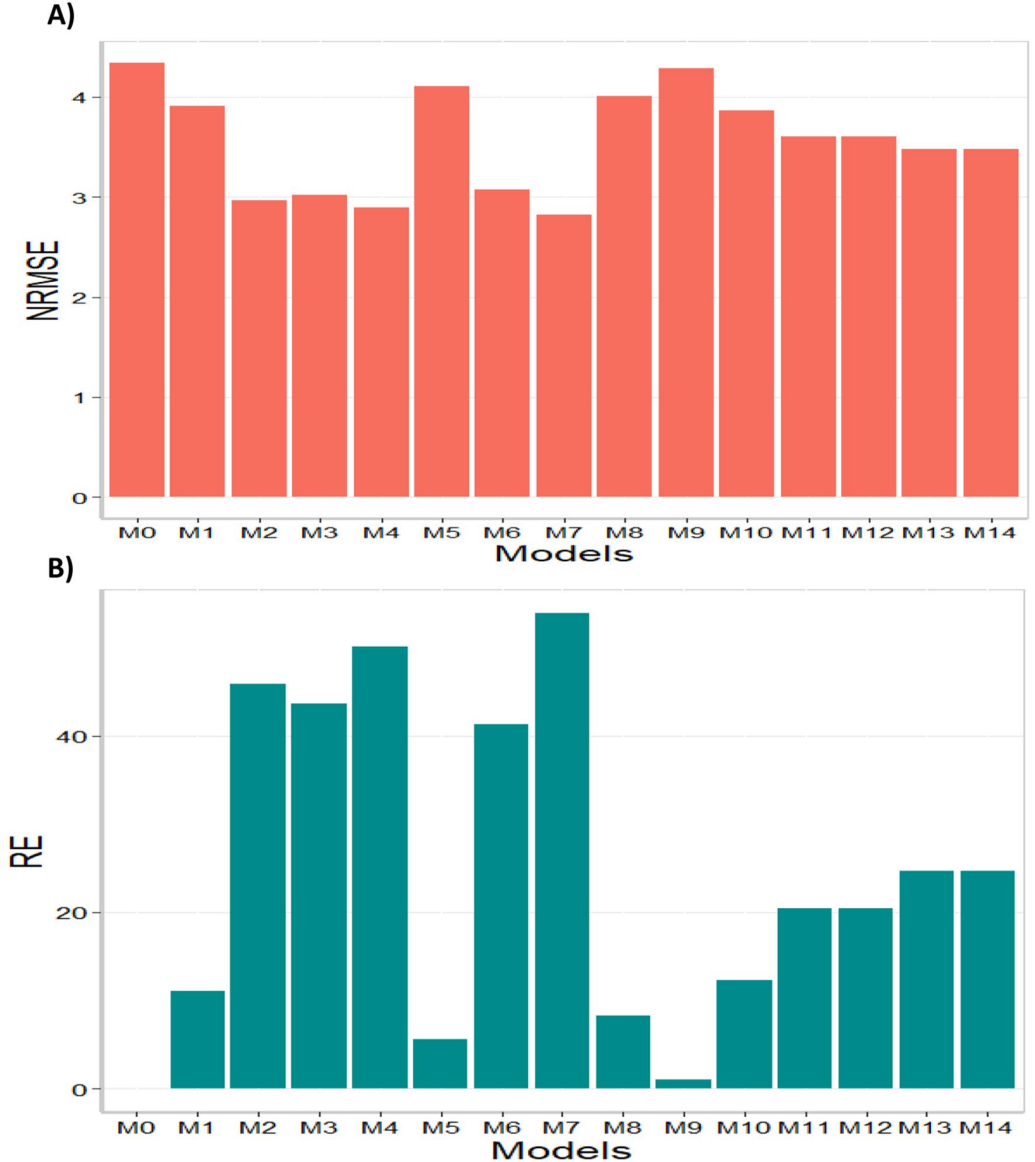
Data set 2 (2020). (**A**) Prediction accuracy of each predictor (M0 to M14) in terms of normalized root mean square error (NRMSE). (**B**) Relative efficiency (RE) of each model compared to the worst model (M0)

In terms of NRMSE model M7 was the best-performing model with the smallest NRMSE value (2.82). The second-best model was M4 (2.89), followed by M2 as the third-best model (NRMSE = 2.97). On the other hand, model M0 stood out as the worst-performing model (NRMSE = 4.34), with models M9 and M5 being the second and third worst models respectively (NRMSE = 4.29 and NRMSE = 4.11). In terms of RE, compared to the worst model (M0), the achieved gains were 53.89% for the best model, M7; 50.06% for M4; and 45.89% for M2. Ultimately, in terms of NRMSE, it is found that model M7 demonstrated the best predictive capability, while model M0 exhibited the worst predictive capacity. Finally, it is important to point out that model M0 is the model without environmental covariates. For this reason, in this data set it is observed that adding environmental covariates helps to significantly reduce the prediction error. The results of this data set are presented in Figs. 1 and 2 (refer to in Annex A Table A1 for detailed information).

### Data set 3 (2021)

Among the various models considered, model M6 stood out as the top-performing model in terms of total count per environment, surpassing all others (184/315; this model achieves victory in 184 out of 315 possible combinations). The second-best performing models were M11 and M12 (174/315), closely followed by M14 as the third-best model (161/315). Conversely, model M2 performed the poorest among all models (73/315), with models M7 (105/315) and M5 (112/315) ranking as the second and third worst models, respectively. When comparing the models based on specific traits, the maximum number of times a model could outperform the others is 45. Among the top-performing models, M14 claims the first position (33/45), followed closely by M6 (32/45), and M11 and M12 (29/45). On the other hand, models M2 and M7 performed the worst (2/45), with M4 (9/45) and M5 (10/45) occupied the second and third positions as the least effective models, respectively. Furthermore, model M0 fell in the middle of the ranking based on environments (147/315) and ranks as the fourth-best model when evaluated based on traits (28/45).

**Fig. 3:**
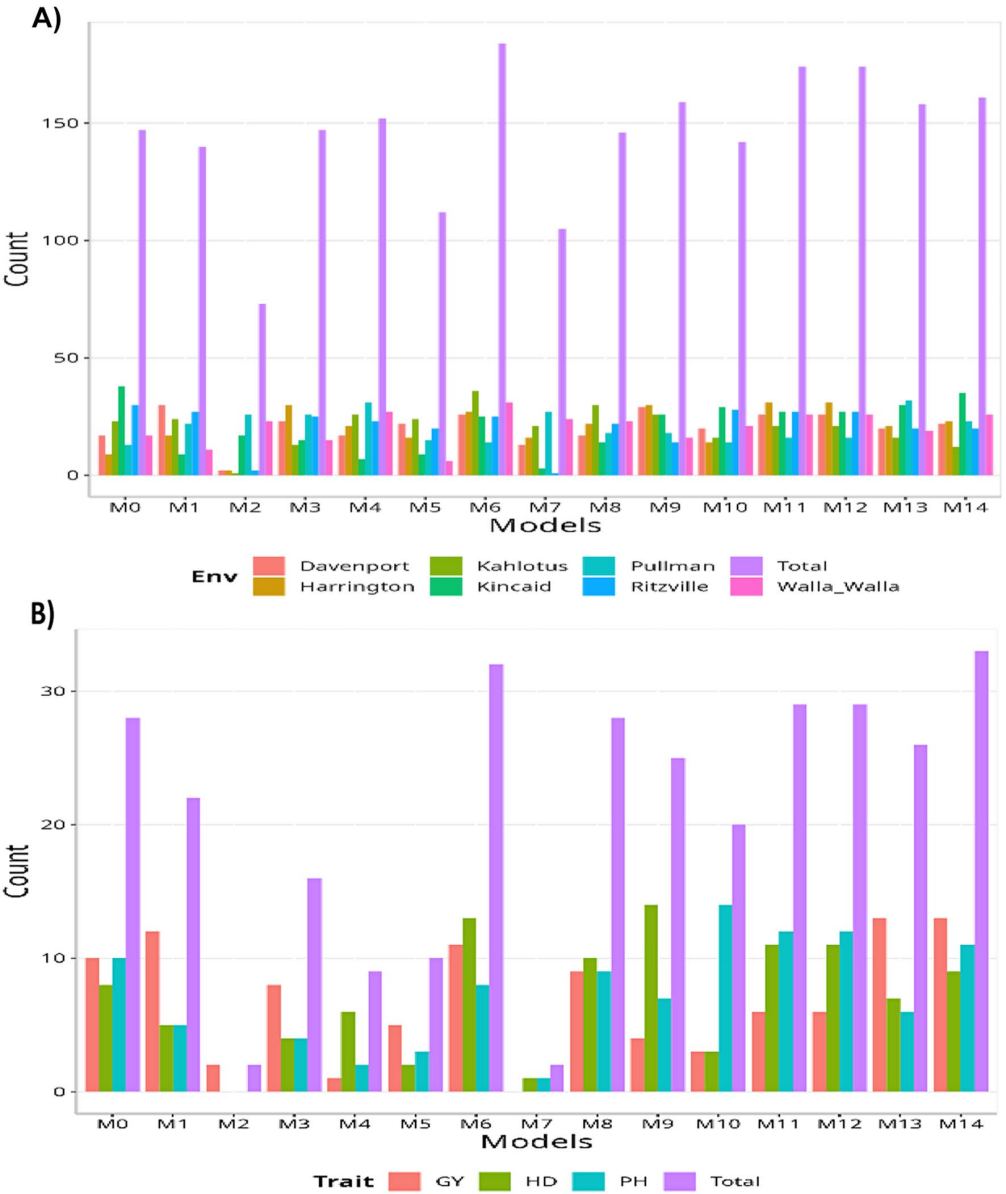
Data set 3 (2021). (**A**) Count of the number of times a model is better than another, by environment. (**B**) Count of the number of times a model is better than another, by trait

**Fig. 4:**
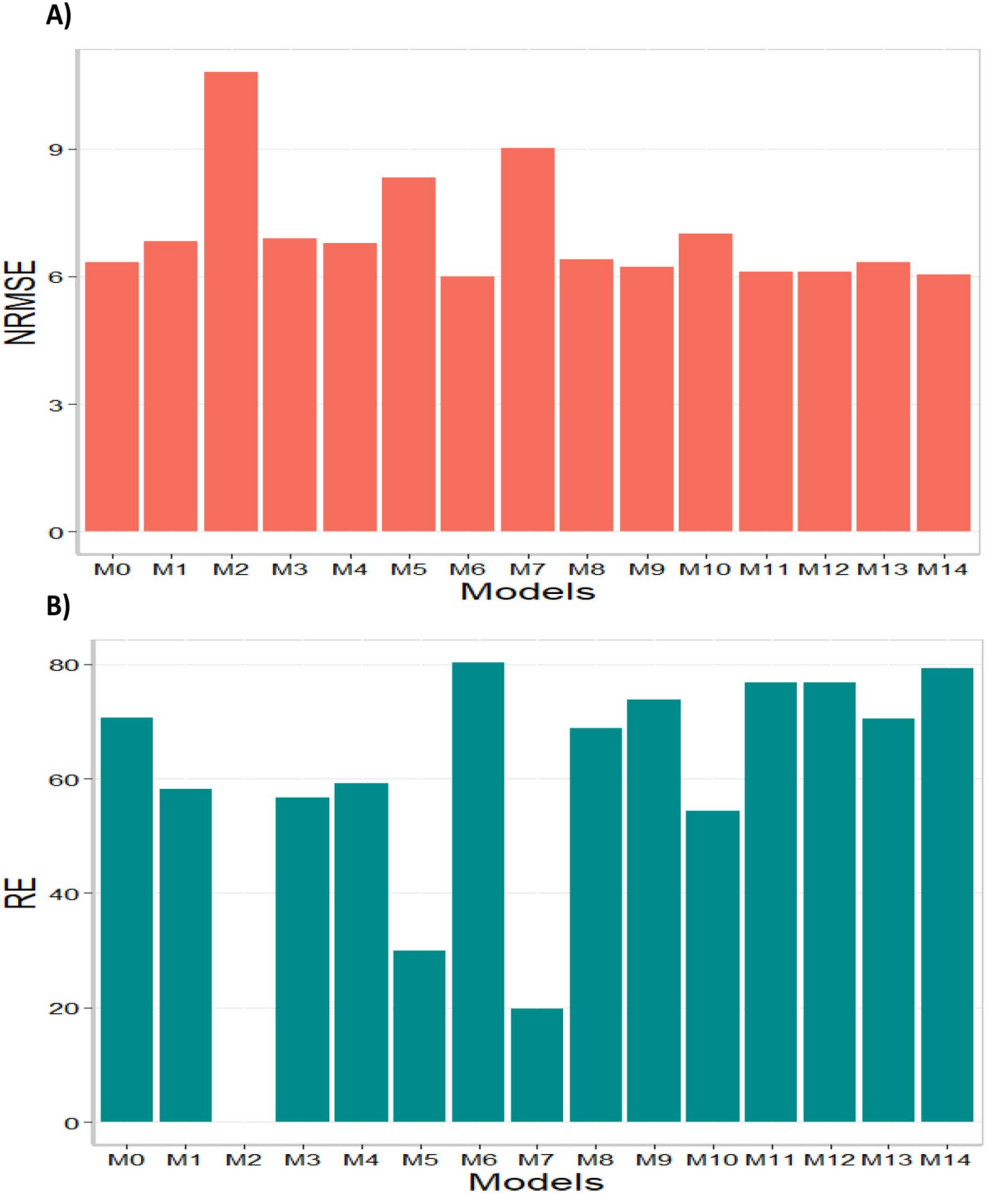
Data set 3 (2021). (**A**) Prediction accuracy of each predictor (M0 to M14) in terms of normalized root mean square error (NRMSE). (**B**) Relative efficiency (RE) of each model compared to the worst model (M2)

Based on the average NRMSE results, it is evident that model M6 outperformed other models with the smallest NRMSE value of 5.99. The second-best model was M14 with an NRMSE value of 6.03, followed by models M11 and M12 as the third-best models with NRMSE values of 6.11. On the contrary, model M2 performed the poorest with an NRMSE value of 10.80, while models M7 and M5 ranked as the second and third worst models, respectively, with NRMSE values of 9.02 and 8.31. In terms of RE, the gains achieved compared to the worst-performing model (M2) were substantial. Model M6 achieved a gain of 80.28%, followed by model M14 with a gain of 79.21%, and models M11 and M12 with gains of 76.79%. Furthermore, compared to model M0, the three best models showed gains of 5.68% (M0), 4.98% (M14), and 3.60% (M11 and M12), respectively (See last column of Table A2). Finally, regarding NRMSE, it can be concluded that model M6 demonstrated the best predictive capability, while model M2 exhibited the poorest predictive capacity. The results of this dataset are presented in Figs. 3 and 4 (for detailed information, refer to in Table A2). For details of the comparison of the 14 models to model M0 see column RE_M0 (%) of Table A2, representing the computed percentage of gain (or loss) of each model compared to model M0.

### Data set 4 (2022)

When considering the total count per environment, model M2 emerged as the best-performing model, surpassing all others (170/240; this model achieved victory in 170 out of 240 possible combinations). Following closely behind is model M6, positioned as the second-best model (167/240), while model M7 ranks as the third-best model (163/240). Conversely, model M0 ranks as the poorest-performing model (63/240), with models M13-M14 sharing the second worst position (64/240), and models M11-M12 ranking as the third worst models (69/240). Comparing the models based on traits, the maximum number of times a model could outperform the others is 45. Among the top-performing models, both models M2 and M6 share the first position (37/45), showcasing their strong performance. Following closely behind was model M7 (34/45), while model M3 claimed the third position (30/45). On the other hand, models M13 and M14 have an equal count, making them both the worst models in terms of traits (6/45), followed by model M0 (9/45) and M9 (11/45). Notably, model M0 appeared among the two worst models in both the environment and trait analyses, indicating its consistently poor performance across different evaluations. In Table A3, the column RE (%) are computed as the percentage of gain of each model regarding the worst model that in this data set (M0), while in the last column of Table A3 RE_M0 (%) are computed as the percentage of gain of each model regarding the model without using environmental covariates (M0). The results of both columns [RE (%) and RE_M0 (%)] of Table A3 are the same since in this data set M0 resulted in the worst model in terms of prediction error.

**Fig. 5:**
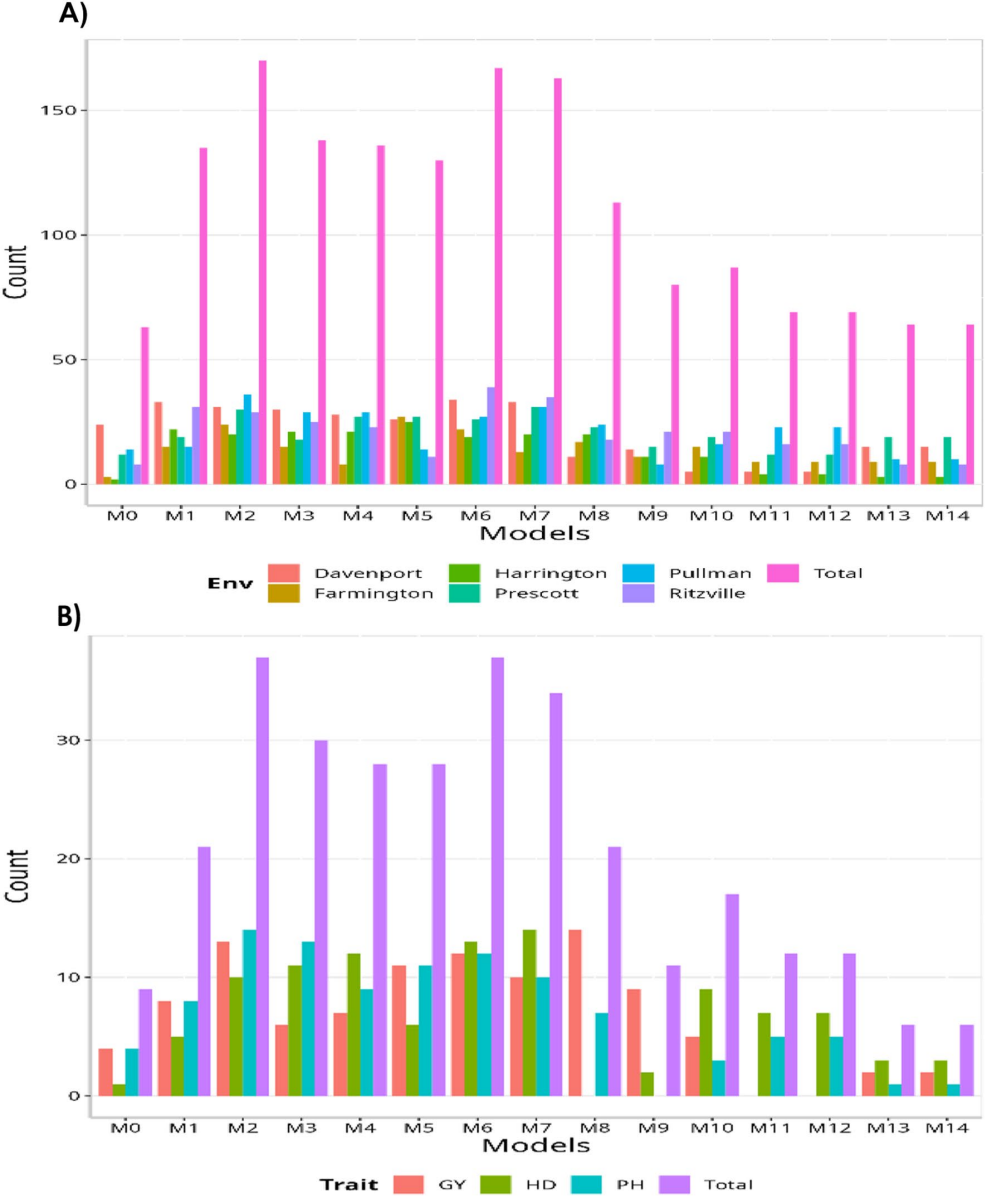
Data set 4 (2022). (**A**) Count of the number of times a model is better than another, by environment. (**B**) Count of the number of times a model is better than another, by trait

**Fig. 6:**
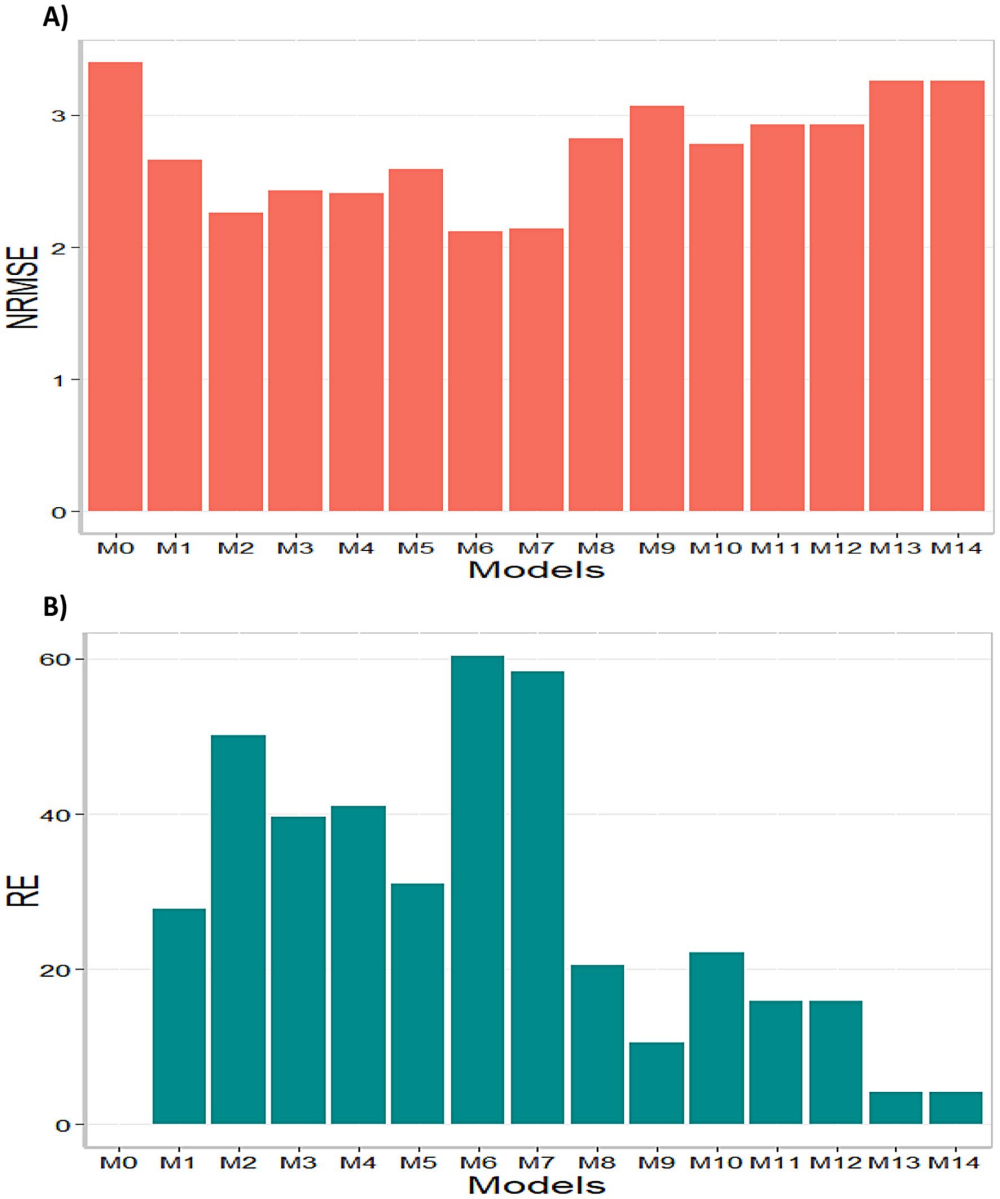
Data set 4 (2022). (**A**) Prediction accuracy of each predictor (M0 to M14) in terms of normalized root mean square error (NRMSE). (**B**) Relative efficiency (RE) of each model compared to the worst model (M0)

Based on the NRMSE, model M6 emerged as the best-performing model with the smallest NRMSE value of 2.12. Following closely behind was model M7 as the second-best model with an NRMSE value of 2.14, while model M2 ranked as the third-best model with an NRMSE value of 2.26. Conversely, model M0 stood out as the worst-performing model with an NRMSE value of 3.40, and models M13 and M14 occupied the second worst position with an NRMSE value of 3.26. In terms of relative error (RE), the gains achieved compared to the worst-performing model (M0) were significant. Model M6 achieved a gain of 60.36%, followed by model M7 with a gain of 58.40%, and model M2 with a gain of 50.08%. Finally, it can be concluded that for this particular data set, model M6 demonstrated the best predictive capability, while model M0 exhibited the poorest predictive capacity. The results of this data set are presented in Figs. 5 and 6 (for detailed information, refer to Table A3).

### Data set 5 (all years together)

When analyzing the total count per environment, it is evident that model M1 outperformed all other models, winning in 542 out of 870 possible combinations (542/870). Models M13 and M14 ranked as the second-best models, both achieving a count of 466 out of 870 (466/870). Following closely behind was model M8, positioned as the third-best model with a count of 362 out of 870 (362/870). On the other hand, model M2 performed the poorest with a count of 276 out of 870 (276/870), while model M4 ranked as the second-worst model with a count of 306 out of 870 (306/870). In terms of comparing the models based on traits, the maximum number of times a model could outperform the others is 45, representing all possible combinations within the data set. Model M1 stood out as the best-performing model, surpassing all others with a count of 40 out of 45 (40/45). Following closely behind was model M3 as the second-best model with a count of 26 out of 45 (26/45), while model M9 claimed the third-best position with a count of 25 out of 45 (25/45). Model M2 consistently ranked as the worst-performing model, with a count of only 3 out of 45 (3/45), aligning with its performance based on environments. Additionally, model M4 was positioned as the second-worst model with a count of 10 out of 45 (10/45).

**Fig. 7:**
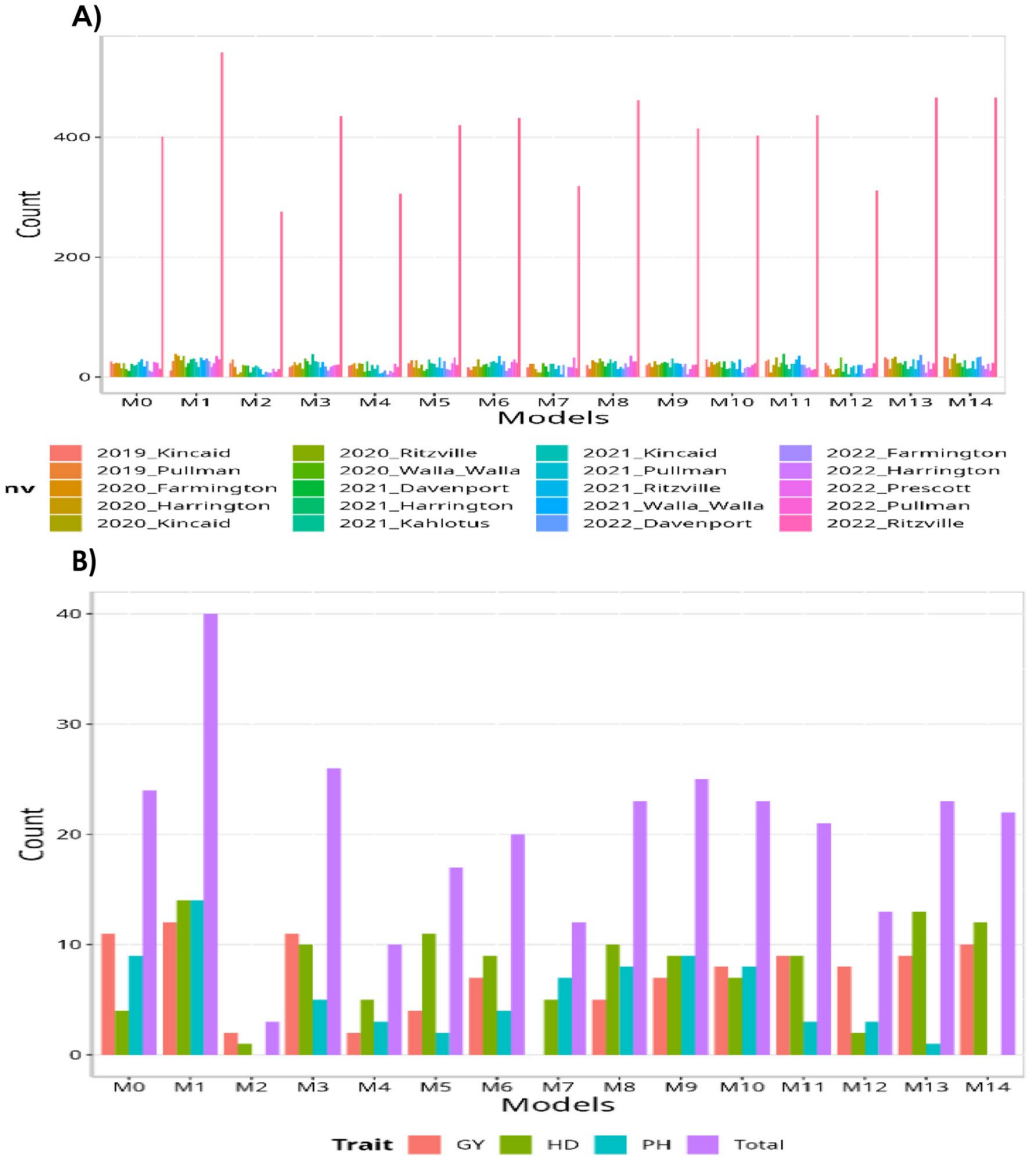
Data set 5 (all years together). (**A**) Count of the number of times a model is better than another, by environment. (**B**) Count of the number of times a model is better than another, by trait

**Fig. 8:**
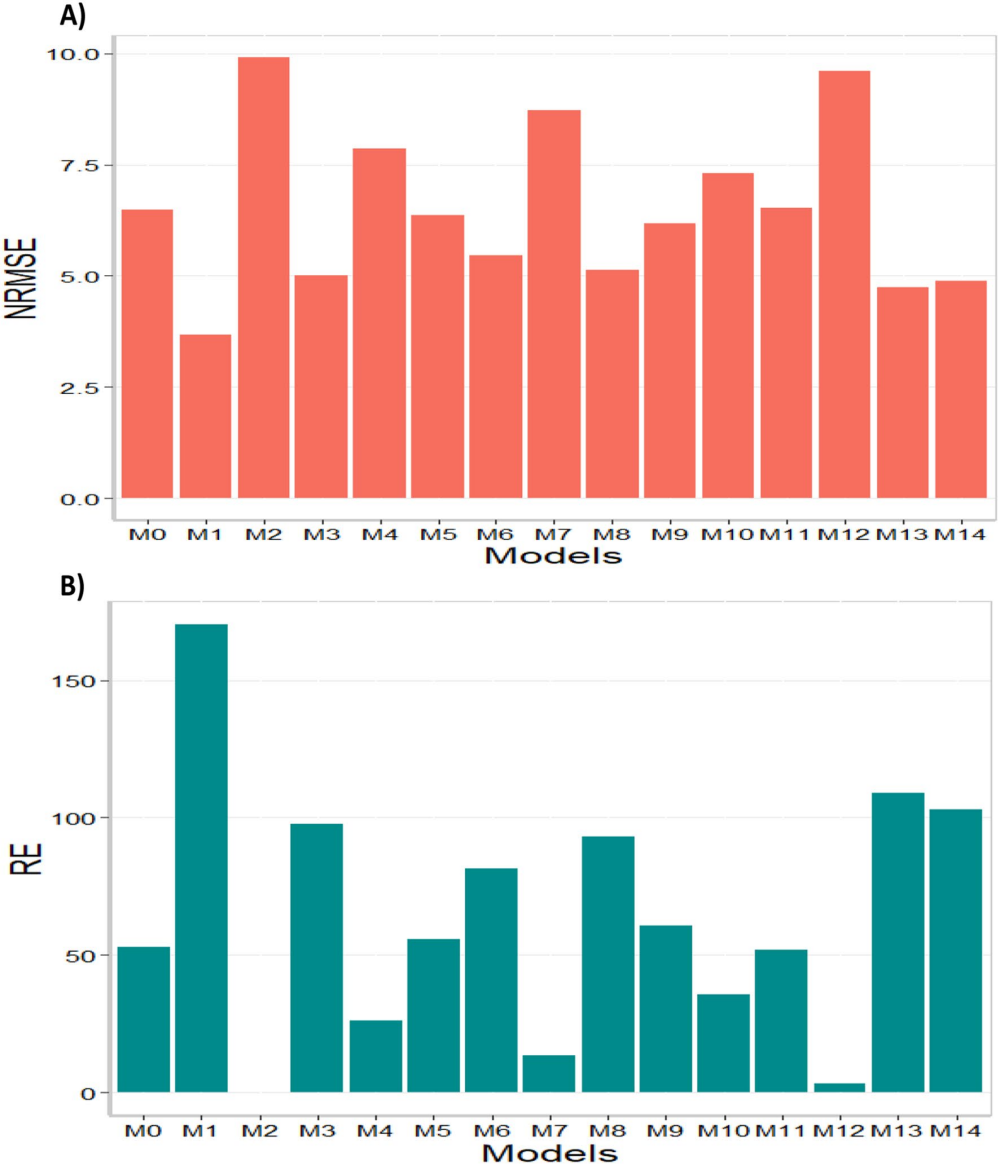
Data set 5 (all years together). (**A**) Prediction accuracy of each predictor (M0 to M14) in terms of normalized root mean square error (NRMSE). (**B**) Relative efficiency (RE) of each model compared to the worst model (M2)

Based on the NRMSE, model M1 stood out as the best-performing model, achieving the smallest NRMSE value of 3.67. Following closely behind was model M13 as the second-best model with an NRMSE value of 4.74, while model M14 ranked as the third-best model with an NRMSE value of 4.89. Conversely, model M2 demonstrated the poorest performance with an NRMSE value of 9.91, and models M12 and M7 ranked as the second and third worst models, respectively, with NRMSE values of 9.61 and 8.73. Considering RE, the gains achieved compared to the worst-performing model (M2) were substantial. Model M1 exhibited a gain of up to 170.26%, followed by model M13 with a gain of 109.03%, and model M14 with a gain of 102.82%. Additionally, the top three models showed gains of 76.84% (M1), 36.92% (M13), and 32.76% (M14), respectively, compared to model M0 (the model without using environmental covariates). From these results, it can be concluded that, for the entire data set, model M1 demonstrated the best predictive capability, while model M2 exhibited the poorest predictive capacity. The results of this data set are presented in Figs. 7 and 8 (for detailed information, refer to Table A4). More details of the percentage of gain of each model regarding the worst model (M2) are given Table A4 in the column RE (%). The percentage of gain of each model regarding the model without using environmental covariates (M0) also are given Table A4 in the last column denoted as RE_M0 (%).

### Comparison between models M3 and M6

The specific comparison between the results of models M3 and M6 illustrates that including only one covariate (${\varvec{X}}_{e.avg})$ summarizing the filtered covariates (${\varvec{X}}_{e}$) can be equal or more efficient than including all the covariates available in ${\varvec{X}}_{e}$. It is important to point out that models M3 and M6 in the predictor contain exactly the same information (See Table 1 for details) but the unique difference is that M3 estimates one beta coefficient for each covariate since all covariates are included in the modeling process, whereas M6 estimates only one beta coefficient (${\varvec{X}}_{e.avg}$; details for how this average was computed are provided in material and methods) of the information available in ${\varvec{X}}_{e}$. In Table 2 we can observe that across data sets by environment the percentage of won models by M6 regarding the worst model was 59.99%, whereas the percentage of won models of M3 regarding the worst model was 51.10%. If analyzed by trait, the won models of M6, regarding the worst model, was 71.67%, but the won models of M3 in the same context was only of 51.11%. Regarding NRMSE across data sets also this was better for model M6 (NRMSE = 4.16) and worse for model M3 (NRMSE = 4.338). For this reason, the gain in RE(%) regarding the worst model was better for model M6 (65.84%) and worse for model M3 (59.41%). This comparison illustrates that parsimonious models can be more efficient.

**Table 1:** Description of the 15 predictors implemented. Environmental covariates (0 denotes not used, whereas 1 denotes used), selection method of environmental covariates (C = Pearson´s correlation and B = Boruta). TC denotes a threshold correlation, and this takes values of 0.3, 0.4, 0.5, 0.6, and 0.7. The largest TC value was evaluated first and if any covariate satisfied this TC value, the second largest was used and so on

Model	Predictor	Environmental covariates	Selection method	Average of covariates	Correlation
M0	${\varvec{K}}_{\varvec{e}}$, ${\varvec{K}}_{\varvec{g}}$, ${\varvec{K}}_{\varvec{g}\varvec{e}}$ and $\varvec{M}$	0	-	0	-
M1	${\varvec{K}}_{\varvec{e}}$, ${\varvec{K}}_{\varvec{g}}$, ${\varvec{K}}_{\varvec{g}\varvec{e}}$, ${\varvec{X}}_{e}$ and $\varvec{M}$	1	-	0	> 0
M2	${\varvec{K}}_{\varvec{e}\varvec{c}}$, ${\varvec{K}}_{\varvec{g}}$, ${\varvec{K}}_{\varvec{g}\varvec{e}\varvec{c}}$and $\varvec{M}$	1	C	0	TC
M3	${\varvec{K}}_{\varvec{e}}$, ${\varvec{K}}_{\varvec{g}}$, ${\varvec{K}}_{\varvec{g}\varvec{e}}$, ${\varvec{X}}_{e}$ and $\varvec{M}$	1	C	0	TC
M4	${\varvec{K}}_{\varvec{e}\varvec{c}}$, ${\varvec{K}}_{\varvec{g}}$, ${\varvec{K}}_{\varvec{g}\varvec{e}\varvec{c}}$, ${\varvec{X}}_{e}$ and $\varvec{M}$	1	C	0	TC
M5	${\varvec{K}}_{\varvec{e}}$, ${\varvec{K}}_{\varvec{g}}$, ${\varvec{K}}_{\varvec{g}\varvec{e}}$,${\varvec{X}}_{e2}$ and $\varvec{M}$	1	C	0	TC
M6	${\varvec{K}}_{\varvec{e}}$, ${\varvec{K}}_{\varvec{g}}$, ${\varvec{K}}_{\varvec{g}\varvec{e}}$, ${\varvec{X}}_{e.avg}$ and $\varvec{M}$	1	C	1	TC
M7	${\varvec{K}}_{\varvec{e}\varvec{c}}$, ${\varvec{K}}_{\varvec{g}}$, ${\varvec{K}}_{\varvec{g}\varvec{e}\varvec{c}}$, ${\varvec{X}}_{e.avg}$ and $\varvec{M}$	1	C	1	TC
M8	${\varvec{K}}_{\varvec{e}\varvec{c}}$, ${\varvec{K}}_{\varvec{g}}$, ${\varvec{K}}_{\varvec{g}\varvec{e}\varvec{c}}$, ${\varvec{X}}_{e.avg}$ and $\varvec{M}$	1	C & B	1	TC
M9	${\varvec{K}}_{\varvec{e}}$, ${\varvec{K}}_{\varvec{g}\varvec{e}}$, ${\varvec{X}}_{\varvec{g}\varvec{e}\varvec{c}}$, $\varvec{M}$,	1	B	0	-
M10	${\varvec{K}}_{\varvec{e}}$, ${\varvec{K}}_{\varvec{g}}$, ${\varvec{K}}_{\varvec{g}\varvec{e}}$, ${\varvec{X}}_{e}$(Tenative true), $\varvec{M}$	1	B	0	-
M11	${\varvec{K}}_{\varvec{e}\varvec{c}}$, ${\varvec{K}}_{\varvec{g}}$, ${\varvec{K}}_{\varvec{g}\varvec{e}\varvec{c}}$, ${\varvec{X}}_{e.avg}$ (Tentative false) and $\varvec{M}$	1	B	1	-
M12	${\varvec{K}}_{\varvec{e}\varvec{c}}$, ${\varvec{K}}_{\varvec{g}}$, ${\varvec{K}}_{\varvec{g}\varvec{e}}$, ${\varvec{X}}_{e.avg}$ (Tenative true) and $\varvec{M}$	1	B	1	-
M13	${\varvec{K}}_{\varvec{e}\varvec{c}}$, ${\varvec{K}}_{\varvec{g}}$, ${\varvec{K}}_{\varvec{g}\varvec{e}\varvec{c}}$, ${\varvec{X}}_{e.avg}$ (Tenative true) and $\varvec{M}$	1	B	1	-
M14	${\varvec{K}}_{\varvec{e}\varvec{c}}$, ${\varvec{K}}_{\varvec{g}}$, ${\varvec{K}}_{\varvec{g}\varvec{e}\varvec{c}}$, ${\varvec{X}}_{e.avg}$ (Tenative False) and $\varvec{M}$	1	B	1	-

**Table 2:** Comparison of models M3 and M6 in terms of count of the number of times these models were better than another in terms of normalized root mean square error (NRMSE), both by environments and by traits. Prediction accuracy was in terms of NRMSE; relative efficiency (RE) in terms of percentage

		Env		Trait			
Data set	Model	Won models	%	Won models	%	NRMSE	RE(%)
Data set 2 (2020)	M3	113	50.22	20	44.44	3.02	43.63
Data set 2 (2020)	M6	140	62.22	40	88.89	3.07	41.31
Data set 3 (2021)	M3	147	46.67	16	35.56	6.89	56.73
Data set 3 (2021)	M6	184	58.41	32	71.11	5.99	80.28
Data set 4 (2022)	M3	138	57.50	30	66.67	2.43	39.60
Data set 4 (2022)	M6	167	69.58	37	82.22	2.12	60.36
Data set 5	M3	435	50.00	26	57.78	5.01	97.67
Data set 5	M6	432	49.66	20	44.44	5.46	81.40
**Across data**	**M3**	**208.25**	**51.10**	**23**	**51.11**	**4.34**	**59.41**
**Across data**	**M6**	**230.75**	**59.97**	**32.25**	**71.67**	**4.16**	**65.84**

## Discussion

Multi-environment genomic prediction presents formidable challenges arising from diverse factors such as genetic variation, genotype-by-environment interaction, environmental heterogeneity, limited training data, and the risks of overfitting and generalization. Collectively, these elements compound the complexity of accurately forecasting genotype performance across different environments. The prediction of tested line performance in novel environments is hindered by sparse data for specific line-by-environment combinations, intricate genotype-by-environment interactions, the impact of environmental variations on performance, limited model stability across environments, and unaccounted factors influencing performance. Successfully navigating these challenges necessitates extensive data collection, the employment of advanced modeling approaches, and a profound understanding of the interplay between genetic and environmental factors. Sustained research efforts are crucial for continual enhancements in the accuracy and reliability of predicting line performance in new and diverse environments.

To enhance the prediction accuracy in challenging scenarios such as tested lines in untested environments (here called leave one environment out) and untested lines in untested environments, the integration of multiple types of input has proven crucial. This has been supported by studies that integrated two types of inputs [11, 12, 14–19, 20], as well as those that incorporated three different sources [7, 21]. Such integration of diverse inputs offers promising avenues for improving prediction accuracy in these challenging scenarios.

In this study, under tested lines in untested environments, we explored the integration of three distinct sources of inputs, namely genomics, phenomics, and environmental information in soft white winter wheat. The results demonstrated that incorporating environmental information alongside genomic and phenomics data led to a substantial increase in prediction accuracy. On average, across various data sets, traits, and environments, the prediction accuracy, as measured by NRMSE, improved by 49.19%. However, it is important to note that the extent of improvement in prediction accuracy varied across the different data sets. For instance, in data set 3 (year 2021), the gain in terms of NRMSE was only 5.68%, whereas in data set 4 (year 2022), it was 60.36%. These findings highlight the diverse impact of incorporating environmental information on prediction accuracy across different data sets and underscore the need for careful consideration of specific data characteristics and contexts in genomic prediction research.

The present study demonstrated a significant improvement in prediction accuracy when incorporating environmental information in addition to genomics and phenomics data. However, it is important to note that the observed gains in prediction accuracy varied across different data sets, suggesting heterogeneity in the results. These discrepancies can be attributed to variations in the quality of the feature selection process and the specific characteristics of each data set. Furthermore, we observed that a naive incorporation of covariates often proved ineffective and, in some cases, even detrimental to prediction accuracy (like model M1 in Table A2; Data set 3 (2021)). Also, we observed that it is not necessary to use all environmental covariates, since many models with variable selection outperformed model M1 that used all environmental covariates (examples are the results of Data set 2 (2020), Data set 3 (2021) and Data set 4 (2022)]. This highlights the importance of careful consideration and thoughtful integration of covariates, as their inclusion can either enhance or diminish the accuracy of predictions.

Concerning the identification of the most crucial environmental covariates for predicting each environment, our findings underscore their dependence on both the specific environment and the corresponding year. For example, for those models that used the Pearson´s correlation to select the optimal covariates, requires for data set 5 (all years together) between 1 and 339 environmental covariates, with an average across environments of 155 environmental covariates. This indicates there is a lot of variability in the number of covariates required for a specific environment, and that only a small average fraction of 5.33% of the environmental covariates for data set 5 (all years together) were required to decrease the prediction error. Details for data set 5 (all years together) of the environmental covariates selected for each environment are provided in Table C1. This observation holds significant weight as it elucidates why the inclusion of all environmental covariates as model inputs often fails to consistently enhance prediction accuracy. Consequently, we consistently observed that conducting feature selection is pivotal for improving the prediction performance of each unique environment.

It is crucial to emphasize that no significant differences were found between the two implemented methods for feature selection, namely Pearson’s Correlation and the Boruta algorithm. However, an exhaustive comparison was not done between the two selection methods since with Boruta, some models (Models M9, 10 and 11) selected not only environmental information but also marker information. Regarding using Pearson’s correlation, a higher threshold for feature selection yielded better results. Nonetheless, a drawback arose as, in many instances, specifying a larger threshold resulted in none of the environmental covariates meeting the criteria for selection. Consequently, Pearson’s correlation proved ineffective in selecting any environmental covariates under these circumstances. On the other hand, the Boruta method presents a distinct advantage by not necessitating a specific threshold and we found that it was slightly better than Pearson’s Correlation (with better performance in three out of the five data sets). This characteristic renders Boruta an exceptionally appealing and efficient tool for variable selection.

Generally, our findings indicate that the inclusion of environmental covariates enhances prediction performance in terms of NRMSE. However, a distinct pattern regarding the superiority of a specific predictor was not clearly discernible. The only consistent trend observed was a slight improvement in predictors that incorporate the average covariate, ${\varvec{X}}_{e.avg}$, as evidenced in the data sets for 2020, 2021, and 2022. The notable advantage of utilizing the average covariate ${\varvec{X}}_{e.avg}$ lies in the requirement for estimating only a single parameter (beta coefficient).

It is crucial to emphasize that our findings align with prior research that demonstrated the beneficial impact of incorporating environmental covariates on enhancing prediction accuracy [23, 24]. However, it should be noted that the extent of improvement in prediction accuracy varies depending on the specific data set and the modeling approach utilized. Also, it is important to point out that the gain found in this research was in terms of NRMSE. We have chosen not to employ the Pearson´s correlation coefficient as a metric for reporting prediction performance, primarily due to the absence of significant improvement associated with this measure (See Table B1 of Annex B), but we are aware that this metric is directly related to the genetic gain of genomic selection [30]. The limited enhancement of using this metric can be ascribed, in part, to our exclusive concentration on feature selection within the domain of environmental covariates. Also, it can be attributed to the fact that the environmental covariates were assessed not at the genotype level but rather at the environmental (location) level.

## Conclusions

In this research, we employed a cross-validation scheme, partially tested lines and untested environment, to assess the benefits of integrating environmental covariates, in addition to the already integrated genomics and phenomics information. Our objective was to evaluate the impact of this integration on prediction accuracy. Our findings indicate that the inclusion of environmental information resulted in a notable increase of 49.19% in the prediction accuracy, as measured by the normalized root mean square error across multiple data sets. Among the four data sets examined in our study, all of them demonstrated improved prediction accuracy when environmental information was integrated. Notably, data set 3 exhibited the smallest gain, with an increase of only 5.68%. Conversely, data set 4 from the year 2022 showcased the largest gain, with a substantial increase of 60.36%. These results provide empirical evidence supporting the notion that incorporating additional inputs into the modeling process holds significant potential for enhancing prediction accuracy. However, it is crucial to approach the integration of environmental covariates with care, as naive integration often proves unhelpful. Therefore, we recommend the use of feature selection techniques, such as Pearson’s correlation and Boruta, to ensure an optimal or near-optimal integration. By employing these techniques, a more refined and effective integration of environmental covariates is guaranteed.

## Methods

### Data set descriptions

Data sets 1 to 5, referred to as the wheat data, were utilized in this study and are the same used in the paper “Genomics combined with UAS data enhances prediction of grain yield in winter wheat” by Montesinos-López et al. [15]; they were used for the GY trait, in addition to plant high (PH) and days to heading (HD). The wheat lines used in the study were obtained from the breeding program of Washington State University (WSU) and were cultivated at different locations within the state of Washington (see supplementary Figure S1). Below is a summary of the characteristics of each data set:

Data set 1, Wheat_1 (Year 2019): Is comprised of 1,379 distinct wheat lines evaluated across two environments, namely Kincaid and Pullman. The data set contains a total of 1,379 observations with no replication of lines in multiple environments.

Data set 2, Wheat_2 (Year 2020): Consists of 758 unique wheat lines assessed in six different environments, namely Farmington, Harrington, Kincaid, Lind, Ritzville, and Walla Walla. The data set contains a total of 952 observations due to the presence of repeated lines across multiple environments.

Data set 3, Wheat_3 (Year 2021): Includes 452 distinct wheat lines evaluated in eight environments, namely Davenport, Harrington, Kahlotus, Kincaid, Lind, Pullman, Ritzville, and Walla Walla. The data set contains a total of 780 observations due to the inclusion of certain lines in multiple environments.

Data set 4, Wheat_4 (Year 2022): Is comprised of 363 unique wheat lines assessed in six environments, namely Davenport, Farmington, Harrington, Prescott, Pullman, and Ritzville. The data set contains a total of 483 observations due to the presence of repeated lines across multiple environments.

Data set 5, Wheat_5 (Joint information of years 2019–2022): Is comprised of 2279 unique wheat lines assessed in twenty environments, namely 2019_Kincaid, 2019_ Pullman, 2020_Farmington, 2020_ Harrington, 2020_ Kincaid, 2020_ Ritzville, 2020_ Walla_Walla, 2021_Davenport, 2021_ Harrington, 2021_ Kahlotus, 2021_ Kincaid, 2021_ Pullman, 2021_ Ritzville, 2021_ Walla_Walla, 2022_Davenport, 2022_ Farmington, 2022_ Harrington, 2022_ Prescott, 2022_ Pullman, 2022_ Ritzville. The data set contains a total of 3891 observations due to the presence of repeated lines across multiple environments and the environment results of the year/location combinations.

To collect phenotypic data, the Sentera Quad Multispectral Sensor (Sentera, St Paul, MN) was employed. This sensor encompasses four sensors that cover a total of eight broad spectral bands ranging from 450 nm to 970 nm, which, based on previous research, are relevant for evaluating winter wheat in Washington. An unmanned aerial system (DJI Inspire 1) equipped with the Sentera camera flew along a predetermined route at an altitude of 45 m, capturing georeferenced images with 85% overlapping coverage. UAS data was collected within a four-hour window of solar noon, with an effort to be as close to solar noon as possible. Flights often took 20 min to collect and were done on days with clear skies to limit variability in solar radiation. Data were collected on wheat plants between the heading and flowering (Feekes 10.1 and 10.5) growth stage. The collected UAS images were processed in Pix4Dmapper (Pix4D Inc., Denver, CO) to create a single orthomosaic image for each sensor per location. These orthomosaic images were then transferred to the Geographic Information System (QGIS) for plot identification and subsequently subjected to further processing using a custom R code. This processing involved image calibration, index calculation, and extraction of mean data for individual plots.

For radiometric calibration in 2019, a single reflectance panel with 85% reflectance was employed for the RGB and red edge bands. The NIR band was normalized using a coefficient of 3.07 during the calculation of spectral reflectance indices (SRIs), according to the formula: NIR = (2.921 × Blue) - (0.754 × Red). In the years 2020 to 2022, a set of calibration panels consisting of five panels with reflectance ranging from 2 to 85% (MosaicMill Oy, Vantaa, Finland) was used. All raw band layers were adjusted based on the relationship: SR = DN × Slope ± Intercept, where the slope and intercept were derived from the regression of observed reflectance in calibration panels. In this equation, DN represents the raw observed pixel values, and SR represents the true reflectance value. Adjusted multispectral band values were utilized in all data sets to calculate indices for subsequent model analysis.

The genotyping process employed genotyping-by-sequencing (GBS; [31]) to analyze all the wheat lines. Initially, the original data set consisted of a total of 6,075,743 single nucleotide polymorphisms (SNPs). However, the data set underwent a series of filtering steps to refine the SNPs for further analysis. The filtering criteria included removing SNPs with homozygosity greater than 80%, less than 50% missing data, a minor allele frequency greater than 0.05, and heterozygosity less than 5%. Following these filtering steps, the data set was reduced to 19,645 SNPs, which met the specified criteria.

To address missing data in the markers, imputation was performed using the ‘expectation-maximization’ algorithm implemented in the ‘R’ package rrBLUP [32]. This imputation process helped to fill in the missing values within the data set. Within each data set, the best linear unbiased estimates (BLUEs) were computed utilizing two experimental designs (alpha lattice design and augmented randomized complete block design). These designs were utilized to obtain reliable and unbiased estimates for further analysis. For details on the BLUEs computation see Montesinos-López et al. [15].

In addition to the genomic and phenomics (UAS data), we collected environmental information for each environment. The environmental covariates measured in each one are given in Table 3.

**Table 3:** Description of environmental covariates (EC) used in each environment

No.	EC abbreviation	EC full name
1	Air MinA°F	Minimum air temperature in °F
2	Air AvgA°F	Average air temperature over the 24 h in °F
3	Air MaxA°F	Maximum air temperature in °F
4	Avg1.5 m DPA°F	Average dewpoint temperature in °F at 1.5 m height
5	Avg1.5 m RH%	Average relative humidity at 1.5 m height
6	Soil 2 in.A°F	Soil temperature at two inches depth in °F
7	Soil MinA°F	Minimum soil temperature at eight inches depth in °F
8	Soil AvgA°F	Average soil temperature at eight inches depth in °F
9	Avg 2 in.SWPkPa	Average stem water potential at two inches depth
10	Avg 8 in.SWPkPa	Average stem water potential at eight inches depth
11	TotPrecin	Total precipitation in inches
12	TotalSolarRadMJ/mA^2^	Total solar radiation for a 24 h period
13	EToin	Evapotranspiration from the soil in inches with reference to grass
14	ETrin	Evapotranspiration from the soil in inches with reference to alfalfa

In each environment, all the covariates given in Table 3 were measured daily from the date of planting of each trial location until the date of harvest. For this reason, for each environment there were available 2904 records since each covariate was measured daily, and on average these covariates were measured across 207 days. Data were downloaded from the WSU AgWeatherNet system of weather stations (www.weather.wsu.edu) using the weather station that was closest to the trial location.

### Feature selection methods

We implemented two feature selection methods exactly as was done in the study of Montesinos-López et al. [10]. The first feature selection method involved determining the correlation between the environmental covariates and the response variable. The selection process identified the highest correlation within each training set for each trait. However, it is crucial to note that this selection of covariates is carried out without considering the response variables in the testing set. In other words, the covariates corresponding to the environment being predicted are not included. The threshold correlations used for selecting environmental covariates were 0.3, 0.4, 0.5, 0.6, and 0.7. When the correlations fall below the 0.3 value, it indicates that the training process was performed without any environmental covariates. However, if only a few covariates met the threshold correlation of 0.7, only those covariates were used in the training process. If no covariates satisfied this threshold, the ones meeting the lower threshold (0.5) were used, and so on.

The second feature selection method employed the Boruta algorithm, which aims to identify covariates that are either strongly or weakly relevant to the response variable. In this case, the covariates included in the training process of the models were determined using only the response variables from the training set. The observations that form part of the testing set were not utilized for selecting the significant environmental covariates.

Boruta is an algorithm specifically designed for feature selection in high-dimensional data sets with noisy features [33]. It operates by creating a shadow feature set, which is a replica of the original feature set with randomly permuted values. These shadow features serve as a control to assess the statistical significance of the original features. The relevance of the original features is determined based on whether their importance scores significantly exceed the importance scores of their corresponding shadow features. Boruta is efficient in data sets containing numerous noisy features, where traditional feature selection methods may encounter challenges. However, it can be computationally intensive and may require careful parameter tuning to achieve optimal outcomes [33].

The Boruta algorithm operates through the following steps:

Step 1. Generate a shadow feature set by randomly permuting the values of each feature.

Step 2. Train a random forest model using both the original feature set and the shadow feature set.

Step 3. Calculate the feature importance scores for each original feature by comparing them to the importance scores of their corresponding shadow features.

Step 4. Determine the maximum importance score for each feature.

Step 5. Employ the Binomial test to assess the statistical significance of each feature. If it is deemed significant, the feature is marked as important; otherwise, it is marked as unimportant. The Binomial test is a statistical test utilized in Boruta to evaluate the significance of feature importance scores. It compares the observed number of successes (e.g., the number of times a feature’s importance score exceeds a threshold) with the expected number of successes under a null hypothesis. This test determines whether the observed results are statistically significant or can be attributed to chance. In Boruta, the Binomial test is employed to determine if the feature importance scores are significantly higher than the importance scores of the shadow features, indicating the importance of the original features [33].

Step 6. Repeat steps 1–5 for a predetermined number of iterations.

Step 7. Rank the features based on their importance scores and select the top n features for the final feature set. In Boruta, features are categorized as “Confirmed” if they are considered important, “Rejected” if they are deemed unimportant, and “Tentative” if they require further investigation or are considered less important.

### Bayesian model

The Bayesian model used with all predictors given in Table 1 is

1 $$\eqalign{{Y_{ij}}\, =  & \mu \, + \,{E_i}\, + \,{g_j}\, +  \cr & g{E_{ij}}\, + \,\sum\limits_{k = 1}^r {{X_{ik}}{\beta _k}} \, + \,\sum\limits_{l = 1}^3 {{M_{ijl}}{\beta _{M,l}}} \, + \,{ \in _{ij}} \cr} $$

Where $$ {Y}_{ij}$$ is the response variable for the genotype j in environment i, $$ \mu $$ is a general mean, $$ {E}_{i}$$ are the random effects of locations (environments) distributed as $$ \varvec{E}={\left({E}_{1},\dots ,{E}_{I}\right)}^{T}\sim N\left(0,{\sigma }_{E}^{2}{\varvec{H}}_{\varvec{e}}\right)$$, where $$ {\varvec{H}}_{\varvec{e}}$$ is the environmental relationship matrix as computed by [34], but instead of using genomic information, it was computed using environmental variables; that is, $$ {\varvec{H}}_{\varvec{e}}=\frac{{\varvec{X}}_{e}{\varvec{X}}_{e}^{T}}{r}$$, where $$ {\varvec{X}}_{e}=\left({X}_{1},\dots ,{X}_{r}\right)$$ is the standardize (centered and scaled) matrix of dimension $$ I\times r$$ containing the environmental information of $$ I$$ environments and for each environment were measured $$ r$$ environmental covariates; $$ {X}_{ik}$$ denotes the environmental covariate k measured in environment i, $$ {\beta }_{k}$$ is the beta coefficient corresponding to covariate $$ {X}_{ik}$$; $$ {g}_{j},j=1,\dots ,J$$, are the random effects of genotypes (lines), $$ g{E}_{ij}$$ are the random effects of genotype$$ \times $$environment interaction (GE) and $${ \in _{ij}}$$ are the random error components in the model assumed to be independent normal random variables with mean 0 and variance $$ {\sigma }^{2}$$. Furthermore, it is assumed that $$ \varvec{g}={\left({g}_{1},\dots ,{g}_{J}\right)}^{T}\sim N\left(0,{\sigma }_{g}^{2}{\varvec{K}}_{\varvec{g}}\right)$$, where $$ {\varvec{K}}_{g}$$ is the genomic relationship matrix as computed by [34], which is slightly different than what [35] proposed, using the marker data ($$ {\varvec{K}}_{g}=\frac{{\varvec{M}}_{e}{\varvec{M}}_{e}^{T}}{p})$$ where $$ {\varvec{M}}_{e}$$ is the standardize (centered and scaled) matrix of dimension $$ J\times p$$ containing the marker information of $$ J$$ genotypes for which $$ p$$ markers were measured. $$gE = {\left( {g{E_{11}}, \ldots ,g{E_{1J}}, \ldots ,g{E_{IJ}}} \right)^T} \sim N\left( {{\bf{0}},{K_{gec}}\sigma _{gE}^2} \right)$$, where $${K_{gec}} = {K_{ec}} \odot {Z_g}{K_g}Z_g^T$$, where $$ {\varvec{K}}_{\varvec{e}\varvec{c}}={\varvec{Z}}_{e}{\varvec{H}}_{\varvec{e}}{\varvec{Z}}_{e,}^{T}{\varvec{Z}}_{e}$$ is the design matrix of environments, $${\odot }$$ denotes the Hadamard product and $$ {\varvec{Z}}_{g}$$ is the design matrix of genotypes. It is important to point out that the dimension of $$ {\varvec{X}}_{e}$$ is reduced after variable selection and in place of being $$ I\times r$$, it is $$ I\times {r}_{s}$$ with $$ {r}_{s}\le r$$. $$ {M}_{ijl}$$ denotes the $$ lth$$ multispectral index, with $$ l=\text{1,2},3$$ computed from the multispectral information for the j*th* line and i*th* environment and $$ {\beta }_{M,l}$$ denotes the beta coefficient corresponding to the $$ lth$$ multispectral index. In vector notation the information of the three multispectral index (UAS data) is denoted as $$ \varvec{M}$$; with $$ {\varvec{M}}_{ij}=[{M}_{11},\dots ,{M}_{qJ},\dots ,{M}_{IJ}]$$; $$ {\varvec{M}}_{ij}=[{M}_{ij1,}{M}_{ij2},{M}_{ij3}]$$ and $$ {\beta }_{M}=[{\beta }_{M,1}{\beta }_{M,2},{\beta }_{M,3}]$$. It is crucial to highlight that the efficiency of incorporating replicated lines in each environment is a noteworthy aspect, particularly in relation to how design matrices and linear kernels are computed for all implemented predictors. For beta coefficients ($$ {\beta }_{k}$$ and $$ {\beta }_{M,l}$$) a prior assumed independent and identically normal distribution, with mean zero and variance $$ {\sigma }_{{\beta }_{k} }^{2}$$(or $$ {\sigma }_{{\beta }_{M,l} }^{2})$$ were used. For the implementation of model (1) we used the BGLR R library of [36] where terms with these priors for the beta coefficients are specified in model as Bayesian Ridge Regression (BRR), whereas for the remaining components of model (1) each component is specified as model RKHS, where RKHS stands for Reproducing Kernel Hilbert Spaces. Since the implementation was done under a Bayesian framework all terms in the predictor are assumed random variables.

### Predictors implemented

It should be noted that among the models, only Model M0 does not utilize environmental covariates, while the remaining models employ all environmental covariates or a subset of these covariates. All implemented predictors are given in Table 1.

To provide a better understanding of the contents of Table 1, we will describe the computation of certain predictor components. For example, ${\varvec{K}}_{\varvec{e}}$ is calculated as ${\varvec{K}}_{\varvec{e}}=\frac{{\varvec{Z}}_{e}{\varvec{Z}}_{e}^{T}}{I}$, ${K_{ge}} = {K_{e}} \odot {Z_g}{K_g}Z_g^T$, and ${\varvec{X}}_{e.avg}$ represents an average covariate derived from the environmental covariates (${\varvec{X}}_{e}$). When the feature selection is not applied, ${\varvec{X}}_{e}$ includes all available environmental covariates. However, when the feature selection is applied, only the selected covariates are included. The average covariate, ${\varvec{X}}_{e.avg}$, is computed from ${\varvec{X}}_{e}$ with an order of $I\times {r}_{s}$ after variable selection. The computation of ${\varvec{X}}_{e.avg}$ involves the following steps:

1. Determine the correlation direction (positive or negative) of each column of ${\varvec{X}}_{e}$ using only the training set.
2. Multiply the columns of ${\varvec{X}}_{e}$ with a negative correlation by -1 to ensure a positive correlation with the response variable. The resulting matrix is denoted as ${\varvec{X}}_{e}^{*}$.
3. Compute ${\varvec{X}}_{e.avg}$ for the entire data set by taking the average of each row of ${\varvec{X}}_{e}^{*}.$ As a result, ${\varvec{X}}_{e.avg}$ has an order of $I\times 1$. However, since the covariates are measured at the environment (location) level, ${\varvec{X}}_{e.avg}$ is expanded to an order of $IJ\times 1$, where each covariate is the same for all lines within the same environment.

By using $$ {\varvec{X}}_{e.avg}$$ as a single covariate, only one beta coefficient needs to be estimated instead of the $$ {r}_{s}$$ beta coefficients required when using the $$ {\varvec{X}}_{e}$$ matrix as an input. In predictor M9, it is important to note that $$ {\varvec{X}}_{\varvec{g}\varvec{e}\varvec{c}} $$represents the selected covariates, but instead of selecting only from the environmental covariates, the Boruta selection was performed on both markers and environmental covariates together. Pearson’s correlation and the Boruta method were used for feature selection, which will be explained in the following section. All predictors presented in Table 1 were implemented using the BGLR package by Pérez and de los Campos [36] in the R statistical software [37].

The training of each model differs in terms of the environmental covariates included in each data set. Therefore, Model M0 stands apart from the other models as it makes predictions without incorporating any information from the environmental covariates. As a result, the linear kernels ${\varvec{K}}_{\varvec{e}}=\frac{{\varvec{Z}}_{e}{\varvec{Z}}_{e}^{T}}{I}$ and ${K_{ge}} = {K_{ec}} \odot {Z_g}{K_g}Z_g^T$ were computed only with the design matrices of environments (${\varvec{Z}}_{e}$). On the other hand, Model M1 is the same as Model M0 but includes all available environmental information as covariates (${\varvec{X}}_{e}$) without variable selection. Model M2 is similar to Model M0, but the computation of linear kernels (${\varvec{K}}_{\varvec{e}\varvec{c}}={\varvec{Z}}_{e}{\varvec{H}}_{\varvec{e}}{\varvec{Z}}_{e}^{T}$and ${K_{gec}} = {K_{ec}} \odot {Z_g}{K_g}Z_g^T$ takes into account the environmental covariates after variable selection using Pearson’s correlation.

Model M3 is equivalent to Model M1, but it uses the covariates ${\varvec{X}}_{e}$ after variable selection with Pearson’s correlation. Model M4 is identical to model M2, but it also incorporates environmental information as covariates (${\varvec{X}}_{e}$) following variable selection with Pearson’s correlation. Model M5 is similar to model M3, but instead of solely utilizing ${\varvec{X}}_{e}$ as covariates after variable selection with Pearson’s correlation, it also includes the square of each column of ${\varvec{X}}_{e}$ as covariates (${\varvec{X}}_{e2}={\varvec{X}}_{e}+{\varvec{X}}_{e}*{\varvec{X}}_{e}$). Model M6 is analogous to model M3, but instead of employing ${\varvec{X}}_{e}$ as a covariate after variable selection with Pearson’s correlation, it only employs the average covariate (${\varvec{X}}_{e.avg}$). Model M7 is equivalent to model M4, except that it incorporates the average covariate (${\varvec{X}}_{e.avg}$) instead of ${\varvec{X}}_{e}$ as a covariate after variable selection using Pearson’s correlation. Model M8 is identical to model M7, except that the variable selection process was conducted simultaneously using both Pearson’s correlation and Boruta. Model M9 performed variable selection of markers and environmental covariates simultaneously using the Boruta algorithm, resulting in the selected covariates referred to as ${\varvec{X}}_{\varvec{g}\varvec{e}\varvec{c}}$, while ${\varvec{K}}_{\varvec{e}}$and ${\varvec{K}}_{\varvec{g}\varvec{e}}$ were computed solely using the design matrix of the environment (${\varvec{Z}}_{e}$). Model M10 is similar to model M3, but the selection of environmental covariates was accomplished using the Boruta algorithm, which selected both tentative and confirmatory covariates. It is important to note that in model M10, the Boruta algorithm was also applied to select markers, and subsequently, the linear kernels of lines (${\varvec{K}}_{\varvec{g}}$) and genotype by environment (${\varvec{K}}_{\varvec{g}\varvec{e}}$) interactions were computed using the selected markers. Model M11 is equivalent to model M8, but the selection of environmental and marker covariates was performed exclusively using the Boruta algorithm, selecting only confirmatory covariates. Model M12 is identical to model M11, except that the Boruta algorithm was utilized to select both tentative and confirmatory covariates. Model M13 is similar to model M12, but the selected environmental covariates were also employed to compute the linear kernels of environments (${\varvec{K}}_{\varvec{e}\varvec{c}}$) and genotype by environment (**K**.gec) interactions. Finally, model M14 is equivalent to model M13, but only confirmed features were selected using the Boruta algorithm. For further details on each predictor, please refer to Table 1. Even though certain predictors used similar information, we evaluated them since some predictors used this information as covariates (with particular priors) and in other cases were used as linear kernels under the assumption of random effects. Since we made feature selection of markers and environmental covariates with the Boruta algorithm in some models (M9, M10 and M11), the results of these models in which feature selection was performed with Pearson´s correlation (models M2-M7) were not directly compared.

### Assessment of predictive performance

To evaluate the accuracy of predictions, a leave-one-environment-out (LOEO) cross-validation approach was utilized for each data set. This cross-validation strategy is important when breeders are interested in predicting phenotypes (or breeding values) of all genotypes under study in a complete environment. For this reason, this cross-validation is very challenging due to the fact that we want to predict performance of all genotypes in a new or untested environment with no available information in the training set. It is important to point out that LOEO cross-validation is considerably more difficult than when we use a cross-validation for tested lines in tested environments (also called CV2 Cross validation [38–39]) since this LOEO cross validation is for tested lines in untested environments (also called CV0 cross validation [38, 39]). The LOEO strategy involved iteratively constructing the training set by excluding one environment (testing set) while utilizing the remaining environment as the training set. The evaluation methodology adhered to the approach is described by Montesinos-López et al. [40] with more details. However, it should be noted that the selection of environmental covariates mentioned in Table 1 was performed after splitting the data into training and testing sets. Only the training set was used for selecting the important covariates. This approach was adopted to avoid data leakage, which occurs when the data used to train a machine learning algorithm contains information that the model is trying to predict. This leakage of information is a primary error in machine learning and can greatly impact the performance and validation accuracy of the model. Utilizing the entire data set before splitting it into training and testing sets leads to overly optimistic results that may not translate well into real-world applications. Also, it is important to point out that the UAS information was not included in the test data.

The prediction accuracy was measured using the Normalized Root Mean Squared Error (NRMSE). Additionally, we conducted a computation to determine the number of instances where model m outperformed model m’ in terms of NRMSE, considering m = 0,…,14 and m’ = 0,…,14, with m being different from m’. This count was performed for each data set, taking into consideration the specific traits and environments being evaluated. Furthermore, we calculated the Relative Efficiency (RE) of each model relative to the worst model, using the following expression:

$$ RE=\left(\frac{NRMSE\left({M}_{i}\right)}{NRMSE\left({M}_{k}\right)}-1\right)\times 100$$

Let $$ {M}_{i}$$ represent any of the models, where i ranges from 0 to 14, within each data set. Similarly, $$ {M}_{k}$$ represents the model with the highest NRMSE value among all models in a specific data set.

### Electronic supplementary material

Below is the link to the electronic supplementary material.

Supplementary Material 1

## Data Availability

Data used in this manuscript can be found at: 10.7273/000005294.

## Abbreviations

BLUEs: Best linear unbiased estimates
EC: Environmental covariates
LOEO: Leave-one-environment-out
GS: Genomic selection
GBS: Genotyping-by-sequencing
GY: Grain yield
HD: Heading date
PH: Plant height
NRMSE: Normalized root mean squared error
RE: Relative efficiency
SNPs: Single nucleotide polymorphisms
SRIs: Spectral reflectance indices
UAS: Unmanned aerial systems
