# Supplementary material for: Enhancing winter wheat prediction with genomics, phenomics and environmental data
Source: BMC Genomics. 2024 May 31;25:544. doi: 10.1186/s12864-024-10438-4 (PMC11143639; doi:10.1186/s12864-024-10438-4)

Appendix A

Table A1. Data set 2 (2020). Count of the number of times a model was better than another in terms of normalized root mean square error (NRMSE), both by environments and by traits. Prediction accuracy in terms of NRMSE. Relative efficiency (RE) in percentage, while RE_M0 is the relative efficiency in percentage regarding model M0.

**Model**	**Env**		**Trait**		**NRMSE**	**RE (%)**	**RE_M0 (%)**
	**Won models**	**%**	**Won models**	**%**			
M0	63	28.00	0	0.00	4.34	0.00	0.00
M1	113	50.22	25	55.56	3.91	11.02	11.02
M2	124	55.11	28	62.22	2.97	45.89	45.89
M3	113	50.22	20	44.44	3.02	43.63	43.63
M4	110	48.89	27	60.00	2.89	50.06	50.06
M5	119	52.89	21	46.67	4.11	5.56	5.56
M6	140	62.22	40	88.89	3.07	41.31	41.31
M7	144	64.00	40	88.89	2.82	53.89	53.89
M8	90	40.00	20	44.44	4.01	8.28	8.28
M9	72	32.00	11	24.44	4.29	1.06	1.06
M10	73	32.44	7	15.56	3.86	12.31	12.31
M11	94	41.78	16	35.56	3.60	20.47	20.47
M12	94	41.78	16	35.56	3.60	20.47	20.47
M13	98	43.56	19	42.22	3.48	24.71	24.71
M14	98	43.56	19	42.22	3.48	24.71	24.71

Table A2. Data set 3 (2021). Count of the number of times a model was better than another in terms of normalized root mean square error (NRMSE), both by environments and by traits. Prediction accuracy in terms of NRMSE. Relative efficiency (RE) in percentage, while RE_M0 is the relative efficiency in percentage regarding model M0.

**Model**	**Env**		**Trait**		**NRMSE**	**RE (%)**	**RE_M0 (%)**
	**Won models**	**%**	**Won models**	**%**			
M0	147	46.67	28	62.22	6.33	70.58	0.00
M1	140	44.44	22	48.89	6.82	58.22	-7.18
M2	73	23.17	2	4.44	10.80	0.00	-41.39
M3	147	46.67	16	35.56	6.89	56.73	-8.13
M4	152	48.25	9	20.00	6.78	59.22	-6.64
M5	112	35.56	10	22.22	8.31	29.91	-23.83
M6	184	58.41	32	71.11	5.99	80.28	5.68
M7	105	33.33	2	4.44	9.02	19.75	-29.82
M8	146	46.35	28	62.22	6.39	68.85	-0.94
M9	159	50.48	25	55.56	6.21	73.79	1.93
M10	142	45.08	20	44.44	7.00	54.30	-9.57
M11	174	55.24	29	64.44	6.11	76.79	3.60
M12	174	55.24	29	64.44	6.11	76.79	3.60
M13	158	50.16	26	57.78	6.33	70.48	0.00
M14	161	51.11	33	73.33	6.03	79.21	4.98

Table A3. Data set 4 (2022). Count of the number of times a model was better than another in terms of normalized root mean square error (NRMSE), both by environments and by traits. Prediction accuracy in terms of NRMSE. Relative efficiency (RE) in percentage, while RE_M0 is the relative efficiency in percentage regarding model M0.

Model	Env		Trait		NRMSE	RE (%)	RE_M0 (%)
	Won models	%	Won models	%			
M0	63	26.25	9	20.00	3.40	0.00	0.00
M1	135	56.25	21	46.67	2.66	27.77	27.77
M2	170	70.83	37	82.22	2.26	50.08	50.08
M3	138	57.50	30	66.67	2.43	39.60	39.60
M4	136	56.67	28	62.22	2.41	40.95	40.95
M5	130	54.17	28	62.22	2.59	31.00	31.00
M6	167	69.58	37	82.22	2.12	60.36	60.36
M7	163	67.92	34	75.56	2.14	58.40	58.40
M8	113	47.08	21	46.67	2.82	20.51	20.51
M9	80	33.33	11	24.44	3.07	10.58	10.58
M10	87	36.25	17	37.78	2.78	22.17	22.17
M11	69	28.75	12	26.67	2.93	15.88	15.88
M12	69	28.75	12	26.67	2.93	15.88	15.88
M13	64	26.67	6	13.33	3.26	4.14	4.14
M14	64	26.67	6	13.33	3.26	4.14	4.14

Table A4. Data set 5 (all years together). Count of the number of times a model was better than another in terms of normalized root mean square error (NRMSE), both by environments and by traits. Prediction accuracy in terms of NRMSE. Relative efficiency (RE) in percentage regarding the worst model, while RE_M0 is the relative efficiency in percentage regarding model M0.

Model	Env		Trait		NRMSE	RE (%)	RE_M0 (%)
	Won models	%	Won models	%			
M0	401	46.09	24	53.33	6.49	52.77	0.00
M1	542	62.30	40	88.89	3.67	170.26	76.84
M2	276	31.72	3	6.67	9.91	0.00	-34.51
M3	435	50.00	26	57.78	5.01	97.67	29.54
M4	306	35.17	10	22.22	7.85	26.23	-17.32
M5	420	48.28	17	37.78	6.36	55.80	2.04
M6	432	49.66	20	44.44	5.46	81.40	18.86
M7	319	36.67	12	26.67	8.73	13.48	-25.66
M8	462	53.10	23	51.11	5.13	93.13	26.51
M9	415	47.70	25	55.56	6.17	60.72	5.19
M10	403	46.32	23	51.11	7.31	35.60	-11.22
M11	437	50.23	21	46.67	6.53	51.86	-0.61
M12	311	35.75	13	28.89	9.61	3.10	-32.47
M13	466	53.56	23	51.11	4.74	109.03	36.92
M14	466	53.56	22	48.89	4.89	102.82	32.72

Appendix B.

Table B1. Prediction accuracy for each model across environment and traits for each data set in terms of Average Pearson´s Correlation (APC).

Data	Data set 2 (2020)	Data set 3 (2021)	Data set 4 (2022)	Data set 5 (all years together)
Model	APC	APC	APC	APC
M0	0.611	0.724	0.491	0.491
M1	0.585	0.723	0.509	0.509
M10	0.594	0.712	0.196	0.196
M11	0.611	0.716	0.479	0.479
M12	0.611	0.716	0.479	0.479
M13	0.628	0.724	0.499	0.499
M14	0.628	0.725	0.499	0.499
M2	0.602	0.686	0.502	0.502
M3	0.585	0.724	0.502	0.502
M4	0.585	0.673	0.519	0.519
M5	0.603	0.724	0.489	0.489
M6	0.623	0.724	0.505	0.505
M7	0.608	0.668	0.510	0.510
M8	0.627	0.717	0.512	0.512
M9	0.602	0.627	0.223	0.223

Appendix C

Table C1. Environmental covariates selected with Pearson´s correlation criteria for each trait and environment for Data set 5 (all years together). Env_Cov_Sel denotes the environmental covariates selected. No_Env_Sel denotes the number of environmental covariates selected out of the 2904 environmental covariates available.

Trait	Env	No_Env_Sel	Env_Cov_Sel
GY	2019_Kincaid	103	Min.F_12/13
GY	2019_Kincaid	103	Min.F_02/12
GY	2019_Kincaid	103	Min.F_04/05
GY	2019_Kincaid	103	Min.F_04/09
GY	2019_Kincaid	103	Min.F_04/11
GY	2019_Kincaid	103	Min.F_04/26
GY	2019_Kincaid	103	Min.F_05/06
GY	2019_Kincaid	103	Min.F_05/09
GY	2019_Kincaid	103	Min.F_06/20
GY	2019_Kincaid	103	Min.F_06/27
GY	2019_Kincaid	103	Min.F_06/28
GY	2019_Kincaid	103	Avg.F_12/14
GY	2019_Kincaid	103	Avg.F_01/19
GY	2019_Kincaid	103	Avg.F_03/18
GY	2019_Kincaid	103	Avg.F_06/21
GY	2019_Kincaid	103	Avg.F_06/27
GY	2019_Kincaid	103	Avg.F_07/08
GY	2019_Kincaid	103	Max.F_10/23
GY	2019_Kincaid	103	Max.F_12/14
GY	2019_Kincaid	103	Max.F_06/21
GY	2019_Kincaid	103	Max.F_06/22
GY	2019_Kincaid	103	Max.F_06/27
GY	2019_Kincaid	103	Max.F_07/02
GY	2019_Kincaid	103	Max.F_07/08
GY	2019_Kincaid	103	Avg1.5m.DP.F_03/30
GY	2019_Kincaid	103	Avg1.5m.DP.F_03/31
GY	2019_Kincaid	103	Avg1.5m.DP.F_04/07
GY	2019_Kincaid	103	Avg1.5m.DP.F_04/08
GY	2019_Kincaid	103	Avg1.5m.DP.F_04/09
GY	2019_Kincaid	103	Avg1.5m.DP.F_04/11
GY	2019_Kincaid	103	Avg1.5m.DP.F_04/19
GY	2019_Kincaid	103	Avg1.5m.DP.F_04/20
GY	2019_Kincaid	103	Avg1.5m.DP.F_04/23
GY	2019_Kincaid	103	Avg1.5m.DP.F_05/16
GY	2019_Kincaid	103	Avg1.5m.DP.F_05/28
GY	2019_Kincaid	103	Avg1.5m.DP.F_05/31
GY	2019_Kincaid	103	Avg1.5m.RH._11/23

GY	2019_Kincaid	103	Avg1.5m.RH._12/12
GY	2019_Kincaid	103	Avg1.5m.RH._01/16
GY	2019_Kincaid	103	Avg1.5m.RH._01/18
GY	2019_Kincaid	103	Avg1.5m.RH._03/29
GY	2019_Kincaid	103	Avg1.5m.RH._03/30
GY	2019_Kincaid	103	Avg1.5m.RH._03/31
GY	2019_Kincaid	103	Avg1.5m.RH._04/03
GY	2019_Kincaid	103	Avg1.5m.RH._04/06
GY	2019_Kincaid	103	Avg1.5m.RH._04/13
GY	2019_Kincaid	103	Avg1.5m.RH._04/20
GY	2019_Kincaid	103	Avg1.5m.RH._05/16
GY	2019_Kincaid	103	Avg1.5m.RH._05/17
GY	2019_Kincaid	103	Avg1.5m.RH._05/24
GY	2019_Kincaid	103	Avg1.5m.RH._05/26
GY	2019_Kincaid	103	Avg1.5m.RH._06/08
GY	2019_Kincaid	103	Avg1.5m.RH._06/21
GY	2019_Kincaid	103	Avg1.5m.RH._06/22
GY	2019_Kincaid	103	Avg1.5m.RH._06/26
GY	2019_Kincaid	103	Avg1.5m.RH._06/27
GY	2019_Kincaid	103	Avg1.5m.RH._06/28
GY	2019_Kincaid	103	Avg1.5m.RH._07/02
GY	2019_Kincaid	103	Min.F.1_06/28
GY	2019_Kincaid	103	Avg.F.1_06/28
GY	2019_Kincaid	103	TotPrecin_12/23
GY	2019_Kincaid	103	TotPrecin_04/05
GY	2019_Kincaid	103	TotPrecin_04/06
GY	2019_Kincaid	103	TotalSolarRadMJ.m._10/28
GY	2019_Kincaid	103	TotalSolarRadMJ.m._12/08
GY	2019_Kincaid	103	TotalSolarRadMJ.m._12/12
GY	2019_Kincaid	103	TotalSolarRadMJ.m._01/12
GY	2019_Kincaid	103	TotalSolarRadMJ.m._03/09
GY	2019_Kincaid	103	TotalSolarRadMJ.m._03/27
GY	2019_Kincaid	103	TotalSolarRadMJ.m._05/09
GY	2019_Kincaid	103	TotalSolarRadMJ.m._05/26
GY	2019_Kincaid	103	TotalSolarRadMJ.m._06/27
GY	2019_Kincaid	103	TotalSolarRadMJ.m._07/02
GY	2019_Kincaid	103	EToin_12/12
GY	2019_Kincaid	103	EToin_12/13
GY	2019_Kincaid	103	EToin_01/16
GY	2019_Kincaid	103	EToin_01/18
GY	2019_Kincaid	103	EToin_04/06
GY	2019_Kincaid	103	EToin_04/13
GY	2019_Kincaid	103	EToin_05/26
GY	2019_Kincaid	103	EToin_06/21

GY	2019_Kincaid	103	EToin_06/22
GY	2019_Kincaid	103	EToin_06/25
GY	2019_Kincaid	103	EToin_06/26
GY	2019_Kincaid	103	EToin_06/27
GY	2019_Kincaid	103	EToin_07/02
GY	2019_Kincaid	103	EToin_07/08
GY	2019_Kincaid	103	ETrin_12/12
GY	2019_Kincaid	103	ETrin_01/16
GY	2019_Kincaid	103	ETrin_01/18
GY	2019_Kincaid	103	ETrin_01/19
GY	2019_Kincaid	103	ETrin_03/31
GY	2019_Kincaid	103	ETrin_04/06
GY	2019_Kincaid	103	ETrin_04/13
GY	2019_Kincaid	103	ETrin_05/24
GY	2019_Kincaid	103	ETrin_05/26
GY	2019_Kincaid	103	ETrin_06/21
GY	2019_Kincaid	103	ETrin_06/22
GY	2019_Kincaid	103	ETrin_06/25
GY	2019_Kincaid	103	ETrin_06/26
GY	2019_Kincaid	103	ETrin_06/27
GY	2019_Kincaid	103	ETrin_07/02
GY	2019_Kincaid	103	ETrin_07/08
GY	2019_Pullman	18	Max.F_11/09
GY	2019_Pullman	18	Avg1.5m.DP.F_02/16
GY	2019_Pullman	18	Avg1.5m.RH._04/02
GY	2019_Pullman	18	Avg1.5m.RH._04/23
GY	2019_Pullman	18	Avg1.5m.RH._06/01
GY	2019_Pullman	18	Avg1.5m.RH._06/02
GY	2019_Pullman	18	Avg1.5m.RH._06/20
GY	2019_Pullman	18	Avg1.5m.RH._06/23
GY	2019_Pullman	18	Avg1.5m.RH._06/24
GY	2019_Pullman	18	Avg1.5m.RH._06/29
GY	2019_Pullman	18	Avg1.5m.RH._06/30
GY	2019_Pullman	18	TotalSolarRadMJ.m._11/29
GY	2019_Pullman	18	TotalSolarRadMJ.m._04/27
GY	2019_Pullman	18	EToin_05/18
GY	2019_Pullman	18	EToin_07/01
GY	2019_Pullman	18	ETrin_01/29
GY	2019_Pullman	18	ETrin_05/18
GY	2019_Pullman	18	ETrin_06/01
GY	2020_Farmington	2	Avg1.5m.RH._06/29
GY	2020_Farmington	2	ETrin_07/08
GY	2020_Harrington	4	Avg1.5m.RH._06/29
GY	2020_Harrington	4	Avg1.5m.RH._06/30

GY	2020_Harrington	4	ETrin_03/31
GY	2020_Harrington	4	ETrin_07/08
GY	2020_Kincaid	233	Min.F_10/25
GY	2020_Kincaid	233	Min.F_10/26
GY	2020_Kincaid	233	Min.F_11/17
GY	2020_Kincaid	233	Min.F_11/22
GY	2020_Kincaid	233	Min.F_12/13
GY	2020_Kincaid	233	Min.F_01/18
GY	2020_Kincaid	233	Min.F_02/12
GY	2020_Kincaid	233	Min.F_03/23
GY	2020_Kincaid	233	Min.F_04/08
GY	2020_Kincaid	233	Min.F_04/09
GY	2020_Kincaid	233	Min.F_04/11
GY	2020_Kincaid	233	Min.F_04/26
GY	2020_Kincaid	233	Min.F_05/28
GY	2020_Kincaid	233	Min.F_06/06
GY	2020_Kincaid	233	Min.F_06/20
GY	2020_Kincaid	233	Min.F_06/27
GY	2020_Kincaid	233	Min.F_06/30
GY	2020_Kincaid	233	Avg.F_10/20
GY	2020_Kincaid	233	Avg.F_10/23
GY	2020_Kincaid	233	Avg.F_10/24
GY	2020_Kincaid	233	Avg.F_10/25
GY	2020_Kincaid	233	Avg.F_10/26
GY	2020_Kincaid	233	Avg.F_10/27
GY	2020_Kincaid	233	Avg.F_11/22
GY	2020_Kincaid	233	Avg.F_11/23
GY	2020_Kincaid	233	Avg.F_01/18
GY	2020_Kincaid	233	Avg.F_01/19
GY	2020_Kincaid	233	Avg.F_03/25
GY	2020_Kincaid	233	Avg.F_06/16
GY	2020_Kincaid	233	Avg.F_06/20
GY	2020_Kincaid	233	Max.F_10/17
GY	2020_Kincaid	233	Max.F_10/19
GY	2020_Kincaid	233	Max.F_10/21
GY	2020_Kincaid	233	Max.F_10/22
GY	2020_Kincaid	233	Max.F_10/23
GY	2020_Kincaid	233	Max.F_10/26
GY	2020_Kincaid	233	Max.F_10/27
GY	2020_Kincaid	233	Max.F_11/23
GY	2020_Kincaid	233	Max.F_12/14
GY	2020_Kincaid	233	Max.F_01/19
GY	2020_Kincaid	233	Max.F_04/25
GY	2020_Kincaid	233	Max.F_06/09

GY	2020_Kincaid	233	Max.F_06/25
GY	2020_Kincaid	233	Avg1.5m.DP.F_10/26
GY	2020_Kincaid	233	Avg1.5m.DP.F_10/27
GY	2020_Kincaid	233	Avg1.5m.DP.F_11/22
GY	2020_Kincaid	233	Avg1.5m.DP.F_01/03
GY	2020_Kincaid	233	Avg1.5m.DP.F_01/19
GY	2020_Kincaid	233	Avg1.5m.DP.F_02/12
GY	2020_Kincaid	233	Avg1.5m.DP.F_02/13
GY	2020_Kincaid	233	Avg1.5m.DP.F_03/12
GY	2020_Kincaid	233	Avg1.5m.DP.F_03/29
GY	2020_Kincaid	233	Avg1.5m.DP.F_03/30
GY	2020_Kincaid	233	Avg1.5m.DP.F_03/31
GY	2020_Kincaid	233	Avg1.5m.DP.F_04/08
GY	2020_Kincaid	233	Avg1.5m.DP.F_04/09
GY	2020_Kincaid	233	Avg1.5m.DP.F_04/11
GY	2020_Kincaid	233	Avg1.5m.DP.F_04/12
GY	2020_Kincaid	233	Avg1.5m.DP.F_04/13
GY	2020_Kincaid	233	Avg1.5m.DP.F_04/14
GY	2020_Kincaid	233	Avg1.5m.DP.F_04/16
GY	2020_Kincaid	233	Avg1.5m.DP.F_04/17
GY	2020_Kincaid	233	Avg1.5m.DP.F_04/18
GY	2020_Kincaid	233	Avg1.5m.DP.F_04/19
GY	2020_Kincaid	233	Avg1.5m.DP.F_04/20
GY	2020_Kincaid	233	Avg1.5m.DP.F_04/21
GY	2020_Kincaid	233	Avg1.5m.DP.F_04/23
GY	2020_Kincaid	233	Avg1.5m.DP.F_05/15
GY	2020_Kincaid	233	Avg1.5m.DP.F_05/16
GY	2020_Kincaid	233	Avg1.5m.DP.F_05/21
GY	2020_Kincaid	233	Avg1.5m.DP.F_05/26
GY	2020_Kincaid	233	Avg1.5m.DP.F_05/27
GY	2020_Kincaid	233	Avg1.5m.DP.F_05/28
GY	2020_Kincaid	233	Avg1.5m.DP.F_05/31
GY	2020_Kincaid	233	Avg1.5m.DP.F_06/01
GY	2020_Kincaid	233	Avg1.5m.DP.F_06/06
GY	2020_Kincaid	233	Avg1.5m.DP.F_06/16
GY	2020_Kincaid	233	Avg1.5m.DP.F_06/17
GY	2020_Kincaid	233	Avg1.5m.DP.F_07/05
GY	2020_Kincaid	233	Avg1.5m.RH._10/18
GY	2020_Kincaid	233	Avg1.5m.RH._10/30
GY	2020_Kincaid	233	Avg1.5m.RH._10/31
GY	2020_Kincaid	233	Avg1.5m.RH._11/01
GY	2020_Kincaid	233	Avg1.5m.RH._11/14
GY	2020_Kincaid	233	Avg1.5m.RH._11/26
GY	2020_Kincaid	233	Avg1.5m.RH._12/15

GY	2020_Kincaid	233	Avg1.5m.RH._01/01
GY	2020_Kincaid	233	Avg1.5m.RH._01/02
GY	2020_Kincaid	233	Avg1.5m.RH._01/09
GY	2020_Kincaid	233	Avg1.5m.RH._01/16
GY	2020_Kincaid	233	Avg1.5m.RH._03/18
GY	2020_Kincaid	233	Avg1.5m.RH._03/30
GY	2020_Kincaid	233	Avg1.5m.RH._04/09
GY	2020_Kincaid	233	Avg1.5m.RH._04/11
GY	2020_Kincaid	233	Avg1.5m.RH._04/12
GY	2020_Kincaid	233	Avg1.5m.RH._04/13
GY	2020_Kincaid	233	Avg1.5m.RH._04/16
GY	2020_Kincaid	233	Avg1.5m.RH._04/17
GY	2020_Kincaid	233	Avg1.5m.RH._04/18
GY	2020_Kincaid	233	Avg1.5m.RH._04/20
GY	2020_Kincaid	233	Avg1.5m.RH._04/25
GY	2020_Kincaid	233	Avg1.5m.RH._05/15
GY	2020_Kincaid	233	Avg1.5m.RH._05/16
GY	2020_Kincaid	233	Avg1.5m.RH._05/26
GY	2020_Kincaid	233	Avg1.5m.RH._06/08
GY	2020_Kincaid	233	Avg1.5m.RH._06/21
GY	2020_Kincaid	233	Avg1.5m.RH._06/22
GY	2020_Kincaid	233	Avg1.5m.RH._06/23
GY	2020_Kincaid	233	Avg1.5m.RH._06/26
GY	2020_Kincaid	233	Avg1.5m.RH._06/27
GY	2020_Kincaid	233	Avg1.5m.RH._06/29
GY	2020_Kincaid	233	Avg1.5m.RH._06/30
GY	2020_Kincaid	233	Avg1.5m.RH._07/02
GY	2020_Kincaid	233	Avg1.5m.RH._07/08
GY	2020_Kincaid	233	Min.F.1_10/24
GY	2020_Kincaid	233	Min.F.1_10/25
GY	2020_Kincaid	233	Min.F.1_10/26
GY	2020_Kincaid	233	Min.F.1_10/27
GY	2020_Kincaid	233	Min.F.1_10/28
GY	2020_Kincaid	233	Min.F.1_10/29
GY	2020_Kincaid	233	Min.F.1_11/23
GY	2020_Kincaid	233	Min.F.1_11/24
GY	2020_Kincaid	233	Min.F.1_06/27
GY	2020_Kincaid	233	Min.F.1_06/28
GY	2020_Kincaid	233	Min.F.1_06/29
GY	2020_Kincaid	233	Min.F.1_06/30
GY	2020_Kincaid	233	Avg.F.1_10/24
GY	2020_Kincaid	233	Avg.F.1_10/25
GY	2020_Kincaid	233	Avg.F.1_10/26
GY	2020_Kincaid	233	Avg.F.1_10/27

GY	2020_Kincaid	233	Avg.F.1_10/28
GY	2020_Kincaid	233	Avg.F.1_10/29
GY	2020_Kincaid	233	Avg.F.1_11/23
GY	2020_Kincaid	233	Avg.F.1_11/24
GY	2020_Kincaid	233	Avg.F.1_06/27
GY	2020_Kincaid	233	Avg.F.1_06/28
GY	2020_Kincaid	233	Avg.F.1_06/29
GY	2020_Kincaid	233	Avg.F.1_06/30
GY	2020_Kincaid	233	TotPrecin_11/04
GY	2020_Kincaid	233	TotPrecin_12/23
GY	2020_Kincaid	233	TotPrecin_01/12
GY	2020_Kincaid	233	TotPrecin_02/17
GY	2020_Kincaid	233	TotPrecin_04/11
GY	2020_Kincaid	233	TotPrecin_04/19
GY	2020_Kincaid	233	TotPrecin_05/26
GY	2020_Kincaid	233	TotPrecin_06/09
GY	2020_Kincaid	233	TotalSolarRadMJ.m._10/25
GY	2020_Kincaid	233	TotalSolarRadMJ.m._10/26
GY	2020_Kincaid	233	TotalSolarRadMJ.m._10/28
GY	2020_Kincaid	233	TotalSolarRadMJ.m._11/16
GY	2020_Kincaid	233	TotalSolarRadMJ.m._11/17
GY	2020_Kincaid	233	TotalSolarRadMJ.m._11/21
GY	2020_Kincaid	233	TotalSolarRadMJ.m._12/02
GY	2020_Kincaid	233	TotalSolarRadMJ.m._12/08
GY	2020_Kincaid	233	TotalSolarRadMJ.m._01/02
GY	2020_Kincaid	233	TotalSolarRadMJ.m._01/20
GY	2020_Kincaid	233	TotalSolarRadMJ.m._01/29
GY	2020_Kincaid	233	TotalSolarRadMJ.m._03/05
GY	2020_Kincaid	233	TotalSolarRadMJ.m._03/09
GY	2020_Kincaid	233	TotalSolarRadMJ.m._03/20
GY	2020_Kincaid	233	TotalSolarRadMJ.m._04/08
GY	2020_Kincaid	233	TotalSolarRadMJ.m._04/17
GY	2020_Kincaid	233	TotalSolarRadMJ.m._04/24
GY	2020_Kincaid	233	TotalSolarRadMJ.m._04/28
GY	2020_Kincaid	233	TotalSolarRadMJ.m._05/09
GY	2020_Kincaid	233	TotalSolarRadMJ.m._05/10
GY	2020_Kincaid	233	TotalSolarRadMJ.m._06/15
GY	2020_Kincaid	233	TotalSolarRadMJ.m._07/02
GY	2020_Kincaid	233	EToin_10/23
GY	2020_Kincaid	233	EToin_10/30
GY	2020_Kincaid	233	EToin_11/01
GY	2020_Kincaid	233	EToin_11/14
GY	2020_Kincaid	233	EToin_12/12
GY	2020_Kincaid	233	EToin_12/15

GY	2020_Kincaid	233	EToin_01/03
GY	2020_Kincaid	233	EToin_01/18
GY	2020_Kincaid	233	EToin_03/04
GY	2020_Kincaid	233	EToin_03/18
GY	2020_Kincaid	233	EToin_04/12
GY	2020_Kincaid	233	EToin_04/13
GY	2020_Kincaid	233	EToin_04/15
GY	2020_Kincaid	233	EToin_04/17
GY	2020_Kincaid	233	EToin_04/20
GY	2020_Kincaid	233	EToin_04/24
GY	2020_Kincaid	233	EToin_04/25
GY	2020_Kincaid	233	EToin_04/29
GY	2020_Kincaid	233	EToin_05/16
GY	2020_Kincaid	233	EToin_06/08
GY	2020_Kincaid	233	EToin_06/21
GY	2020_Kincaid	233	EToin_06/22
GY	2020_Kincaid	233	EToin_06/25
GY	2020_Kincaid	233	EToin_06/26
GY	2020_Kincaid	233	EToin_06/27
GY	2020_Kincaid	233	EToin_06/29
GY	2020_Kincaid	233	EToin_07/02
GY	2020_Kincaid	233	EToin_07/08
GY	2020_Kincaid	233	ETrin_11/01
GY	2020_Kincaid	233	ETrin_11/14
GY	2020_Kincaid	233	ETrin_12/12
GY	2020_Kincaid	233	ETrin_01/07
GY	2020_Kincaid	233	ETrin_01/09
GY	2020_Kincaid	233	ETrin_01/23
GY	2020_Kincaid	233	ETrin_01/24
GY	2020_Kincaid	233	ETrin_03/04
GY	2020_Kincaid	233	ETrin_03/13
GY	2020_Kincaid	233	ETrin_03/18
GY	2020_Kincaid	233	ETrin_03/31
GY	2020_Kincaid	233	ETrin_04/12
GY	2020_Kincaid	233	ETrin_04/13
GY	2020_Kincaid	233	ETrin_04/15
GY	2020_Kincaid	233	ETrin_04/16
GY	2020_Kincaid	233	ETrin_04/17
GY	2020_Kincaid	233	ETrin_04/18
GY	2020_Kincaid	233	ETrin_04/20
GY	2020_Kincaid	233	ETrin_04/24
GY	2020_Kincaid	233	ETrin_04/29
GY	2020_Kincaid	233	ETrin_05/15
GY	2020_Kincaid	233	ETrin_05/16

GY	2020_Kincaid	233	ETrin_05/24
GY	2020_Kincaid	233	ETrin_05/26
GY	2020_Kincaid	233	ETrin_05/27
GY	2020_Kincaid	233	ETrin_06/08
GY	2020_Kincaid	233	ETrin_06/21
GY	2020_Kincaid	233	ETrin_06/22
GY	2020_Kincaid	233	ETrin_06/24
GY	2020_Kincaid	233	ETrin_06/25
GY	2020_Kincaid	233	ETrin_06/26
GY	2020_Kincaid	233	ETrin_06/27
GY	2020_Kincaid	233	ETrin_06/29
GY	2020_Kincaid	233	ETrin_07/02
GY	2020_Kincaid	233	ETrin_07/03
GY	2020_Kincaid	233	ETrin_07/05
GY	2020_Kincaid	233	ETrin_07/08
GY	2020_Ritzville	1	Avg1.5m.RH._06/29
GY	2020_Walla_Walla	6	Max.F_12/12
GY	2020_Walla_Walla	6	Avg1.5m.DP.F_05/26
GY	2020_Walla_Walla	6	Avg1.5m.RH._06/29
GY	2020_Walla_Walla	6	Avg1.5m.RH._07/02
GY	2020_Walla_Walla	6	TotalSolarRadMJ.m._12/08
GY	2020_Walla_Walla	6	ETrin_07/08
GY	2021_Davenport	103	Min.F_10/25
GY	2021_Davenport	103	Min.F_12/12
GY	2021_Davenport	103	Min.F_12/13
GY	2021_Davenport	103	Min.F_12/23
GY	2021_Davenport	103	Min.F_02/13
GY	2021_Davenport	103	Min.F_04/26
GY	2021_Davenport	103	Min.F_05/09
GY	2021_Davenport	103	Min.F_06/06
GY	2021_Davenport	103	Avg.F_10/25
GY	2021_Davenport	103	Avg.F_12/12
GY	2021_Davenport	103	Avg.F_06/29
GY	2021_Davenport	103	Avg.F_06/30
GY	2021_Davenport	103	Avg.F_07/02
GY	2021_Davenport	103	Max.F_10/25
GY	2021_Davenport	103	Max.F_12/12
GY	2021_Davenport	103	Max.F_04/25
GY	2021_Davenport	103	Max.F_06/29
GY	2021_Davenport	103	Max.F_06/30
GY	2021_Davenport	103	Max.F_07/08
GY	2021_Davenport	103	Avg1.5m.DP.F_02/12
GY	2021_Davenport	103	Avg1.5m.DP.F_02/13
GY	2021_Davenport	103	Avg1.5m.DP.F_03/29

GY	2021_Davenport	103	Avg1.5m.DP.F_03/30
GY	2021_Davenport	103	Avg1.5m.DP.F_05/26
GY	2021_Davenport	103	Avg1.5m.DP.F_05/28
GY	2021_Davenport	103	Avg1.5m.DP.F_05/29
GY	2021_Davenport	103	Avg1.5m.DP.F_06/06
GY	2021_Davenport	103	Avg1.5m.RH._03/30
GY	2021_Davenport	103	Avg1.5m.RH._03/31
GY	2021_Davenport	103	Avg1.5m.RH._04/01
GY	2021_Davenport	103	Avg1.5m.RH._04/23
GY	2021_Davenport	103	Avg1.5m.RH._05/15
GY	2021_Davenport	103	Avg1.5m.RH._05/26
GY	2021_Davenport	103	Avg1.5m.RH._06/01
GY	2021_Davenport	103	Avg1.5m.RH._06/08
GY	2021_Davenport	103	Avg1.5m.RH._06/21
GY	2021_Davenport	103	Avg1.5m.RH._06/22
GY	2021_Davenport	103	Avg1.5m.RH._06/23
GY	2021_Davenport	103	Avg1.5m.RH._06/24
GY	2021_Davenport	103	Avg1.5m.RH._06/25
GY	2021_Davenport	103	Avg1.5m.RH._06/26
GY	2021_Davenport	103	Avg1.5m.RH._06/27
GY	2021_Davenport	103	Avg1.5m.RH._06/28
GY	2021_Davenport	103	Avg1.5m.RH._06/29
GY	2021_Davenport	103	Avg1.5m.RH._06/30
GY	2021_Davenport	103	Avg1.5m.RH._07/02
GY	2021_Davenport	103	Avg1.5m.RH._07/08
GY	2021_Davenport	103	Min.F.1_06/29
GY	2021_Davenport	103	Min.F.1_06/30
GY	2021_Davenport	103	Min.F.1_07/01
GY	2021_Davenport	103	Min.F.1_07/02
GY	2021_Davenport	103	Min.F.1_07/03
GY	2021_Davenport	103	Min.F.1_07/04
GY	2021_Davenport	103	Min.F.1_07/05
GY	2021_Davenport	103	Min.F.1_07/06
GY	2021_Davenport	103	Min.F.1_07/07
GY	2021_Davenport	103	Avg.F.1_06/29
GY	2021_Davenport	103	Avg.F.1_06/30
GY	2021_Davenport	103	Avg.F.1_07/01
GY	2021_Davenport	103	Avg.F.1_07/02
GY	2021_Davenport	103	Avg.F.1_07/03
GY	2021_Davenport	103	Avg.F.1_07/04
GY	2021_Davenport	103	Avg.F.1_07/05
GY	2021_Davenport	103	Avg.F.1_07/06
GY	2021_Davenport	103	Avg.F.1_07/07
GY	2021_Davenport	103	TotPrecin_12/15

GY	2021_Davenport	103	TotPrecin_02/17
GY	2021_Davenport	103	TotalSolarRadMJ.m._10/28
GY	2021_Davenport	103	TotalSolarRadMJ.m._12/08
GY	2021_Davenport	103	TotalSolarRadMJ.m._12/23
GY	2021_Davenport	103	TotalSolarRadMJ.m._04/27
GY	2021_Davenport	103	TotalSolarRadMJ.m._05/09
GY	2021_Davenport	103	ETоin_11/01
GY	2021_Davenport	103	ETоin_12/12
GY	2021_Davenport	103	ETоin_01/07
GY	2021_Davenport	103	ETоin_03/18
GY	2021_Davenport	103	ETоin_03/31
GY	2021_Davenport	103	ETоin_04/18
GY	2021_Davenport	103	ETоin_05/26
GY	2021_Davenport	103	ETоin_06/08
GY	2021_Davenport	103	ETоin_06/24
GY	2021_Davenport	103	ETоin_06/25
GY	2021_Davenport	103	ETоin_06/28
GY	2021_Davenport	103	ETоin_06/29
GY	2021_Davenport	103	ETоin_07/02
GY	2021_Davenport	103	ETоin_07/05
GY	2021_Davenport	103	ETоin_07/08
GY	2021_Davenport	103	ETrin_12/12
GY	2021_Davenport	103	ETrin_01/07
GY	2021_Davenport	103	ETrin_03/18
GY	2021_Davenport	103	ETrin_03/31
GY	2021_Davenport	103	ETrin_04/01
GY	2021_Davenport	103	ETrin_04/18
GY	2021_Davenport	103	ETrin_05/26
GY	2021_Davenport	103	ETrin_06/22
GY	2021_Davenport	103	ETrin_06/24
GY	2021_Davenport	103	ETrin_06/25
GY	2021_Davenport	103	ETrin_06/28
GY	2021_Davenport	103	ETrin_06/29
GY	2021_Davenport	103	ETrin_07/01
GY	2021_Davenport	103	ETrin_07/02
GY	2021_Davenport	103	ETrin_07/05
GY	2021_Davenport	103	ETrin_07/08
GY	2021_Harrington	138	Min.F_10/25
GY	2021_Harrington	138	Min.F_12/12
GY	2021_Harrington	138	Min.F_12/13
GY	2021_Harrington	138	Min.F_12/23
GY	2021_Harrington	138	Min.F_02/13
GY	2021_Harrington	138	Min.F_03/23
GY	2021_Harrington	138	Min.F_04/06

GY	2021_Harrington	138	Min.F_04/26
GY	2021_Harrington	138	Min.F_05/09
GY	2021_Harrington	138	Min.F_06/06
GY	2021_Harrington	138	Avg.F_10/25
GY	2021_Harrington	138	Avg.F_12/12
GY	2021_Harrington	138	Avg.F_12/13
GY	2021_Harrington	138	Avg.F_06/28
GY	2021_Harrington	138	Avg.F_06/29
GY	2021_Harrington	138	Avg.F_06/30
GY	2021_Harrington	138	Avg.F_07/01
GY	2021_Harrington	138	Avg.F_07/02
GY	2021_Harrington	138	Avg.F_07/06
GY	2021_Harrington	138	Avg.F_07/08
GY	2021_Harrington	138	Max.F_10/25
GY	2021_Harrington	138	Max.F_12/12
GY	2021_Harrington	138	Max.F_04/25
GY	2021_Harrington	138	Max.F_06/28
GY	2021_Harrington	138	Max.F_06/29
GY	2021_Harrington	138	Max.F_06/30
GY	2021_Harrington	138	Max.F_07/07
GY	2021_Harrington	138	Max.F_07/08
GY	2021_Harrington	138	Avg1.5m.DP.F_10/25
GY	2021_Harrington	138	Avg1.5m.DP.F_12/03
GY	2021_Harrington	138	Avg1.5m.DP.F_12/23
GY	2021_Harrington	138	Avg1.5m.DP.F_12/24
GY	2021_Harrington	138	Avg1.5m.DP.F_02/12
GY	2021_Harrington	138	Avg1.5m.DP.F_02/13
GY	2021_Harrington	138	Avg1.5m.DP.F_02/14
GY	2021_Harrington	138	Avg1.5m.DP.F_03/29
GY	2021_Harrington	138	Avg1.5m.DP.F_03/30
GY	2021_Harrington	138	Avg1.5m.DP.F_04/06
GY	2021_Harrington	138	Avg1.5m.DP.F_04/23
GY	2021_Harrington	138	Avg1.5m.DP.F_05/18
GY	2021_Harrington	138	Avg1.5m.DP.F_05/26
GY	2021_Harrington	138	Avg1.5m.DP.F_05/28
GY	2021_Harrington	138	Avg1.5m.DP.F_05/29
GY	2021_Harrington	138	Avg1.5m.DP.F_06/06
GY	2021_Harrington	138	Avg1.5m.RH._03/30
GY	2021_Harrington	138	Avg1.5m.RH._03/31
GY	2021_Harrington	138	Avg1.5m.RH._04/01
GY	2021_Harrington	138	Avg1.5m.RH._04/06
GY	2021_Harrington	138	Avg1.5m.RH._04/23
GY	2021_Harrington	138	Avg1.5m.RH._05/15
GY	2021_Harrington	138	Avg1.5m.RH._05/26

GY	2021_Harrington	138	Avg1.5m.RH._06/01
GY	2021_Harrington	138	Avg1.5m.RH._06/08
GY	2021_Harrington	138	Avg1.5m.RH._06/21
GY	2021_Harrington	138	Avg1.5m.RH._06/22
GY	2021_Harrington	138	Avg1.5m.RH._06/23
GY	2021_Harrington	138	Avg1.5m.RH._06/24
GY	2021_Harrington	138	Avg1.5m.RH._06/25
GY	2021_Harrington	138	Avg1.5m.RH._06/26
GY	2021_Harrington	138	Avg1.5m.RH._06/27
GY	2021_Harrington	138	Avg1.5m.RH._06/28
GY	2021_Harrington	138	Avg1.5m.RH._06/29
GY	2021_Harrington	138	Avg1.5m.RH._06/30
GY	2021_Harrington	138	Avg1.5m.RH._07/02
GY	2021_Harrington	138	Avg1.5m.RH._07/05
GY	2021_Harrington	138	Avg1.5m.RH._07/08
GY	2021_Harrington	138	Min.F.1_06/28
GY	2021_Harrington	138	Min.F.1_06/29
GY	2021_Harrington	138	Min.F.1_06/30
GY	2021_Harrington	138	Min.F.1_07/01
GY	2021_Harrington	138	Min.F.1_07/02
GY	2021_Harrington	138	Min.F.1_07/03
GY	2021_Harrington	138	Min.F.1_07/04
GY	2021_Harrington	138	Min.F.1_07/05
GY	2021_Harrington	138	Min.F.1_07/06
GY	2021_Harrington	138	Min.F.1_07/07
GY	2021_Harrington	138	Min.F.1_07/08
GY	2021_Harrington	138	Avg.F.1_06/28
GY	2021_Harrington	138	Avg.F.1_06/29
GY	2021_Harrington	138	Avg.F.1_06/30
GY	2021_Harrington	138	Avg.F.1_07/01
GY	2021_Harrington	138	Avg.F.1_07/02
GY	2021_Harrington	138	Avg.F.1_07/03
GY	2021_Harrington	138	Avg.F.1_07/04
GY	2021_Harrington	138	Avg.F.1_07/05
GY	2021_Harrington	138	Avg.F.1_07/06
GY	2021_Harrington	138	Avg.F.1_07/07
GY	2021_Harrington	138	Avg.F.1_07/08
GY	2021_Harrington	138	TotPrecin_11/13
GY	2021_Harrington	138	TotPrecin_11/18
GY	2021_Harrington	138	TotPrecin_02/17
GY	2021_Harrington	138	TotPrecin_03/29
GY	2021_Harrington	138	TotalSolarRadMJ.m._10/28
GY	2021_Harrington	138	TotalSolarRadMJ.m._12/08
GY	2021_Harrington	138	TotalSolarRadMJ.m._12/23

GY	2021_Harrington	138	TotalSolarRadMJ.m._03/27
GY	2021_Harrington	138	TotalSolarRadMJ.m._04/15
GY	2021_Harrington	138	TotalSolarRadMJ.m._04/27
GY	2021_Harrington	138	TotalSolarRadMJ.m._05/09
GY	2021_Harrington	138	TotalSolarRadMJ.m._05/10
GY	2021_Harrington	138	TotalSolarRadMJ.m._05/26
GY	2021_Harrington	138	ETоin_11/01
GY	2021_Harrington	138	ETоin_12/12
GY	2021_Harrington	138	ETоin_01/07
GY	2021_Harrington	138	ETоin_03/14
GY	2021_Harrington	138	ETоin_03/18
GY	2021_Harrington	138	ETоin_03/31
GY	2021_Harrington	138	ETоin_04/15
GY	2021_Harrington	138	ETоin_04/18
GY	2021_Harrington	138	ETоin_05/26
GY	2021_Harrington	138	ETоin_06/08
GY	2021_Harrington	138	ETоin_06/24
GY	2021_Harrington	138	ETоin_06/25
GY	2021_Harrington	138	ETоin_06/28
GY	2021_Harrington	138	ETоin_06/29
GY	2021_Harrington	138	ETоin_06/30
GY	2021_Harrington	138	ETоin_07/02
GY	2021_Harrington	138	ETоin_07/05
GY	2021_Harrington	138	ETоin_07/08
GY	2021_Harrington	138	ETrin_12/12
GY	2021_Harrington	138	ETrin_01/07
GY	2021_Harrington	138	ETrin_03/14
GY	2021_Harrington	138	ETrin_03/18
GY	2021_Harrington	138	ETrin_03/31
GY	2021_Harrington	138	ETrin_04/01
GY	2021_Harrington	138	ETrin_04/15
GY	2021_Harrington	138	ETrin_04/18
GY	2021_Harrington	138	ETrin_05/18
GY	2021_Harrington	138	ETrin_05/26
GY	2021_Harrington	138	ETrin_06/22
GY	2021_Harrington	138	ETrin_06/24
GY	2021_Harrington	138	ETrin_06/25
GY	2021_Harrington	138	ETrin_06/28
GY	2021_Harrington	138	ETrin_06/29
GY	2021_Harrington	138	ETrin_07/01
GY	2021_Harrington	138	ETrin_07/02
GY	2021_Harrington	138	ETrin_07/05
GY	2021_Harrington	138	ETrin_07/08
GY	2021_Kahlotus	1	TotalSolarRadMJ.m._12/08

GY	2021_Kincaid	4	Avg1.5m.RH._06/29
GY	2021_Kincaid	4	TotalSolarRadMJ.m._12/08
GY	2021_Kincaid	4	EToin_07/08
GY	2021_Kincaid	4	ETrin_07/08
GY	2021_Pullman	25	Avg1.5m.DP.F_05/26
GY	2021_Pullman	25	Avg1.5m.RH._12/18
GY	2021_Pullman	25	Avg1.5m.RH._01/19
GY	2021_Pullman	25	Avg1.5m.RH._05/26
GY	2021_Pullman	25	Avg1.5m.RH._06/01
GY	2021_Pullman	25	Avg1.5m.RH._06/22
GY	2021_Pullman	25	Avg1.5m.RH._06/23
GY	2021_Pullman	25	Avg1.5m.RH._06/24
GY	2021_Pullman	25	Avg1.5m.RH._06/25
GY	2021_Pullman	25	Avg1.5m.RH._06/26
GY	2021_Pullman	25	Avg1.5m.RH._06/29
GY	2021_Pullman	25	Avg1.5m.RH._07/02
GY	2021_Pullman	25	EToin_12/31
GY	2021_Pullman	25	EToin_06/28
GY	2021_Pullman	25	EToin_06/29
GY	2021_Pullman	25	EToin_07/02
GY	2021_Pullman	25	ETrin_12/31
GY	2021_Pullman	25	ETrin_05/26
GY	2021_Pullman	25	ETrin_06/23
GY	2021_Pullman	25	ETrin_06/26
GY	2021_Pullman	25	ETrin_06/28
GY	2021_Pullman	25	ETrin_06/29
GY	2021_Pullman	25	ETrin_07/01
GY	2021_Pullman	25	ETrin_07/02
GY	2021_Pullman	25	ETrin_07/08
GY	2021_Ritzville	174	Min.F_10/25
GY	2021_Ritzville	174	Min.F_12/12
GY	2021_Ritzville	174	Min.F_12/13
GY	2021_Ritzville	174	Min.F_12/23
GY	2021_Ritzville	174	Min.F_01/25
GY	2021_Ritzville	174	Min.F_02/12
GY	2021_Ritzville	174	Min.F_02/13
GY	2021_Ritzville	174	Min.F_03/23
GY	2021_Ritzville	174	Min.F_04/06
GY	2021_Ritzville	174	Min.F_04/26
GY	2021_Ritzville	174	Min.F_05/09
GY	2021_Ritzville	174	Min.F_06/06
GY	2021_Ritzville	174	Min.F_07/01
GY	2021_Ritzville	174	Avg.F_10/25
GY	2021_Ritzville	174	Avg.F_12/12

GY	2021_Ritzville	174	Avg.F_12/13
GY	2021_Ritzville	174	Avg.F_02/12
GY	2021_Ritzville	174	Avg.F_06/28
GY	2021_Ritzville	174	Avg.F_06/29
GY	2021_Ritzville	174	Avg.F_06/30
GY	2021_Ritzville	174	Avg.F_07/01
GY	2021_Ritzville	174	Avg.F_07/02
GY	2021_Ritzville	174	Avg.F_07/03
GY	2021_Ritzville	174	Avg.F_07/06
GY	2021_Ritzville	174	Avg.F_07/08
GY	2021_Ritzville	174	Max.F_10/25
GY	2021_Ritzville	174	Max.F_12/12
GY	2021_Ritzville	174	Max.F_04/25
GY	2021_Ritzville	174	Max.F_06/28
GY	2021_Ritzville	174	Max.F_06/29
GY	2021_Ritzville	174	Max.F_06/30
GY	2021_Ritzville	174	Max.F_07/02
GY	2021_Ritzville	174	Max.F_07/07
GY	2021_Ritzville	174	Max.F_07/08
GY	2021_Ritzville	174	Avg1.5m.DP.F_10/25
GY	2021_Ritzville	174	Avg1.5m.DP.F_12/03
GY	2021_Ritzville	174	Avg1.5m.DP.F_12/23
GY	2021_Ritzville	174	Avg1.5m.DP.F_12/24
GY	2021_Ritzville	174	Avg1.5m.DP.F_02/12
GY	2021_Ritzville	174	Avg1.5m.DP.F_02/13
GY	2021_Ritzville	174	Avg1.5m.DP.F_02/14
GY	2021_Ritzville	174	Avg1.5m.DP.F_03/29
GY	2021_Ritzville	174	Avg1.5m.DP.F_03/30
GY	2021_Ritzville	174	Avg1.5m.DP.F_04/06
GY	2021_Ritzville	174	Avg1.5m.DP.F_04/10
GY	2021_Ritzville	174	Avg1.5m.DP.F_04/21
GY	2021_Ritzville	174	Avg1.5m.DP.F_04/23
GY	2021_Ritzville	174	Avg1.5m.DP.F_05/16
GY	2021_Ritzville	174	Avg1.5m.DP.F_05/18
GY	2021_Ritzville	174	Avg1.5m.DP.F_05/26
GY	2021_Ritzville	174	Avg1.5m.DP.F_05/28
GY	2021_Ritzville	174	Avg1.5m.DP.F_05/29
GY	2021_Ritzville	174	Avg1.5m.DP.F_06/06
GY	2021_Ritzville	174	Avg1.5m.RH._12/06
GY	2021_Ritzville	174	Avg1.5m.RH._01/19
GY	2021_Ritzville	174	Avg1.5m.RH._03/18
GY	2021_Ritzville	174	Avg1.5m.RH._03/29
GY	2021_Ritzville	174	Avg1.5m.RH._03/30
GY	2021_Ritzville	174	Avg1.5m.RH._03/31

GY	2021_Ritzville	174	Avg1.5m.RH._04/01
GY	2021_Ritzville	174	Avg1.5m.RH._04/06
GY	2021_Ritzville	174	Avg1.5m.RH._04/19
GY	2021_Ritzville	174	Avg1.5m.RH._04/23
GY	2021_Ritzville	174	Avg1.5m.RH._05/15
GY	2021_Ritzville	174	Avg1.5m.RH._05/16
GY	2021_Ritzville	174	Avg1.5m.RH._05/26
GY	2021_Ritzville	174	Avg1.5m.RH._06/01
GY	2021_Ritzville	174	Avg1.5m.RH._06/02
GY	2021_Ritzville	174	Avg1.5m.RH._06/08
GY	2021_Ritzville	174	Avg1.5m.RH._06/21
GY	2021_Ritzville	174	Avg1.5m.RH._06/22
GY	2021_Ritzville	174	Avg1.5m.RH._06/23
GY	2021_Ritzville	174	Avg1.5m.RH._06/24
GY	2021_Ritzville	174	Avg1.5m.RH._06/25
GY	2021_Ritzville	174	Avg1.5m.RH._06/26
GY	2021_Ritzville	174	Avg1.5m.RH._06/27
GY	2021_Ritzville	174	Avg1.5m.RH._06/28
GY	2021_Ritzville	174	Avg1.5m.RH._06/29
GY	2021_Ritzville	174	Avg1.5m.RH._06/30
GY	2021_Ritzville	174	Avg1.5m.RH._07/02
GY	2021_Ritzville	174	Avg1.5m.RH._07/05
GY	2021_Ritzville	174	Avg1.5m.RH._07/08
GY	2021_Ritzville	174	Min.F.1_06/28
GY	2021_Ritzville	174	Min.F.1_06/29
GY	2021_Ritzville	174	Min.F.1_06/30
GY	2021_Ritzville	174	Min.F.1_07/01
GY	2021_Ritzville	174	Min.F.1_07/02
GY	2021_Ritzville	174	Min.F.1_07/03
GY	2021_Ritzville	174	Min.F.1_07/04
GY	2021_Ritzville	174	Min.F.1_07/05
GY	2021_Ritzville	174	Min.F.1_07/06
GY	2021_Ritzville	174	Min.F.1_07/07
GY	2021_Ritzville	174	Min.F.1_07/08
GY	2021_Ritzville	174	Avg.F.1_06/28
GY	2021_Ritzville	174	Avg.F.1_06/29
GY	2021_Ritzville	174	Avg.F.1_06/30
GY	2021_Ritzville	174	Avg.F.1_07/01
GY	2021_Ritzville	174	Avg.F.1_07/02
GY	2021_Ritzville	174	Avg.F.1_07/03
GY	2021_Ritzville	174	Avg.F.1_07/04
GY	2021_Ritzville	174	Avg.F.1_07/05
GY	2021_Ritzville	174	Avg.F.1_07/06
GY	2021_Ritzville	174	Avg.F.1_07/07

GY	2021_Ritzville	174	Avg.F.1_07/08
GY	2021_Ritzville	174	TotPrecin_11/13
GY	2021_Ritzville	174	TotPrecin_11/18
GY	2021_Ritzville	174	TotPrecin_12/15
GY	2021_Ritzville	174	TotPrecin_02/17
GY	2021_Ritzville	174	TotPrecin_03/29
GY	2021_Ritzville	174	TotalSolarRadMJ.m._10/23
GY	2021_Ritzville	174	TotalSolarRadMJ.m._10/28
GY	2021_Ritzville	174	TotalSolarRadMJ.m._12/08
GY	2021_Ritzville	174	TotalSolarRadMJ.m._12/23
GY	2021_Ritzville	174	TotalSolarRadMJ.m._03/27
GY	2021_Ritzville	174	TotalSolarRadMJ.m._04/15
GY	2021_Ritzville	174	TotalSolarRadMJ.m._04/27
GY	2021_Ritzville	174	TotalSolarRadMJ.m._05/09
GY	2021_Ritzville	174	TotalSolarRadMJ.m._05/10
GY	2021_Ritzville	174	TotalSolarRadMJ.m._05/26
GY	2021_Ritzville	174	EToin_10/22
GY	2021_Ritzville	174	EToin_11/01
GY	2021_Ritzville	174	EToin_12/12
GY	2021_Ritzville	174	EToin_01/07
GY	2021_Ritzville	174	EToin_03/14
GY	2021_Ritzville	174	EToin_03/18
GY	2021_Ritzville	174	EToin_03/31
GY	2021_Ritzville	174	EToin_04/15
GY	2021_Ritzville	174	EToin_04/18
GY	2021_Ritzville	174	EToin_05/16
GY	2021_Ritzville	174	EToin_05/18
GY	2021_Ritzville	174	EToin_05/26
GY	2021_Ritzville	174	EToin_06/08
GY	2021_Ritzville	174	EToin_06/20
GY	2021_Ritzville	174	EToin_06/22
GY	2021_Ritzville	174	EToin_06/24
GY	2021_Ritzville	174	EToin_06/25
GY	2021_Ritzville	174	EToin_06/27
GY	2021_Ritzville	174	EToin_06/28
GY	2021_Ritzville	174	EToin_06/29
GY	2021_Ritzville	174	EToin_06/30
GY	2021_Ritzville	174	EToin_07/01
GY	2021_Ritzville	174	EToin_07/02
GY	2021_Ritzville	174	EToin_07/05
GY	2021_Ritzville	174	EToin_07/08
GY	2021_Ritzville	174	ETrin_11/01
GY	2021_Ritzville	174	ETrin_12/12
GY	2021_Ritzville	174	ETrin_01/07

GY	2021_Ritzville	174	ETrin_01/29
GY	2021_Ritzville	174	ETrin_03/14
GY	2021_Ritzville	174	ETrin_03/18
GY	2021_Ritzville	174	ETrin_03/31
GY	2021_Ritzville	174	ETrin_04/01
GY	2021_Ritzville	174	ETrin_04/13
GY	2021_Ritzville	174	ETrin_04/15
GY	2021_Ritzville	174	ETrin_04/18
GY	2021_Ritzville	174	ETrin_05/15
GY	2021_Ritzville	174	ETrin_05/16
GY	2021_Ritzville	174	ETrin_05/18
GY	2021_Ritzville	174	ETrin_05/26
GY	2021_Ritzville	174	ETrin_06/02
GY	2021_Ritzville	174	ETrin_06/21
GY	2021_Ritzville	174	ETrin_06/22
GY	2021_Ritzville	174	ETrin_06/24
GY	2021_Ritzville	174	ETrin_06/25
GY	2021_Ritzville	174	ETrin_06/26
GY	2021_Ritzville	174	ETrin_06/27
GY	2021_Ritzville	174	ETrin_06/28
GY	2021_Ritzville	174	ETrin_06/29
GY	2021_Ritzville	174	ETrin_06/30
GY	2021_Ritzville	174	ETrin_07/01
GY	2021_Ritzville	174	ETrin_07/02
GY	2021_Ritzville	174	ETrin_07/03
GY	2021_Ritzville	174	ETrin_07/05
GY	2021_Ritzville	174	ETrin_07/08
GY	2021_Walla_Walla	40	Min.F_12/13
GY	2021_Walla_Walla	40	Avg.F_06/29
GY	2021_Walla_Walla	40	Avg.F_07/02
GY	2021_Walla_Walla	40	Avg.F_07/06
GY	2021_Walla_Walla	40	Max.F_06/28
GY	2021_Walla_Walla	40	Max.F_06/29
GY	2021_Walla_Walla	40	Max.F_06/30
GY	2021_Walla_Walla	40	Max.F_07/07
GY	2021_Walla_Walla	40	Max.F_07/08
GY	2021_Walla_Walla	40	Avg1.5m.RH._05/26
GY	2021_Walla_Walla	40	Avg1.5m.RH._06/22
GY	2021_Walla_Walla	40	Avg1.5m.RH._06/23
GY	2021_Walla_Walla	40	Avg1.5m.RH._06/29
GY	2021_Walla_Walla	40	Avg1.5m.RH._06/30
GY	2021_Walla_Walla	40	Avg1.5m.RH._07/02
GY	2021_Walla_Walla	40	Avg1.5m.RH._07/08
GY	2021_Walla_Walla	40	Min.F.1_07/01

GY	2021_Walla_Walla	40	Min.F.1_07/02
GY	2021_Walla_Walla	40	Min.F.1_07/03
GY	2021_Walla_Walla	40	Min.F.1_07/04
GY	2021_Walla_Walla	40	Avg.F.1_06/30
GY	2021_Walla_Walla	40	Avg.F.1_07/01
GY	2021_Walla_Walla	40	Avg.F.1_07/03
GY	2021_Walla_Walla	40	Avg.F.1_07/04
GY	2021_Walla_Walla	40	TotalSolarRadMJ.m._12/08
GY	2021_Walla_Walla	40	TotalSolarRadMJ.m._12/23
GY	2021_Walla_Walla	40	ETоin_03/31
GY	2021_Walla_Walla	40	ETоin_05/26
GY	2021_Walla_Walla	40	ETоin_06/29
GY	2021_Walla_Walla	40	ETоin_07/02
GY	2021_Walla_Walla	40	ETоin_07/05
GY	2021_Walla_Walla	40	ETоin_07/08
GY	2021_Walla_Walla	40	ETrin_12/12
GY	2021_Walla_Walla	40	ETrin_03/31
GY	2021_Walla_Walla	40	ETrin_05/26
GY	2021_Walla_Walla	40	ETrin_06/24
GY	2021_Walla_Walla	40	ETrin_06/29
GY	2021_Walla_Walla	40	ETrin_07/02
GY	2021_Walla_Walla	40	ETrin_07/05
GY	2021_Walla_Walla	40	ETrin_07/08
GY	2022_Davenport	2	Avg1.5m.RH._06/29
GY	2022_Davenport	2	ETrin_07/08
GY	2022_Farmington	2	Avg1.5m.RH._06/29
GY	2022_Farmington	2	ETrin_07/08
GY	2022_Harrington	1	Avg1.5m.RH._06/29
GY	2022_Prescott	1	Avg1.5m.RH._06/29
GY	2022_Pullman	11	Min.F_04/06
GY	2022_Pullman	11	Min.F_05/09
GY	2022_Pullman	11	Avg1.5m.DP.F_04/06
GY	2022_Pullman	11	Avg1.5m.RH._03/30
GY	2022_Pullman	11	Avg1.5m.RH._03/31
GY	2022_Pullman	11	Avg1.5m.RH._05/26
GY	2022_Pullman	11	Avg1.5m.RH._06/28
GY	2022_Pullman	11	Avg1.5m.RH._06/29
GY	2022_Pullman	11	TotPrecin_03/29
GY	2022_Pullman	11	ETrin_01/29
GY	2022_Pullman	11	ETrin_05/04
GY	2022_Ritzville	1	Avg1.5m.RH._06/29
PH	2019_Kincaid	135	Min.F_10/18
PH	2019_Kincaid	135	Min.F_11/02
PH	2019_Kincaid	135	Min.F_11/03

PH	2019_Kincaid	135	Min.F_11/16
PH	2019_Kincaid	135	Min.F_01/31
PH	2019_Kincaid	135	Min.F_02/23
PH	2019_Kincaid	135	Min.F_02/24
PH	2019_Kincaid	135	Min.F_02/26
PH	2019_Kincaid	135	Min.F_04/22
PH	2019_Kincaid	135	Min.F_06/12
PH	2019_Kincaid	135	Avg.F_11/18
PH	2019_Kincaid	135	Avg.F_11/19
PH	2019_Kincaid	135	Avg.F_12/21
PH	2019_Kincaid	135	Avg.F_01/03
PH	2019_Kincaid	135	Avg.F_01/07
PH	2019_Kincaid	135	Avg.F_01/08
PH	2019_Kincaid	135	Avg.F_01/29
PH	2019_Kincaid	135	Avg.F_01/30
PH	2019_Kincaid	135	Avg.F_01/31
PH	2019_Kincaid	135	Avg.F_02/25
PH	2019_Kincaid	135	Avg.F_02/26
PH	2019_Kincaid	135	Avg.F_03/06
PH	2019_Kincaid	135	Avg.F_03/09
PH	2019_Kincaid	135	Avg.F_03/10
PH	2019_Kincaid	135	Avg.F_03/11
PH	2019_Kincaid	135	Avg.F_04/27
PH	2019_Kincaid	135	Avg.F_04/28
PH	2019_Kincaid	135	Avg.F_04/29
PH	2019_Kincaid	135	Avg.F_05/02
PH	2019_Kincaid	135	Avg.F_06/24
PH	2019_Kincaid	135	Max.F_11/18
PH	2019_Kincaid	135	Max.F_12/21
PH	2019_Kincaid	135	Max.F_12/22
PH	2019_Kincaid	135	Max.F_01/01
PH	2019_Kincaid	135	Max.F_01/02
PH	2019_Kincaid	135	Max.F_01/03
PH	2019_Kincaid	135	Max.F_01/04
PH	2019_Kincaid	135	Max.F_02/21
PH	2019_Kincaid	135	Max.F_02/22
PH	2019_Kincaid	135	Max.F_02/25
PH	2019_Kincaid	135	Max.F_02/26
PH	2019_Kincaid	135	Max.F_03/03
PH	2019_Kincaid	135	Max.F_03/05
PH	2019_Kincaid	135	Max.F_03/06
PH	2019_Kincaid	135	Max.F_03/10
PH	2019_Kincaid	135	Max.F_03/11
PH	2019_Kincaid	135	Max.F_04/09

PH	2019_Kincaid	135	Max.F_04/20
PH	2019_Kincaid	135	Max.F_04/27
PH	2019_Kincaid	135	Max.F_04/28
PH	2019_Kincaid	135	Max.F_05/02
PH	2019_Kincaid	135	Max.F_05/24
PH	2019_Kincaid	135	Max.F_06/23
PH	2019_Kincaid	135	Avg1.5m.DP.F_11/17
PH	2019_Kincaid	135	Avg1.5m.DP.F_11/18
PH	2019_Kincaid	135	Avg1.5m.DP.F_12/09
PH	2019_Kincaid	135	Avg1.5m.DP.F_12/14
PH	2019_Kincaid	135	Avg1.5m.DP.F_01/07
PH	2019_Kincaid	135	Avg1.5m.DP.F_01/08
PH	2019_Kincaid	135	Avg1.5m.DP.F_02/26
PH	2019_Kincaid	135	Avg1.5m.DP.F_03/06
PH	2019_Kincaid	135	Avg1.5m.DP.F_03/07
PH	2019_Kincaid	135	Avg1.5m.DP.F_03/10
PH	2019_Kincaid	135	Avg1.5m.DP.F_04/24
PH	2019_Kincaid	135	Avg1.5m.DP.F_04/25
PH	2019_Kincaid	135	Avg1.5m.DP.F_04/27
PH	2019_Kincaid	135	Avg1.5m.DP.F_04/28
PH	2019_Kincaid	135	Avg1.5m.DP.F_04/29
PH	2019_Kincaid	135	Avg1.5m.DP.F_05/13
PH	2019_Kincaid	135	Avg1.5m.DP.F_06/23
PH	2019_Kincaid	135	Avg1.5m.DP.F_06/24
PH	2019_Kincaid	135	Avg1.5m.DP.F_06/25
PH	2019_Kincaid	135	Avg1.5m.DP.F_06/26
PH	2019_Kincaid	135	Avg1.5m.RH._12/29
PH	2019_Kincaid	135	Avg1.5m.RH._12/30
PH	2019_Kincaid	135	Avg1.5m.RH._03/01
PH	2019_Kincaid	135	Min.F.1_11/18
PH	2019_Kincaid	135	Min.F.1_11/19
PH	2019_Kincaid	135	Min.F.1_11/20
PH	2019_Kincaid	135	Min.F.1_12/23
PH	2019_Kincaid	135	Min.F.1_01/02
PH	2019_Kincaid	135	Min.F.1_01/03
PH	2019_Kincaid	135	Min.F.1_01/04
PH	2019_Kincaid	135	Min.F.1_01/05
PH	2019_Kincaid	135	Min.F.1_01/07
PH	2019_Kincaid	135	Min.F.1_01/08
PH	2019_Kincaid	135	Min.F.1_01/09
PH	2019_Kincaid	135	Min.F.1_01/10
PH	2019_Kincaid	135	Min.F.1_02/09
PH	2019_Kincaid	135	Min.F.1_02/27
PH	2019_Kincaid	135	Min.F.1_02/28

PH	2019_Kincaid	135	Min.F.1_03/01
PH	2019_Kincaid	135	Min.F.1_03/10
PH	2019_Kincaid	135	Min.F.1_03/11
PH	2019_Kincaid	135	Min.F.1_03/12
PH	2019_Kincaid	135	Min.F.1_04/10
PH	2019_Kincaid	135	Min.F.1_04/28
PH	2019_Kincaid	135	Min.F.1_04/29
PH	2019_Kincaid	135	Avg.F.1_11/18
PH	2019_Kincaid	135	Avg.F.1_11/19
PH	2019_Kincaid	135	Avg.F.1_11/20
PH	2019_Kincaid	135	Avg.F.1_11/21
PH	2019_Kincaid	135	Avg.F.1_12/23
PH	2019_Kincaid	135	Avg.F.1_01/02
PH	2019_Kincaid	135	Avg.F.1_01/03
PH	2019_Kincaid	135	Avg.F.1_01/04
PH	2019_Kincaid	135	Avg.F.1_01/05
PH	2019_Kincaid	135	Avg.F.1_01/06
PH	2019_Kincaid	135	Avg.F.1_01/07
PH	2019_Kincaid	135	Avg.F.1_01/08
PH	2019_Kincaid	135	Avg.F.1_01/09
PH	2019_Kincaid	135	Avg.F.1_01/10
PH	2019_Kincaid	135	Avg.F.1_02/26
PH	2019_Kincaid	135	Avg.F.1_02/27
PH	2019_Kincaid	135	Avg.F.1_02/28
PH	2019_Kincaid	135	Avg.F.1_03/01
PH	2019_Kincaid	135	Avg.F.1_03/10
PH	2019_Kincaid	135	Avg.F.1_03/11
PH	2019_Kincaid	135	Avg.F.1_03/12
PH	2019_Kincaid	135	Avg.F.1_04/10
PH	2019_Kincaid	135	TotalSolarRadMJ.m._10/21
PH	2019_Kincaid	135	TotalSolarRadMJ.m._12/15
PH	2019_Kincaid	135	TotalSolarRadMJ.m._12/31
PH	2019_Kincaid	135	TotalSolarRadMJ.m._01/06
PH	2019_Kincaid	135	TotalSolarRadMJ.m._01/10
PH	2019_Kincaid	135	TotalSolarRadMJ.m._02/06
PH	2019_Kincaid	135	TotalSolarRadMJ.m._07/01
PH	2019_Kincaid	135	EToin_10/20
PH	2019_Kincaid	135	EToin_02/24
PH	2019_Kincaid	135	EToin_03/02
PH	2019_Kincaid	135	ETrin_10/20
PH	2019_Kincaid	135	ETrin_01/09
PH	2019_Kincaid	135	ETrin_02/23
PH	2019_Kincaid	135	ETrin_02/24
PH	2019_Kincaid	135	ETrin_03/02

PH	2019_Pullman	141	Min.F_11/02
PH	2019_Pullman	141	Min.F_11/03
PH	2019_Pullman	141	Min.F_11/16
PH	2019_Pullman	141	Min.F_12/21
PH	2019_Pullman	141	Min.F_01/31
PH	2019_Pullman	141	Min.F_02/23
PH	2019_Pullman	141	Min.F_02/24
PH	2019_Pullman	141	Min.F_04/22
PH	2019_Pullman	141	Min.F_06/12
PH	2019_Pullman	141	Min.F_06/24
PH	2019_Pullman	141	Avg.F_11/18
PH	2019_Pullman	141	Avg.F_11/19
PH	2019_Pullman	141	Avg.F_12/21
PH	2019_Pullman	141	Avg.F_01/02
PH	2019_Pullman	141	Avg.F_01/03
PH	2019_Pullman	141	Avg.F_01/07
PH	2019_Pullman	141	Avg.F_01/08
PH	2019_Pullman	141	Avg.F_01/29
PH	2019_Pullman	141	Avg.F_01/30
PH	2019_Pullman	141	Avg.F_01/31
PH	2019_Pullman	141	Avg.F_02/24
PH	2019_Pullman	141	Avg.F_02/25
PH	2019_Pullman	141	Avg.F_03/06
PH	2019_Pullman	141	Avg.F_04/27
PH	2019_Pullman	141	Avg.F_04/28
PH	2019_Pullman	141	Avg.F_05/02
PH	2019_Pullman	141	Avg.F_06/24
PH	2019_Pullman	141	Max.F_11/18
PH	2019_Pullman	141	Max.F_12/21
PH	2019_Pullman	141	Max.F_12/22
PH	2019_Pullman	141	Max.F_12/31
PH	2019_Pullman	141	Max.F_01/01
PH	2019_Pullman	141	Max.F_01/02
PH	2019_Pullman	141	Max.F_01/03
PH	2019_Pullman	141	Max.F_01/04
PH	2019_Pullman	141	Max.F_02/22
PH	2019_Pullman	141	Max.F_02/24
PH	2019_Pullman	141	Max.F_02/25
PH	2019_Pullman	141	Max.F_02/26
PH	2019_Pullman	141	Max.F_03/05
PH	2019_Pullman	141	Max.F_03/06
PH	2019_Pullman	141	Max.F_03/09
PH	2019_Pullman	141	Max.F_03/10
PH	2019_Pullman	141	Max.F_03/11

PH	2019_Pullman	141	Max.F_04/09
PH	2019_Pullman	141	Max.F_04/20
PH	2019_Pullman	141	Max.F_04/27
PH	2019_Pullman	141	Max.F_04/28
PH	2019_Pullman	141	Max.F_05/02
PH	2019_Pullman	141	Max.F_05/05
PH	2019_Pullman	141	Max.F_05/24
PH	2019_Pullman	141	Max.F_06/19
PH	2019_Pullman	141	Max.F_06/23
PH	2019_Pullman	141	Avg1.5m.DP.F_11/17
PH	2019_Pullman	141	Avg1.5m.DP.F_11/18
PH	2019_Pullman	141	Avg1.5m.DP.F_12/09
PH	2019_Pullman	141	Avg1.5m.DP.F_12/31
PH	2019_Pullman	141	Avg1.5m.DP.F_01/02
PH	2019_Pullman	141	Avg1.5m.DP.F_01/08
PH	2019_Pullman	141	Avg1.5m.DP.F_01/29
PH	2019_Pullman	141	Avg1.5m.DP.F_01/30
PH	2019_Pullman	141	Avg1.5m.DP.F_02/26
PH	2019_Pullman	141	Avg1.5m.DP.F_03/06
PH	2019_Pullman	141	Avg1.5m.DP.F_04/24
PH	2019_Pullman	141	Avg1.5m.DP.F_05/10
PH	2019_Pullman	141	Avg1.5m.DP.F_05/13
PH	2019_Pullman	141	Avg1.5m.DP.F_06/23
PH	2019_Pullman	141	Avg1.5m.DP.F_06/24
PH	2019_Pullman	141	Avg1.5m.DP.F_06/25
PH	2019_Pullman	141	Avg1.5m.DP.F_06/26
PH	2019_Pullman	141	Avg1.5m.RH._03/01
PH	2019_Pullman	141	Min.F.1_11/18
PH	2019_Pullman	141	Min.F.1_11/19
PH	2019_Pullman	141	Min.F.1_11/20
PH	2019_Pullman	141	Min.F.1_12/22
PH	2019_Pullman	141	Min.F.1_12/23
PH	2019_Pullman	141	Min.F.1_01/02
PH	2019_Pullman	141	Min.F.1_01/03
PH	2019_Pullman	141	Min.F.1_01/04
PH	2019_Pullman	141	Min.F.1_01/05
PH	2019_Pullman	141	Min.F.1_01/06
PH	2019_Pullman	141	Min.F.1_01/07
PH	2019_Pullman	141	Min.F.1_01/08
PH	2019_Pullman	141	Min.F.1_01/09
PH	2019_Pullman	141	Min.F.1_01/10
PH	2019_Pullman	141	Min.F.1_01/12
PH	2019_Pullman	141	Min.F.1_01/31
PH	2019_Pullman	141	Min.F.1_02/01

PH	2019_Pullman	141	Min.F.1_02/09
PH	2019_Pullman	141	Min.F.1_02/26
PH	2019_Pullman	141	Min.F.1_02/27
PH	2019_Pullman	141	Min.F.1_02/28
PH	2019_Pullman	141	Min.F.1_03/01
PH	2019_Pullman	141	Min.F.1_03/02
PH	2019_Pullman	141	Min.F.1_03/11
PH	2019_Pullman	141	Min.F.1_03/12
PH	2019_Pullman	141	Min.F.1_04/10
PH	2019_Pullman	141	Min.F.1_04/15
PH	2019_Pullman	141	Min.F.1_04/27
PH	2019_Pullman	141	Min.F.1_04/28
PH	2019_Pullman	141	Min.F.1_04/29
PH	2019_Pullman	141	Min.F.1_05/03
PH	2019_Pullman	141	Avg.F.1_11/18
PH	2019_Pullman	141	Avg.F.1_11/19
PH	2019_Pullman	141	Avg.F.1_11/20
PH	2019_Pullman	141	Avg.F.1_12/23
PH	2019_Pullman	141	Avg.F.1_12/24
PH	2019_Pullman	141	Avg.F.1_01/02
PH	2019_Pullman	141	Avg.F.1_01/03
PH	2019_Pullman	141	Avg.F.1_01/04
PH	2019_Pullman	141	Avg.F.1_01/05
PH	2019_Pullman	141	Avg.F.1_01/06
PH	2019_Pullman	141	Avg.F.1_01/07
PH	2019_Pullman	141	Avg.F.1_01/08
PH	2019_Pullman	141	Avg.F.1_01/09
PH	2019_Pullman	141	Avg.F.1_01/10
PH	2019_Pullman	141	Avg.F.1_01/11
PH	2019_Pullman	141	Avg.F.1_01/12
PH	2019_Pullman	141	Avg.F.1_01/31
PH	2019_Pullman	141	Avg.F.1_02/01
PH	2019_Pullman	141	Avg.F.1_02/09
PH	2019_Pullman	141	Avg.F.1_02/26
PH	2019_Pullman	141	Avg.F.1_02/27
PH	2019_Pullman	141	Avg.F.1_02/28
PH	2019_Pullman	141	Avg.F.1_03/01
PH	2019_Pullman	141	Avg.F.1_03/11
PH	2019_Pullman	141	Avg.F.1_04/10
PH	2019_Pullman	141	Avg.F.1_04/11
PH	2019_Pullman	141	Avg.F.1_04/27
PH	2019_Pullman	141	Avg.F.1_04/28
PH	2019_Pullman	141	TotalSolarRadMJ.m._12/15
PH	2019_Pullman	141	TotalSolarRadMJ.m._12/31

PH	2019_Pullman	141	TotalSolarRadMJ.m._01/06
PH	2019_Pullman	141	TotalSolarRadMJ.m._01/10
PH	2019_Pullman	141	TotalSolarRadMJ.m._07/01
PH	2019_Pullman	141	EToin_02/23
PH	2019_Pullman	141	EToin_02/24
PH	2019_Pullman	141	EToin_03/02
PH	2019_Pullman	141	ETrin_02/23
PH	2019_Pullman	141	ETrin_02/24
PH	2019_Pullman	141	ETrin_03/02
PH	2020_Farmington	221	Min.F_10/18
PH	2020_Farmington	221	Min.F_10/27
PH	2020_Farmington	221	Min.F_11/02
PH	2020_Farmington	221	Min.F_11/03
PH	2020_Farmington	221	Min.F_11/16
PH	2020_Farmington	221	Min.F_12/21
PH	2020_Farmington	221	Min.F_01/02
PH	2020_Farmington	221	Min.F_01/08
PH	2020_Farmington	221	Min.F_01/29
PH	2020_Farmington	221	Min.F_01/31
PH	2020_Farmington	221	Min.F_02/23
PH	2020_Farmington	221	Min.F_02/24
PH	2020_Farmington	221	Min.F_02/26
PH	2020_Farmington	221	Min.F_04/22
PH	2020_Farmington	221	Min.F_06/12
PH	2020_Farmington	221	Min.F_06/24
PH	2020_Farmington	221	Min.F_06/25
PH	2020_Farmington	221	Avg.F_11/02
PH	2020_Farmington	221	Avg.F_11/17
PH	2020_Farmington	221	Avg.F_11/18
PH	2020_Farmington	221	Avg.F_11/19
PH	2020_Farmington	221	Avg.F_12/21
PH	2020_Farmington	221	Avg.F_01/02
PH	2020_Farmington	221	Avg.F_01/03
PH	2020_Farmington	221	Avg.F_01/07
PH	2020_Farmington	221	Avg.F_01/08
PH	2020_Farmington	221	Avg.F_01/28
PH	2020_Farmington	221	Avg.F_01/29
PH	2020_Farmington	221	Avg.F_01/30
PH	2020_Farmington	221	Avg.F_01/31
PH	2020_Farmington	221	Avg.F_02/21
PH	2020_Farmington	221	Avg.F_02/22
PH	2020_Farmington	221	Avg.F_02/24
PH	2020_Farmington	221	Avg.F_02/25
PH	2020_Farmington	221	Avg.F_02/26

PH	2020_Farmington	221	Avg.F_03/06
PH	2020_Farmington	221	Avg.F_03/09
PH	2020_Farmington	221	Avg.F_03/10
PH	2020_Farmington	221	Avg.F_03/11
PH	2020_Farmington	221	Avg.F_04/06
PH	2020_Farmington	221	Avg.F_04/27
PH	2020_Farmington	221	Avg.F_04/28
PH	2020_Farmington	221	Avg.F_04/29
PH	2020_Farmington	221	Avg.F_04/30
PH	2020_Farmington	221	Avg.F_05/02
PH	2020_Farmington	221	Avg.F_06/24
PH	2020_Farmington	221	Max.F_11/17
PH	2020_Farmington	221	Max.F_11/18
PH	2020_Farmington	221	Max.F_12/21
PH	2020_Farmington	221	Max.F_12/22
PH	2020_Farmington	221	Max.F_12/31
PH	2020_Farmington	221	Max.F_01/01
PH	2020_Farmington	221	Max.F_01/02
PH	2020_Farmington	221	Max.F_01/03
PH	2020_Farmington	221	Max.F_01/04
PH	2020_Farmington	221	Max.F_01/07
PH	2020_Farmington	221	Max.F_01/08
PH	2020_Farmington	221	Max.F_01/30
PH	2020_Farmington	221	Max.F_02/21
PH	2020_Farmington	221	Max.F_02/22
PH	2020_Farmington	221	Max.F_02/24
PH	2020_Farmington	221	Max.F_02/25
PH	2020_Farmington	221	Max.F_02/26
PH	2020_Farmington	221	Max.F_02/27
PH	2020_Farmington	221	Max.F_03/03
PH	2020_Farmington	221	Max.F_03/05
PH	2020_Farmington	221	Max.F_03/06
PH	2020_Farmington	221	Max.F_03/09
PH	2020_Farmington	221	Max.F_03/10
PH	2020_Farmington	221	Max.F_03/11
PH	2020_Farmington	221	Max.F_04/06
PH	2020_Farmington	221	Max.F_04/09
PH	2020_Farmington	221	Max.F_04/20
PH	2020_Farmington	221	Max.F_04/27
PH	2020_Farmington	221	Max.F_04/28
PH	2020_Farmington	221	Max.F_04/29
PH	2020_Farmington	221	Max.F_05/02
PH	2020_Farmington	221	Max.F_05/05
PH	2020_Farmington	221	Max.F_05/24

PH	2020_Farmington	221	Max.F_06/19
PH	2020_Farmington	221	Max.F_06/23
PH	2020_Farmington	221	Max.F_06/24
PH	2020_Farmington	221	Avg1.5m.DP.F_10/17
PH	2020_Farmington	221	Avg1.5m.DP.F_10/19
PH	2020_Farmington	221	Avg1.5m.DP.F_11/17
PH	2020_Farmington	221	Avg1.5m.DP.F_11/18
PH	2020_Farmington	221	Avg1.5m.DP.F_12/09
PH	2020_Farmington	221	Avg1.5m.DP.F_12/14
PH	2020_Farmington	221	Avg1.5m.DP.F_12/31
PH	2020_Farmington	221	Avg1.5m.DP.F_01/02
PH	2020_Farmington	221	Avg1.5m.DP.F_01/03
PH	2020_Farmington	221	Avg1.5m.DP.F_01/06
PH	2020_Farmington	221	Avg1.5m.DP.F_01/07
PH	2020_Farmington	221	Avg1.5m.DP.F_01/08
PH	2020_Farmington	221	Avg1.5m.DP.F_01/30
PH	2020_Farmington	221	Avg1.5m.DP.F_01/31
PH	2020_Farmington	221	Avg1.5m.DP.F_02/26
PH	2020_Farmington	221	Avg1.5m.DP.F_03/06
PH	2020_Farmington	221	Avg1.5m.DP.F_03/07
PH	2020_Farmington	221	Avg1.5m.DP.F_03/09
PH	2020_Farmington	221	Avg1.5m.DP.F_03/10
PH	2020_Farmington	221	Avg1.5m.DP.F_04/24
PH	2020_Farmington	221	Avg1.5m.DP.F_04/25
PH	2020_Farmington	221	Avg1.5m.DP.F_04/27
PH	2020_Farmington	221	Avg1.5m.DP.F_04/28
PH	2020_Farmington	221	Avg1.5m.DP.F_04/29
PH	2020_Farmington	221	Avg1.5m.DP.F_05/10
PH	2020_Farmington	221	Avg1.5m.DP.F_05/13
PH	2020_Farmington	221	Avg1.5m.DP.F_06/15
PH	2020_Farmington	221	Avg1.5m.DP.F_06/23
PH	2020_Farmington	221	Avg1.5m.DP.F_06/24
PH	2020_Farmington	221	Avg1.5m.DP.F_06/25
PH	2020_Farmington	221	Avg1.5m.DP.F_06/26
PH	2020_Farmington	221	Avg1.5m.DP.F_06/29
PH	2020_Farmington	221	Avg1.5m.RH._12/29
PH	2020_Farmington	221	Avg1.5m.RH._12/30
PH	2020_Farmington	221	Avg1.5m.RH._02/28
PH	2020_Farmington	221	Avg1.5m.RH._03/01
PH	2020_Farmington	221	Avg1.5m.RH._03/02
PH	2020_Farmington	221	Avg1.5m.RH._03/23
PH	2020_Farmington	221	Min.F.1_11/03
PH	2020_Farmington	221	Min.F.1_11/17
PH	2020_Farmington	221	Min.F.1_11/18

PH	2020_Farmington	221	Min.F.1_11/19
PH	2020_Farmington	221	Min.F.1_11/20
PH	2020_Farmington	221	Min.F.1_11/21
PH	2020_Farmington	221	Min.F.1_12/22
PH	2020_Farmington	221	Min.F.1_12/23
PH	2020_Farmington	221	Min.F.1_01/02
PH	2020_Farmington	221	Min.F.1_01/03
PH	2020_Farmington	221	Min.F.1_01/04
PH	2020_Farmington	221	Min.F.1_01/05
PH	2020_Farmington	221	Min.F.1_01/06
PH	2020_Farmington	221	Min.F.1_01/07
PH	2020_Farmington	221	Min.F.1_01/08
PH	2020_Farmington	221	Min.F.1_01/09
PH	2020_Farmington	221	Min.F.1_01/10
PH	2020_Farmington	221	Min.F.1_01/12
PH	2020_Farmington	221	Min.F.1_01/31
PH	2020_Farmington	221	Min.F.1_02/01
PH	2020_Farmington	221	Min.F.1_02/09
PH	2020_Farmington	221	Min.F.1_02/27
PH	2020_Farmington	221	Min.F.1_02/28
PH	2020_Farmington	221	Min.F.1_03/01
PH	2020_Farmington	221	Min.F.1_03/02
PH	2020_Farmington	221	Min.F.1_03/04
PH	2020_Farmington	221	Min.F.1_03/06
PH	2020_Farmington	221	Min.F.1_03/07
PH	2020_Farmington	221	Min.F.1_03/10
PH	2020_Farmington	221	Min.F.1_03/11
PH	2020_Farmington	221	Min.F.1_03/12
PH	2020_Farmington	221	Min.F.1_04/10
PH	2020_Farmington	221	Min.F.1_04/15
PH	2020_Farmington	221	Min.F.1_04/27
PH	2020_Farmington	221	Min.F.1_04/28
PH	2020_Farmington	221	Min.F.1_04/29
PH	2020_Farmington	221	Min.F.1_04/30
PH	2020_Farmington	221	Min.F.1_05/02
PH	2020_Farmington	221	Min.F.1_05/03
PH	2020_Farmington	221	Min.F.1_05/28
PH	2020_Farmington	221	Avg.F.1_11/18
PH	2020_Farmington	221	Avg.F.1_11/19
PH	2020_Farmington	221	Avg.F.1_11/20
PH	2020_Farmington	221	Avg.F.1_11/21
PH	2020_Farmington	221	Avg.F.1_12/23
PH	2020_Farmington	221	Avg.F.1_01/02
PH	2020_Farmington	221	Avg.F.1_01/03

PH	2020_Farmington	221	Avg.F.1_01/04
PH	2020_Farmington	221	Avg.F.1_01/05
PH	2020_Farmington	221	Avg.F.1_01/06
PH	2020_Farmington	221	Avg.F.1_01/07
PH	2020_Farmington	221	Avg.F.1_01/08
PH	2020_Farmington	221	Avg.F.1_01/09
PH	2020_Farmington	221	Avg.F.1_01/10
PH	2020_Farmington	221	Avg.F.1_01/11
PH	2020_Farmington	221	Avg.F.1_01/12
PH	2020_Farmington	221	Avg.F.1_01/31
PH	2020_Farmington	221	Avg.F.1_02/09
PH	2020_Farmington	221	Avg.F.1_02/26
PH	2020_Farmington	221	Avg.F.1_02/27
PH	2020_Farmington	221	Avg.F.1_02/28
PH	2020_Farmington	221	Avg.F.1_03/01
PH	2020_Farmington	221	Avg.F.1_03/02
PH	2020_Farmington	221	Avg.F.1_03/07
PH	2020_Farmington	221	Avg.F.1_03/10
PH	2020_Farmington	221	Avg.F.1_03/11
PH	2020_Farmington	221	Avg.F.1_03/12
PH	2020_Farmington	221	Avg.F.1_04/10
PH	2020_Farmington	221	Avg.F.1_04/27
PH	2020_Farmington	221	Avg.F.1_04/28
PH	2020_Farmington	221	Avg.F.1_04/29
PH	2020_Farmington	221	Avg.F.1_04/30
PH	2020_Farmington	221	Avg.F.1_05/02
PH	2020_Farmington	221	TotalSolarRadMJ.m._10/21
PH	2020_Farmington	221	TotalSolarRadMJ.m._11/17
PH	2020_Farmington	221	TotalSolarRadMJ.m._12/15
PH	2020_Farmington	221	TotalSolarRadMJ.m._12/31
PH	2020_Farmington	221	TotalSolarRadMJ.m._01/06
PH	2020_Farmington	221	TotalSolarRadMJ.m._01/10
PH	2020_Farmington	221	TotalSolarRadMJ.m._01/20
PH	2020_Farmington	221	TotalSolarRadMJ.m._02/06
PH	2020_Farmington	221	TotalSolarRadMJ.m._02/19
PH	2020_Farmington	221	TotalSolarRadMJ.m._02/23
PH	2020_Farmington	221	TotalSolarRadMJ.m._04/28
PH	2020_Farmington	221	TotalSolarRadMJ.m._07/01
PH	2020_Farmington	221	ETOin_10/20
PH	2020_Farmington	221	ETOin_02/22
PH	2020_Farmington	221	ETOin_02/23
PH	2020_Farmington	221	ETOin_02/24
PH	2020_Farmington	221	ETOin_03/02
PH	2020_Farmington	221	ETOin_03/03

PH	2020_Farmington	221	ETоin_03/04
PH	2020_Farmington	221	EToin_03/10
PH	2020_Farmington	221	EToin_03/11
PH	2020_Farmington	221	ETrin_10/20
PH	2020_Farmington	221	ETrin_01/09
PH	2020_Farmington	221	ETrin_02/23
PH	2020_Farmington	221	ETrin_02/24
PH	2020_Farmington	221	ETrin_03/02
PH	2020_Farmington	221	ETrin_03/03
PH	2020_Farmington	221	ETrin_03/04
PH	2020_Harrington	4	Min.F_06/12
PH	2020_Harrington	4	Max.F_01/03
PH	2020_Harrington	4	Avg1.5m.DP.F_04/24
PH	2020_Harrington	4	Avg1.5m.DP.F_05/13
PH	2020_Kincaid	6	Min.F_06/12
PH	2020_Kincaid	6	Max.F_01/03
PH	2020_Kincaid	6	Avg1.5m.DP.F_04/24
PH	2020_Kincaid	6	Avg1.5m.DP.F_05/13
PH	2020_Kincaid	6	Min.F.1_01/12
PH	2020_Kincaid	6	Avg.F.1_01/12
PH	2020_Ritzville	4	Min.F_06/12
PH	2020_Ritzville	4	Max.F_01/03
PH	2020_Ritzville	4	Avg1.5m.DP.F_04/24
PH	2020_Ritzville	4	Avg1.5m.DP.F_05/13
PH	2020_Walla_Walla	65	Min.F_11/03
PH	2020_Walla_Walla	65	Min.F_11/16
PH	2020_Walla_Walla	65	Min.F_01/31
PH	2020_Walla_Walla	65	Min.F_02/21
PH	2020_Walla_Walla	65	Min.F_02/23
PH	2020_Walla_Walla	65	Min.F_06/12
PH	2020_Walla_Walla	65	Avg.F_11/18
PH	2020_Walla_Walla	65	Avg.F_11/19
PH	2020_Walla_Walla	65	Avg.F_12/21
PH	2020_Walla_Walla	65	Avg.F_01/30
PH	2020_Walla_Walla	65	Avg.F_01/31
PH	2020_Walla_Walla	65	Avg.F_02/25
PH	2020_Walla_Walla	65	Avg.F_03/06
PH	2020_Walla_Walla	65	Avg.F_04/27
PH	2020_Walla_Walla	65	Avg.F_04/28
PH	2020_Walla_Walla	65	Avg.F_04/29
PH	2020_Walla_Walla	65	Max.F_11/05
PH	2020_Walla_Walla	65	Max.F_12/22
PH	2020_Walla_Walla	65	Max.F_01/03
PH	2020_Walla_Walla	65	Max.F_02/22

PH	2020_Walla_Walla	65	Max.F_02/25
PH	2020_Walla_Walla	65	Max.F_03/05
PH	2020_Walla_Walla	65	Max.F_03/06
PH	2020_Walla_Walla	65	Max.F_03/10
PH	2020_Walla_Walla	65	Max.F_03/11
PH	2020_Walla_Walla	65	Max.F_04/09
PH	2020_Walla_Walla	65	Max.F_04/20
PH	2020_Walla_Walla	65	Max.F_04/27
PH	2020_Walla_Walla	65	Max.F_04/28
PH	2020_Walla_Walla	65	Max.F_04/29
PH	2020_Walla_Walla	65	Max.F_06/23
PH	2020_Walla_Walla	65	Avg1.5m.DP.F_11/17
PH	2020_Walla_Walla	65	Avg1.5m.DP.F_11/18
PH	2020_Walla_Walla	65	Avg1.5m.DP.F_12/09
PH	2020_Walla_Walla	65	Avg1.5m.DP.F_01/03
PH	2020_Walla_Walla	65	Avg1.5m.DP.F_01/08
PH	2020_Walla_Walla	65	Avg1.5m.DP.F_04/24
PH	2020_Walla_Walla	65	Avg1.5m.DP.F_05/13
PH	2020_Walla_Walla	65	Avg1.5m.DP.F_06/23
PH	2020_Walla_Walla	65	Avg1.5m.DP.F_06/24
PH	2020_Walla_Walla	65	Avg1.5m.DP.F_06/25
PH	2020_Walla_Walla	65	Avg1.5m.RH._02/28
PH	2020_Walla_Walla	65	Avg1.5m.RH._03/01
PH	2020_Walla_Walla	65	Avg1.5m.RH._03/05
PH	2020_Walla_Walla	65	Min.F.1_11/18
PH	2020_Walla_Walla	65	Min.F.1_11/19
PH	2020_Walla_Walla	65	Min.F.1_01/08
PH	2020_Walla_Walla	65	Min.F.1_01/09
PH	2020_Walla_Walla	65	Min.F.1_03/11
PH	2020_Walla_Walla	65	Avg.F.1_11/18
PH	2020_Walla_Walla	65	Avg.F.1_11/19
PH	2020_Walla_Walla	65	Avg.F.1_11/20
PH	2020_Walla_Walla	65	Avg.F.1_01/04
PH	2020_Walla_Walla	65	Avg.F.1_01/07
PH	2020_Walla_Walla	65	Avg.F.1_01/08
PH	2020_Walla_Walla	65	Avg.F.1_01/09
PH	2020_Walla_Walla	65	Avg.F.1_03/11
PH	2020_Walla_Walla	65	TotalSolarRadMJ.m._12/05
PH	2020_Walla_Walla	65	TotalSolarRadMJ.m._12/15
PH	2020_Walla_Walla	65	TotalSolarRadMJ.m._12/31
PH	2020_Walla_Walla	65	TotalSolarRadMJ.m._07/01
PH	2020_Walla_Walla	65	EToin_02/24
PH	2020_Walla_Walla	65	EToin_03/05
PH	2020_Walla_Walla	65	ETrin_03/02

PH	2020_Walla_Walla	65	ETrin_03/05
PH	2021_Davenport	27	Min.F_06/12
PH	2021_Davenport	27	Avg.F_11/19
PH	2021_Davenport	27	Max.F_01/03
PH	2021_Davenport	27	Avg1.5m.DP.F_05/13
PH	2021_Davenport	27	Min.F.1_11/17
PH	2021_Davenport	27	Min.F.1_11/18
PH	2021_Davenport	27	Min.F.1_11/19
PH	2021_Davenport	27	Min.F.1_12/23
PH	2021_Davenport	27	Min.F.1_01/04
PH	2021_Davenport	27	Min.F.1_01/05
PH	2021_Davenport	27	Min.F.1_01/08
PH	2021_Davenport	27	Min.F.1_01/09
PH	2021_Davenport	27	Min.F.1_02/28
PH	2021_Davenport	27	Min.F.1_03/01
PH	2021_Davenport	27	Min.F.1_03/11
PH	2021_Davenport	27	Avg.F.1_11/18
PH	2021_Davenport	27	Avg.F.1_11/19
PH	2021_Davenport	27	Avg.F.1_12/23
PH	2021_Davenport	27	Avg.F.1_01/02
PH	2021_Davenport	27	Avg.F.1_01/03
PH	2021_Davenport	27	Avg.F.1_01/04
PH	2021_Davenport	27	Avg.F.1_01/05
PH	2021_Davenport	27	Avg.F.1_01/07
PH	2021_Davenport	27	Avg.F.1_01/08
PH	2021_Davenport	27	Avg.F.1_01/09
PH	2021_Davenport	27	Avg.F.1_02/27
PH	2021_Davenport	27	Avg.F.1_02/28
PH	2021_Harrington	5	Min.F_06/12
PH	2021_Harrington	5	Max.F_01/03
PH	2021_Harrington	5	Max.F_02/25
PH	2021_Harrington	5	Avg1.5m.DP.F_04/24
PH	2021_Harrington	5	Avg1.5m.DP.F_05/13
PH	2021_Kahlotus	4	Min.F_06/12
PH	2021_Kahlotus	4	Max.F_01/03
PH	2021_Kahlotus	4	Avg1.5m.DP.F_04/24
PH	2021_Kahlotus	4	Avg1.5m.DP.F_05/13
PH	2021_Kincaid	17	Min.F_11/02
PH	2021_Kincaid	17	Min.F_11/03
PH	2021_Kincaid	17	Min.F_06/12
PH	2021_Kincaid	17	Max.F_01/03
PH	2021_Kincaid	17	Max.F_03/06
PH	2021_Kincaid	17	Max.F_03/19
PH	2021_Kincaid	17	Max.F_04/20

PH	2021_Kincaid	17	Max.F_05/02
PH	2021_Kincaid	17	Avg1.5m.DP.F_04/24
PH	2021_Kincaid	17	Avg1.5m.DP.F_05/13
PH	2021_Kincaid	17	Min.F.1_01/03
PH	2021_Kincaid	17	Min.F.1_03/01
PH	2021_Kincaid	17	Avg.F.1_01/02
PH	2021_Kincaid	17	Avg.F.1_01/03
PH	2021_Kincaid	17	TotalSolarRadMJ.m._07/01
PH	2021_Kincaid	17	EToin_02/24
PH	2021_Kincaid	17	ETrin_02/24
PH	2021_Pullman	180	Min.F_10/18
PH	2021_Pullman	180	Min.F_10/19
PH	2021_Pullman	180	Min.F_10/26
PH	2021_Pullman	180	Min.F_11/16
PH	2021_Pullman	180	Min.F_11/17
PH	2021_Pullman	180	Min.F_11/18
PH	2021_Pullman	180	Min.F_12/21
PH	2021_Pullman	180	Min.F_01/02
PH	2021_Pullman	180	Min.F_01/08
PH	2021_Pullman	180	Min.F_01/28
PH	2021_Pullman	180	Min.F_01/29
PH	2021_Pullman	180	Min.F_01/31
PH	2021_Pullman	180	Min.F_02/21
PH	2021_Pullman	180	Min.F_02/22
PH	2021_Pullman	180	Min.F_02/23
PH	2021_Pullman	180	Min.F_02/24
PH	2021_Pullman	180	Min.F_02/26
PH	2021_Pullman	180	Min.F_04/29
PH	2021_Pullman	180	Min.F_04/30
PH	2021_Pullman	180	Min.F_06/12
PH	2021_Pullman	180	Min.F_06/20
PH	2021_Pullman	180	Min.F_06/25
PH	2021_Pullman	180	Avg.F_10/27
PH	2021_Pullman	180	Avg.F_11/17
PH	2021_Pullman	180	Avg.F_11/18
PH	2021_Pullman	180	Avg.F_11/19
PH	2021_Pullman	180	Avg.F_12/21
PH	2021_Pullman	180	Avg.F_01/02
PH	2021_Pullman	180	Avg.F_01/03
PH	2021_Pullman	180	Avg.F_01/30
PH	2021_Pullman	180	Avg.F_01/31
PH	2021_Pullman	180	Avg.F_02/21
PH	2021_Pullman	180	Avg.F_02/22
PH	2021_Pullman	180	Avg.F_02/25

PH	2021_Pullman	180	Avg.F_02/26
PH	2021_Pullman	180	Avg.F_03/09
PH	2021_Pullman	180	Avg.F_03/10
PH	2021_Pullman	180	Avg.F_04/27
PH	2021_Pullman	180	Avg.F_04/28
PH	2021_Pullman	180	Avg.F_04/29
PH	2021_Pullman	180	Avg.F_04/30
PH	2021_Pullman	180	Avg.F_05/01
PH	2021_Pullman	180	Avg.F_06/20
PH	2021_Pullman	180	Avg.F_06/23
PH	2021_Pullman	180	Avg.F_06/24
PH	2021_Pullman	180	Max.F_11/05
PH	2021_Pullman	180	Max.F_11/17
PH	2021_Pullman	180	Max.F_12/21
PH	2021_Pullman	180	Max.F_01/01
PH	2021_Pullman	180	Max.F_01/02
PH	2021_Pullman	180	Max.F_01/03
PH	2021_Pullman	180	Max.F_01/30
PH	2021_Pullman	180	Max.F_02/21
PH	2021_Pullman	180	Max.F_02/22
PH	2021_Pullman	180	Max.F_02/25
PH	2021_Pullman	180	Max.F_03/04
PH	2021_Pullman	180	Max.F_03/05
PH	2021_Pullman	180	Max.F_03/09
PH	2021_Pullman	180	Max.F_03/10
PH	2021_Pullman	180	Max.F_03/11
PH	2021_Pullman	180	Max.F_04/09
PH	2021_Pullman	180	Max.F_04/20
PH	2021_Pullman	180	Max.F_04/27
PH	2021_Pullman	180	Max.F_04/28
PH	2021_Pullman	180	Max.F_04/29
PH	2021_Pullman	180	Max.F_05/17
PH	2021_Pullman	180	Max.F_06/19
PH	2021_Pullman	180	Max.F_06/20
PH	2021_Pullman	180	Max.F_06/23
PH	2021_Pullman	180	Max.F_06/24
PH	2021_Pullman	180	Avg1.5m.DP.F_10/17
PH	2021_Pullman	180	Avg1.5m.DP.F_10/18
PH	2021_Pullman	180	Avg1.5m.DP.F_10/19
PH	2021_Pullman	180	Avg1.5m.DP.F_11/17
PH	2021_Pullman	180	Avg1.5m.DP.F_11/18
PH	2021_Pullman	180	Avg1.5m.DP.F_12/09
PH	2021_Pullman	180	Avg1.5m.DP.F_12/14
PH	2021_Pullman	180	Avg1.5m.DP.F_12/21

PH	2021_Pullman	180	Avg1.5m.DP.F_12/31
PH	2021_Pullman	180	Avg1.5m.DP.F_01/02
PH	2021_Pullman	180	Avg1.5m.DP.F_01/03
PH	2021_Pullman	180	Avg1.5m.DP.F_01/08
PH	2021_Pullman	180	Avg1.5m.DP.F_02/21
PH	2021_Pullman	180	Avg1.5m.DP.F_02/26
PH	2021_Pullman	180	Avg1.5m.DP.F_03/06
PH	2021_Pullman	180	Avg1.5m.DP.F_03/09
PH	2021_Pullman	180	Avg1.5m.DP.F_03/10
PH	2021_Pullman	180	Avg1.5m.DP.F_04/24
PH	2021_Pullman	180	Avg1.5m.DP.F_04/28
PH	2021_Pullman	180	Avg1.5m.DP.F_05/13
PH	2021_Pullman	180	Avg1.5m.DP.F_06/15
PH	2021_Pullman	180	Avg1.5m.DP.F_06/23
PH	2021_Pullman	180	Avg1.5m.DP.F_06/24
PH	2021_Pullman	180	Avg1.5m.DP.F_06/25
PH	2021_Pullman	180	Avg1.5m.DP.F_06/26
PH	2021_Pullman	180	Avg1.5m.DP.F_06/29
PH	2021_Pullman	180	Avg1.5m.RH._01/09
PH	2021_Pullman	180	Avg1.5m.RH._01/24
PH	2021_Pullman	180	Avg1.5m.RH._01/27
PH	2021_Pullman	180	Avg1.5m.RH._03/01
PH	2021_Pullman	180	Avg1.5m.RH._03/02
PH	2021_Pullman	180	Avg1.5m.RH._04/14
PH	2021_Pullman	180	Avg1.5m.RH._04/25
PH	2021_Pullman	180	Min.F.1_11/18
PH	2021_Pullman	180	Min.F.1_11/19
PH	2021_Pullman	180	Min.F.1_12/22
PH	2021_Pullman	180	Min.F.1_01/06
PH	2021_Pullman	180	Min.F.1_01/07
PH	2021_Pullman	180	Min.F.1_01/08
PH	2021_Pullman	180	Min.F.1_01/09
PH	2021_Pullman	180	Min.F.1_01/10
PH	2021_Pullman	180	Min.F.1_01/13
PH	2021_Pullman	180	Min.F.1_03/10
PH	2021_Pullman	180	Min.F.1_03/11
PH	2021_Pullman	180	Min.F.1_03/12
PH	2021_Pullman	180	Min.F.1_04/28
PH	2021_Pullman	180	Min.F.1_04/29
PH	2021_Pullman	180	Min.F.1_04/30
PH	2021_Pullman	180	Min.F.1_05/01
PH	2021_Pullman	180	Min.F.1_05/02
PH	2021_Pullman	180	Min.F.1_05/03
PH	2021_Pullman	180	Min.F.1_05/18

PH	2021_Pullman	180	Min.F.1_06/24
PH	2021_Pullman	180	Min.F.1_06/25
PH	2021_Pullman	180	Min.F.1_06/27
PH	2021_Pullman	180	Avg.F.1_11/18
PH	2021_Pullman	180	Avg.F.1_11/19
PH	2021_Pullman	180	Avg.F.1_12/22
PH	2021_Pullman	180	Avg.F.1_12/23
PH	2021_Pullman	180	Avg.F.1_01/04
PH	2021_Pullman	180	Avg.F.1_01/05
PH	2021_Pullman	180	Avg.F.1_01/06
PH	2021_Pullman	180	Avg.F.1_01/07
PH	2021_Pullman	180	Avg.F.1_01/08
PH	2021_Pullman	180	Avg.F.1_01/09
PH	2021_Pullman	180	Avg.F.1_01/10
PH	2021_Pullman	180	Avg.F.1_01/13
PH	2021_Pullman	180	Avg.F.1_03/10
PH	2021_Pullman	180	Avg.F.1_03/11
PH	2021_Pullman	180	Avg.F.1_03/12
PH	2021_Pullman	180	Avg.F.1_04/27
PH	2021_Pullman	180	Avg.F.1_04/28
PH	2021_Pullman	180	Avg.F.1_04/29
PH	2021_Pullman	180	Avg.F.1_04/30
PH	2021_Pullman	180	Avg.F.1_05/01
PH	2021_Pullman	180	Avg.F.1_05/02
PH	2021_Pullman	180	Avg.F.1_05/03
PH	2021_Pullman	180	Avg.F.1_06/24
PH	2021_Pullman	180	TotPrecin_11/04
PH	2021_Pullman	180	TotPrecin_04/11
PH	2021_Pullman	180	TotPrecin_04/19
PH	2021_Pullman	180	TotPrecin_05/26
PH	2021_Pullman	180	TotalSolarRadMJ.m._10/17
PH	2021_Pullman	180	TotalSolarRadMJ.m._10/18
PH	2021_Pullman	180	TotalSolarRadMJ.m._10/21
PH	2021_Pullman	180	TotalSolarRadMJ.m._11/02
PH	2021_Pullman	180	TotalSolarRadMJ.m._11/17
PH	2021_Pullman	180	TotalSolarRadMJ.m._12/15
PH	2021_Pullman	180	TotalSolarRadMJ.m._12/31
PH	2021_Pullman	180	TotalSolarRadMJ.m._01/06
PH	2021_Pullman	180	TotalSolarRadMJ.m._01/20
PH	2021_Pullman	180	TotalSolarRadMJ.m._01/24
PH	2021_Pullman	180	TotalSolarRadMJ.m._02/06
PH	2021_Pullman	180	TotalSolarRadMJ.m._02/26
PH	2021_Pullman	180	TotalSolarRadMJ.m._02/27
PH	2021_Pullman	180	TotalSolarRadMJ.m._03/07

PH	2021_Pullman	180	TotalSolarRadMJ.m._03/12
PH	2021_Pullman	180	TotalSolarRadMJ.m._04/24
PH	2021_Pullman	180	TotalSolarRadMJ.m._04/26
PH	2021_Pullman	180	TotalSolarRadMJ.m._04/28
PH	2021_Pullman	180	TotalSolarRadMJ.m._06/15
PH	2021_Pullman	180	ETo in_10/20
PH	2021_Pullman	180	ETo in_02/12
PH	2021_Pullman	180	ETo in_03/02
PH	2021_Pullman	180	ETo in_03/04
PH	2021_Pullman	180	ETr in_10/20
PH	2021_Pullman	180	ETr in_11/06
PH	2021_Pullman	180	ETr in_02/12
PH	2021_Pullman	180	ETr in_03/02
PH	2021_Pullman	180	ETr in_03/04
PH	2021_Ritzville	3	Min.F_06/12
PH	2021_Ritzville	3	Avg1.5m.DP.F_04/24
PH	2021_Ritzville	3	Avg1.5m.DP.F_05/13
PH	2021_Walla_Walla	122	Min.F_11/02
PH	2021_Walla_Walla	122	Min.F_11/03
PH	2021_Walla_Walla	122	Min.F_11/16
PH	2021_Walla_Walla	122	Min.F_01/07
PH	2021_Walla_Walla	122	Min.F_01/28
PH	2021_Walla_Walla	122	Min.F_01/31
PH	2021_Walla_Walla	122	Min.F_02/23
PH	2021_Walla_Walla	122	Min.F_06/12
PH	2021_Walla_Walla	122	Avg.F_10/28
PH	2021_Walla_Walla	122	Avg.F_11/02
PH	2021_Walla_Walla	122	Avg.F_11/18
PH	2021_Walla_Walla	122	Avg.F_11/19
PH	2021_Walla_Walla	122	Avg.F_12/21
PH	2021_Walla_Walla	122	Avg.F_01/03
PH	2021_Walla_Walla	122	Avg.F_01/07
PH	2021_Walla_Walla	122	Avg.F_01/08
PH	2021_Walla_Walla	122	Avg.F_01/28
PH	2021_Walla_Walla	122	Avg.F_01/29
PH	2021_Walla_Walla	122	Avg.F_01/30
PH	2021_Walla_Walla	122	Avg.F_01/31
PH	2021_Walla_Walla	122	Avg.F_02/25
PH	2021_Walla_Walla	122	Avg.F_02/26
PH	2021_Walla_Walla	122	Avg.F_03/06
PH	2021_Walla_Walla	122	Avg.F_03/11
PH	2021_Walla_Walla	122	Avg.F_04/27
PH	2021_Walla_Walla	122	Avg.F_04/28
PH	2021_Walla_Walla	122	Avg.F_05/02

PH	2021_Walla_Walla	122	Max.F_12/22
PH	2021_Walla_Walla	122	Max.F_01/03
PH	2021_Walla_Walla	122	Max.F_01/08
PH	2021_Walla_Walla	122	Max.F_02/21
PH	2021_Walla_Walla	122	Max.F_02/22
PH	2021_Walla_Walla	122	Max.F_02/25
PH	2021_Walla_Walla	122	Max.F_02/26
PH	2021_Walla_Walla	122	Max.F_03/05
PH	2021_Walla_Walla	122	Max.F_03/06
PH	2021_Walla_Walla	122	Max.F_03/10
PH	2021_Walla_Walla	122	Max.F_03/11
PH	2021_Walla_Walla	122	Max.F_04/09
PH	2021_Walla_Walla	122	Max.F_04/20
PH	2021_Walla_Walla	122	Max.F_04/27
PH	2021_Walla_Walla	122	Max.F_04/28
PH	2021_Walla_Walla	122	Max.F_05/02
PH	2021_Walla_Walla	122	Max.F_06/23
PH	2021_Walla_Walla	122	Avg1.5m.DP.F_10/28
PH	2021_Walla_Walla	122	Avg1.5m.DP.F_11/02
PH	2021_Walla_Walla	122	Avg1.5m.DP.F_11/17
PH	2021_Walla_Walla	122	Avg1.5m.DP.F_11/18
PH	2021_Walla_Walla	122	Avg1.5m.DP.F_12/05
PH	2021_Walla_Walla	122	Avg1.5m.DP.F_12/09
PH	2021_Walla_Walla	122	Avg1.5m.DP.F_01/08
PH	2021_Walla_Walla	122	Avg1.5m.DP.F_01/30
PH	2021_Walla_Walla	122	Avg1.5m.DP.F_01/31
PH	2021_Walla_Walla	122	Avg1.5m.DP.F_02/26
PH	2021_Walla_Walla	122	Avg1.5m.DP.F_03/06
PH	2021_Walla_Walla	122	Avg1.5m.DP.F_03/10
PH	2021_Walla_Walla	122	Avg1.5m.DP.F_04/24
PH	2021_Walla_Walla	122	Avg1.5m.DP.F_04/25
PH	2021_Walla_Walla	122	Avg1.5m.DP.F_05/13
PH	2021_Walla_Walla	122	Avg1.5m.DP.F_06/23
PH	2021_Walla_Walla	122	Avg1.5m.DP.F_06/24
PH	2021_Walla_Walla	122	Avg1.5m.DP.F_06/25
PH	2021_Walla_Walla	122	Avg1.5m.RH._10/19
PH	2021_Walla_Walla	122	Avg1.5m.RH._03/01
PH	2021_Walla_Walla	122	Avg1.5m.RH._03/23
PH	2021_Walla_Walla	122	Avg1.5m.RH._05/20
PH	2021_Walla_Walla	122	Min.F.1_11/03
PH	2021_Walla_Walla	122	Min.F.1_11/18
PH	2021_Walla_Walla	122	Min.F.1_11/19
PH	2021_Walla_Walla	122	Min.F.1_11/20
PH	2021_Walla_Walla	122	Min.F.1_01/04

PH	2021_Walla_Walla	122	Min.F.1_01/08
PH	2021_Walla_Walla	122	Min.F.1_01/09
PH	2021_Walla_Walla	122	Min.F.1_03/11
PH	2021_Walla_Walla	122	Min.F.1_03/12
PH	2021_Walla_Walla	122	Avg.F.1_11/03
PH	2021_Walla_Walla	122	Avg.F.1_11/18
PH	2021_Walla_Walla	122	Avg.F.1_11/19
PH	2021_Walla_Walla	122	Avg.F.1_11/20
PH	2021_Walla_Walla	122	Avg.F.1_12/23
PH	2021_Walla_Walla	122	Avg.F.1_01/02
PH	2021_Walla_Walla	122	Avg.F.1_01/03
PH	2021_Walla_Walla	122	Avg.F.1_01/04
PH	2021_Walla_Walla	122	Avg.F.1_01/07
PH	2021_Walla_Walla	122	Avg.F.1_01/08
PH	2021_Walla_Walla	122	Avg.F.1_01/09
PH	2021_Walla_Walla	122	Avg.F.1_03/11
PH	2021_Walla_Walla	122	TotalSolarRadMJ.m._10/19
PH	2021_Walla_Walla	122	TotalSolarRadMJ.m._10/21
PH	2021_Walla_Walla	122	TotalSolarRadMJ.m._11/30
PH	2021_Walla_Walla	122	TotalSolarRadMJ.m._12/15
PH	2021_Walla_Walla	122	TotalSolarRadMJ.m._12/31
PH	2021_Walla_Walla	122	TotalSolarRadMJ.m._01/06
PH	2021_Walla_Walla	122	TotalSolarRadMJ.m._01/10
PH	2021_Walla_Walla	122	TotalSolarRadMJ.m._01/20
PH	2021_Walla_Walla	122	TotalSolarRadMJ.m._02/06
PH	2021_Walla_Walla	122	TotalSolarRadMJ.m._02/07
PH	2021_Walla_Walla	122	TotalSolarRadMJ.m._02/19
PH	2021_Walla_Walla	122	TotalSolarRadMJ.m._04/01
PH	2021_Walla_Walla	122	TotalSolarRadMJ.m._04/22
PH	2021_Walla_Walla	122	TotalSolarRadMJ.m._05/19
PH	2021_Walla_Walla	122	TotalSolarRadMJ.m._05/20
PH	2021_Walla_Walla	122	TotalSolarRadMJ.m._06/24
PH	2021_Walla_Walla	122	TotalSolarRadMJ.m._07/01
PH	2021_Walla_Walla	122	ETоin_10/20
PH	2021_Walla_Walla	122	EToin_11/14
PH	2021_Walla_Walla	122	EToin_02/23
PH	2021_Walla_Walla	122	EToin_02/24
PH	2021_Walla_Walla	122	EToin_03/03
PH	2021_Walla_Walla	122	EToin_03/10
PH	2021_Walla_Walla	122	EToin_03/11
PH	2021_Walla_Walla	122	EToin_05/19
PH	2021_Walla_Walla	122	EToin_05/20
PH	2021_Walla_Walla	122	ETrin_10/20
PH	2021_Walla_Walla	122	ETrin_12/09

PH	2021_Walla_Walla	122	ETrin_02/23
PH	2021_Walla_Walla	122	ETrin_02/24
PH	2021_Walla_Walla	122	ETrin_03/02
PH	2021_Walla_Walla	122	ETrin_03/03
PH	2021_Walla_Walla	122	ETrin_03/04
PH	2021_Walla_Walla	122	ETrin_03/11
PH	2021_Walla_Walla	122	ETrin_05/19
PH	2022_Davenport	3	Min.F_06/12
PH	2022_Davenport	3	Avg1.5m.DP.F_04/24
PH	2022_Davenport	3	Avg1.5m.DP.F_05/13
PH	2022_Farmington	3	Min.F_06/12
PH	2022_Farmington	3	Avg1.5m.DP.F_04/24
PH	2022_Farmington	3	Avg1.5m.DP.F_05/13
PH	2022_Harrington	3	Min.F_06/12
PH	2022_Harrington	3	Avg1.5m.DP.F_04/24
PH	2022_Harrington	3	Avg1.5m.DP.F_05/13
PH	2022_Prescott	4	Min.F_06/12
PH	2022_Prescott	4	Max.F_01/03
PH	2022_Prescott	4	Avg1.5m.DP.F_04/24
PH	2022_Prescott	4	Avg1.5m.DP.F_05/13
PH	2022_Pullman	2	Min.F_06/12
PH	2022_Pullman	2	Avg1.5m.DP.F_05/13
PH	2022_Ritzville	3	Min.F_06/12
PH	2022_Ritzville	3	Avg1.5m.DP.F_04/24
PH	2022_Ritzville	3	Avg1.5m.DP.F_05/13
HD	2019_Kincaid	153	Min.F_11/27
HD	2019_Kincaid	153	Min.F_12/02
HD	2019_Kincaid	153	Min.F_12/23
HD	2019_Kincaid	153	Min.F_01/19
HD	2019_Kincaid	153	Min.F_03/23
HD	2019_Kincaid	153	Min.F_06/20
HD	2019_Kincaid	153	Min.F_06/22
HD	2019_Kincaid	153	Min.F_06/23
HD	2019_Kincaid	153	Min.F_06/25
HD	2019_Kincaid	153	Min.F_06/26
HD	2019_Kincaid	153	Min.F_06/29
HD	2019_Kincaid	153	Min.F_06/30
HD	2019_Kincaid	153	Avg.F_04/15
HD	2019_Kincaid	153	Avg.F_05/16
HD	2019_Kincaid	153	Avg.F_05/17
HD	2019_Kincaid	153	Avg.F_06/19
HD	2019_Kincaid	153	Avg.F_06/20
HD	2019_Kincaid	153	Avg.F_06/22
HD	2019_Kincaid	153	Avg.F_06/23

HD	2019_Kincaid	153	Avg.F_06/24
HD	2019_Kincaid	153	Avg.F_06/25
HD	2019_Kincaid	153	Avg.F_06/26
HD	2019_Kincaid	153	Avg.F_06/29
HD	2019_Kincaid	153	Avg.F_07/03
HD	2019_Kincaid	153	Avg.F_07/04
HD	2019_Kincaid	153	Avg.F_07/05
HD	2019_Kincaid	153	Avg.F_07/06
HD	2019_Kincaid	153	Max.F_04/06
HD	2019_Kincaid	153	Max.F_04/15
HD	2019_Kincaid	153	Max.F_04/16
HD	2019_Kincaid	153	Max.F_04/30
HD	2019_Kincaid	153	Max.F_05/16
HD	2019_Kincaid	153	Max.F_06/03
HD	2019_Kincaid	153	Max.F_06/19
HD	2019_Kincaid	153	Max.F_06/20
HD	2019_Kincaid	153	Max.F_06/22
HD	2019_Kincaid	153	Max.F_06/24
HD	2019_Kincaid	153	Max.F_06/25
HD	2019_Kincaid	153	Max.F_06/26
HD	2019_Kincaid	153	Max.F_06/29
HD	2019_Kincaid	153	Max.F_07/03
HD	2019_Kincaid	153	Max.F_07/04
HD	2019_Kincaid	153	Max.F_07/06
HD	2019_Kincaid	153	Max.F_07/07
HD	2019_Kincaid	153	Avg1.5m.DP.F_10/24
HD	2019_Kincaid	153	Avg1.5m.DP.F_01/04
HD	2019_Kincaid	153	Avg1.5m.DP.F_01/12
HD	2019_Kincaid	153	Avg1.5m.DP.F_05/27
HD	2019_Kincaid	153	Avg1.5m.DP.F_06/06
HD	2019_Kincaid	153	Avg1.5m.RH._11/16
HD	2019_Kincaid	153	Avg1.5m.RH._11/26
HD	2019_Kincaid	153	Avg1.5m.RH._02/27
HD	2019_Kincaid	153	Avg1.5m.RH._04/10
HD	2019_Kincaid	153	Avg1.5m.RH._04/18
HD	2019_Kincaid	153	Avg1.5m.RH._04/19
HD	2019_Kincaid	153	Avg1.5m.RH._04/21
HD	2019_Kincaid	153	Avg1.5m.RH._05/15
HD	2019_Kincaid	153	Avg1.5m.RH._05/16
HD	2019_Kincaid	153	Avg1.5m.RH._05/21
HD	2019_Kincaid	153	Avg1.5m.RH._05/27
HD	2019_Kincaid	153	Avg1.5m.RH._05/29
HD	2019_Kincaid	153	Avg1.5m.RH._06/02
HD	2019_Kincaid	153	Avg1.5m.RH._06/06

HD	2019_Kincaid	153	Avg1.5m.RH._06/08
HD	2019_Kincaid	153	Avg1.5m.RH._06/20
HD	2019_Kincaid	153	Avg1.5m.RH._06/21
HD	2019_Kincaid	153	Avg1.5m.RH._06/22
HD	2019_Kincaid	153	Avg1.5m.RH._06/25
HD	2019_Kincaid	153	Avg1.5m.RH._06/27
HD	2019_Kincaid	153	Avg1.5m.RH._07/02
HD	2019_Kincaid	153	Avg1.5m.RH._07/03
HD	2019_Kincaid	153	Avg1.5m.RH._07/04
HD	2019_Kincaid	153	Avg1.5m.RH._07/05
HD	2019_Kincaid	153	Avg1.5m.RH._07/06
HD	2019_Kincaid	153	Avg1.5m.RH._07/07
HD	2019_Kincaid	153	Avg1.5m.RH._07/08
HD	2019_Kincaid	153	Min.F.1_06/22
HD	2019_Kincaid	153	Min.F.1_06/23
HD	2019_Kincaid	153	Min.F.1_06/24
HD	2019_Kincaid	153	Min.F.1_06/25
HD	2019_Kincaid	153	Min.F.1_06/26
HD	2019_Kincaid	153	Min.F.1_06/27
HD	2019_Kincaid	153	Min.F.1_06/28
HD	2019_Kincaid	153	Min.F.1_06/29
HD	2019_Kincaid	153	Min.F.1_06/30
HD	2019_Kincaid	153	Avg.F.1_06/21
HD	2019_Kincaid	153	Avg.F.1_06/22
HD	2019_Kincaid	153	Avg.F.1_06/23
HD	2019_Kincaid	153	Avg.F.1_06/24
HD	2019_Kincaid	153	Avg.F.1_06/25
HD	2019_Kincaid	153	Avg.F.1_06/26
HD	2019_Kincaid	153	Avg.F.1_06/27
HD	2019_Kincaid	153	Avg.F.1_06/28
HD	2019_Kincaid	153	TotPrecin_01/01
HD	2019_Kincaid	153	TotPrecin_06/05
HD	2019_Kincaid	153	TotalSolarRadMJ.m._10/26
HD	2019_Kincaid	153	TotalSolarRadMJ.m._11/08
HD	2019_Kincaid	153	TotalSolarRadMJ.m._03/05
HD	2019_Kincaid	153	TotalSolarRadMJ.m._05/26
HD	2019_Kincaid	153	EToin_04/12
HD	2019_Kincaid	153	EToin_04/13
HD	2019_Kincaid	153	EToin_04/14
HD	2019_Kincaid	153	EToin_04/15
HD	2019_Kincaid	153	EToin_04/16
HD	2019_Kincaid	153	EToin_04/18
HD	2019_Kincaid	153	EToin_05/15
HD	2019_Kincaid	153	EToin_05/16

HD	2019_Kincaid	153	EToin_05/26
HD	2019_Kincaid	153	EToin_06/20
HD	2019_Kincaid	153	EToin_06/21
HD	2019_Kincaid	153	EToin_06/22
HD	2019_Kincaid	153	EToin_06/23
HD	2019_Kincaid	153	EToin_06/24
HD	2019_Kincaid	153	EToin_06/25
HD	2019_Kincaid	153	EToin_06/26
HD	2019_Kincaid	153	EToin_06/29
HD	2019_Kincaid	153	EToin_06/30
HD	2019_Kincaid	153	EToin_07/02
HD	2019_Kincaid	153	EToin_07/03
HD	2019_Kincaid	153	EToin_07/04
HD	2019_Kincaid	153	EToin_07/05
HD	2019_Kincaid	153	EToin_07/06
HD	2019_Kincaid	153	EToin_07/07
HD	2019_Kincaid	153	EToin_07/08
HD	2019_Kincaid	153	ETrin_12/12
HD	2019_Kincaid	153	ETrin_04/12
HD	2019_Kincaid	153	ETrin_04/14
HD	2019_Kincaid	153	ETrin_04/15
HD	2019_Kincaid	153	ETrin_04/16
HD	2019_Kincaid	153	ETrin_04/18
HD	2019_Kincaid	153	ETrin_04/20
HD	2019_Kincaid	153	ETrin_05/15
HD	2019_Kincaid	153	ETrin_05/16
HD	2019_Kincaid	153	ETrin_05/26
HD	2019_Kincaid	153	ETrin_05/27
HD	2019_Kincaid	153	ETrin_06/20
HD	2019_Kincaid	153	ETrin_06/21
HD	2019_Kincaid	153	ETrin_06/22
HD	2019_Kincaid	153	ETrin_06/23
HD	2019_Kincaid	153	ETrin_06/24
HD	2019_Kincaid	153	ETrin_06/25
HD	2019_Kincaid	153	ETrin_06/26
HD	2019_Kincaid	153	ETrin_06/27
HD	2019_Kincaid	153	ETrin_06/29
HD	2019_Kincaid	153	ETrin_06/30
HD	2019_Kincaid	153	ETrin_07/01
HD	2019_Kincaid	153	ETrin_07/02
HD	2019_Kincaid	153	ETrin_07/03
HD	2019_Kincaid	153	ETrin_07/04
HD	2019_Kincaid	153	ETrin_07/05
HD	2019_Kincaid	153	ETrin_07/06

HD	2019_Kincaid	153	ETrin_07/07
HD	2019_Kincaid	153	ETrin_07/08
HD	2019_Pullman	102	Min.F_11/27
HD	2019_Pullman	102	Min.F_12/02
HD	2019_Pullman	102	Min.F_01/19
HD	2019_Pullman	102	Min.F_03/23
HD	2019_Pullman	102	Min.F_06/23
HD	2019_Pullman	102	Min.F_06/26
HD	2019_Pullman	102	Avg.F_04/15
HD	2019_Pullman	102	Avg.F_05/16
HD	2019_Pullman	102	Avg.F_05/17
HD	2019_Pullman	102	Avg.F_06/19
HD	2019_Pullman	102	Avg.F_06/23
HD	2019_Pullman	102	Avg.F_06/25
HD	2019_Pullman	102	Avg.F_06/26
HD	2019_Pullman	102	Avg.F_06/29
HD	2019_Pullman	102	Avg.F_07/03
HD	2019_Pullman	102	Avg.F_07/04
HD	2019_Pullman	102	Avg.F_07/05
HD	2019_Pullman	102	Avg.F_07/06
HD	2019_Pullman	102	Max.F_04/15
HD	2019_Pullman	102	Max.F_04/16
HD	2019_Pullman	102	Max.F_04/30
HD	2019_Pullman	102	Max.F_05/16
HD	2019_Pullman	102	Max.F_06/03
HD	2019_Pullman	102	Max.F_06/22
HD	2019_Pullman	102	Max.F_06/24
HD	2019_Pullman	102	Max.F_06/25
HD	2019_Pullman	102	Max.F_06/29
HD	2019_Pullman	102	Max.F_07/03
HD	2019_Pullman	102	Max.F_07/04
HD	2019_Pullman	102	Max.F_07/06
HD	2019_Pullman	102	Avg1.5m.DP.F_10/24
HD	2019_Pullman	102	Avg1.5m.DP.F_01/12
HD	2019_Pullman	102	Avg1.5m.DP.F_05/27
HD	2019_Pullman	102	Avg1.5m.DP.F_06/06
HD	2019_Pullman	102	Avg1.5m.RH._11/26
HD	2019_Pullman	102	Avg1.5m.RH._04/10
HD	2019_Pullman	102	Avg1.5m.RH._04/19
HD	2019_Pullman	102	Avg1.5m.RH._04/21
HD	2019_Pullman	102	Avg1.5m.RH._05/15
HD	2019_Pullman	102	Avg1.5m.RH._05/21
HD	2019_Pullman	102	Avg1.5m.RH._05/27
HD	2019_Pullman	102	Avg1.5m.RH._05/29

HD	2019_Pullman	102	Avg1.5m.RH._06/02
HD	2019_Pullman	102	Avg1.5m.RH._06/06
HD	2019_Pullman	102	Avg1.5m.RH._06/08
HD	2019_Pullman	102	Avg1.5m.RH._06/21
HD	2019_Pullman	102	Avg1.5m.RH._06/22
HD	2019_Pullman	102	Avg1.5m.RH._06/25
HD	2019_Pullman	102	Avg1.5m.RH._07/02
HD	2019_Pullman	102	Avg1.5m.RH._07/03
HD	2019_Pullman	102	Avg1.5m.RH._07/04
HD	2019_Pullman	102	Avg1.5m.RH._07/05
HD	2019_Pullman	102	Avg1.5m.RH._07/06
HD	2019_Pullman	102	Avg1.5m.RH._07/07
HD	2019_Pullman	102	Avg1.5m.RH._07/08
HD	2019_Pullman	102	Min.F.1_06/26
HD	2019_Pullman	102	Avg.F.1_06/26
HD	2019_Pullman	102	TotPrecin_01/01
HD	2019_Pullman	102	TotPrecin_06/05
HD	2019_Pullman	102	TotalSolarRadMJ.m._11/08
HD	2019_Pullman	102	EToin_04/14
HD	2019_Pullman	102	EToin_04/16
HD	2019_Pullman	102	EToin_04/18
HD	2019_Pullman	102	EToin_05/16
HD	2019_Pullman	102	EToin_06/20
HD	2019_Pullman	102	EToin_06/22
HD	2019_Pullman	102	EToin_06/23
HD	2019_Pullman	102	EToin_06/24
HD	2019_Pullman	102	EToin_06/25
HD	2019_Pullman	102	EToin_06/29
HD	2019_Pullman	102	EToin_06/30
HD	2019_Pullman	102	EToin_07/02
HD	2019_Pullman	102	EToin_07/03
HD	2019_Pullman	102	EToin_07/04
HD	2019_Pullman	102	EToin_07/05
HD	2019_Pullman	102	EToin_07/06
HD	2019_Pullman	102	EToin_07/07
HD	2019_Pullman	102	ETrin_04/14
HD	2019_Pullman	102	ETrin_04/15
HD	2019_Pullman	102	ETrin_04/16
HD	2019_Pullman	102	ETrin_04/18
HD	2019_Pullman	102	ETrin_05/15
HD	2019_Pullman	102	ETrin_05/16
HD	2019_Pullman	102	ETrin_05/26
HD	2019_Pullman	102	ETrin_05/27
HD	2019_Pullman	102	ETrin_06/20

HD	2019_Pullman	102	ETrin_06/21
HD	2019_Pullman	102	ETrin_06/22
HD	2019_Pullman	102	ETrin_06/23
HD	2019_Pullman	102	ETrin_06/24
HD	2019_Pullman	102	ETrin_06/25
HD	2019_Pullman	102	ETrin_06/26
HD	2019_Pullman	102	ETrin_06/29
HD	2019_Pullman	102	ETrin_06/30
HD	2019_Pullman	102	ETrin_07/01
HD	2019_Pullman	102	ETrin_07/02
HD	2019_Pullman	102	ETrin_07/03
HD	2019_Pullman	102	ETrin_07/04
HD	2019_Pullman	102	ETrin_07/05
HD	2019_Pullman	102	ETrin_07/06
HD	2019_Pullman	102	ETrin_07/07
HD	2019_Pullman	102	ETrin_07/08
HD	2020_Farmington	134	Min.F_11/27
HD	2020_Farmington	134	Min.F_12/02
HD	2020_Farmington	134	Min.F_12/23
HD	2020_Farmington	134	Min.F_01/19
HD	2020_Farmington	134	Min.F_03/23
HD	2020_Farmington	134	Min.F_06/22
HD	2020_Farmington	134	Min.F_06/23
HD	2020_Farmington	134	Min.F_06/26
HD	2020_Farmington	134	Min.F_06/30
HD	2020_Farmington	134	Avg.F_11/27
HD	2020_Farmington	134	Avg.F_04/15
HD	2020_Farmington	134	Avg.F_05/16
HD	2020_Farmington	134	Avg.F_05/17
HD	2020_Farmington	134	Avg.F_06/19
HD	2020_Farmington	134	Avg.F_06/20
HD	2020_Farmington	134	Avg.F_06/22
HD	2020_Farmington	134	Avg.F_06/23
HD	2020_Farmington	134	Avg.F_06/24
HD	2020_Farmington	134	Avg.F_06/25
HD	2020_Farmington	134	Avg.F_06/26
HD	2020_Farmington	134	Avg.F_06/29
HD	2020_Farmington	134	Avg.F_07/03
HD	2020_Farmington	134	Avg.F_07/04
HD	2020_Farmington	134	Avg.F_07/06
HD	2020_Farmington	134	Max.F_04/06
HD	2020_Farmington	134	Max.F_04/14
HD	2020_Farmington	134	Max.F_04/15
HD	2020_Farmington	134	Max.F_04/16

HD	2020_Farmington	134	Max.F_04/17
HD	2020_Farmington	134	Max.F_04/30
HD	2020_Farmington	134	Max.F_05/16
HD	2020_Farmington	134	Max.F_06/03
HD	2020_Farmington	134	Max.F_06/19
HD	2020_Farmington	134	Max.F_06/20
HD	2020_Farmington	134	Max.F_06/22
HD	2020_Farmington	134	Max.F_06/24
HD	2020_Farmington	134	Max.F_06/25
HD	2020_Farmington	134	Max.F_06/29
HD	2020_Farmington	134	Max.F_07/03
HD	2020_Farmington	134	Max.F_07/06
HD	2020_Farmington	134	Max.F_07/07
HD	2020_Farmington	134	Avg1.5m.DP.F_10/24
HD	2020_Farmington	134	Avg1.5m.DP.F_12/02
HD	2020_Farmington	134	Avg1.5m.DP.F_01/12
HD	2020_Farmington	134	Avg1.5m.DP.F_05/27
HD	2020_Farmington	134	Avg1.5m.DP.F_06/06
HD	2020_Farmington	134	Avg1.5m.RH._11/16
HD	2020_Farmington	134	Avg1.5m.RH._11/26
HD	2020_Farmington	134	Avg1.5m.RH._02/27
HD	2020_Farmington	134	Avg1.5m.RH._04/10
HD	2020_Farmington	134	Avg1.5m.RH._04/19
HD	2020_Farmington	134	Avg1.5m.RH._04/21
HD	2020_Farmington	134	Avg1.5m.RH._05/15
HD	2020_Farmington	134	Avg1.5m.RH._05/21
HD	2020_Farmington	134	Avg1.5m.RH._05/27
HD	2020_Farmington	134	Avg1.5m.RH._06/02
HD	2020_Farmington	134	Avg1.5m.RH._06/06
HD	2020_Farmington	134	Avg1.5m.RH._06/08
HD	2020_Farmington	134	Avg1.5m.RH._06/21
HD	2020_Farmington	134	Avg1.5m.RH._06/22
HD	2020_Farmington	134	Avg1.5m.RH._06/25
HD	2020_Farmington	134	Avg1.5m.RH._07/02
HD	2020_Farmington	134	Avg1.5m.RH._07/03
HD	2020_Farmington	134	Avg1.5m.RH._07/04
HD	2020_Farmington	134	Avg1.5m.RH._07/05
HD	2020_Farmington	134	Avg1.5m.RH._07/06
HD	2020_Farmington	134	Avg1.5m.RH._07/07
HD	2020_Farmington	134	Avg1.5m.RH._07/08
HD	2020_Farmington	134	Min.F.1_06/22
HD	2020_Farmington	134	Min.F.1_06/23
HD	2020_Farmington	134	Min.F.1_06/24
HD	2020_Farmington	134	Min.F.1_06/25

HD	2020_Farmington	134	Min.F.1_06/26
HD	2020_Farmington	134	Min.F.1_06/27
HD	2020_Farmington	134	Min.F.1_06/30
HD	2020_Farmington	134	Avg.F.1_06/21
HD	2020_Farmington	134	Avg.F.1_06/22
HD	2020_Farmington	134	Avg.F.1_06/23
HD	2020_Farmington	134	Avg.F.1_06/24
HD	2020_Farmington	134	Avg.F.1_06/25
HD	2020_Farmington	134	Avg.F.1_06/26
HD	2020_Farmington	134	Avg.F.1_06/27
HD	2020_Farmington	134	TotPrecin_01/01
HD	2020_Farmington	134	TotPrecin_06/05
HD	2020_Farmington	134	TotalSolarRadMJ.m._11/08
HD	2020_Farmington	134	TotalSolarRadMJ.m._02/09
HD	2020_Farmington	134	TotalSolarRadMJ.m._03/05
HD	2020_Farmington	134	ETоin_04/12
HD	2020_Farmington	134	ETоin_04/14
HD	2020_Farmington	134	ETоin_04/15
HD	2020_Farmington	134	ETоin_04/16
HD	2020_Farmington	134	ETоin_04/18
HD	2020_Farmington	134	ETоin_05/15
HD	2020_Farmington	134	ETоin_05/16
HD	2020_Farmington	134	ETоin_06/20
HD	2020_Farmington	134	ETоin_06/22
HD	2020_Farmington	134	ETоin_06/23
HD	2020_Farmington	134	ETоin_06/24
HD	2020_Farmington	134	ETоin_06/25
HD	2020_Farmington	134	ETоin_06/26
HD	2020_Farmington	134	ETоin_06/29
HD	2020_Farmington	134	ETоin_06/30
HD	2020_Farmington	134	ETоin_07/02
HD	2020_Farmington	134	ETоin_07/03
HD	2020_Farmington	134	ETоin_07/04
HD	2020_Farmington	134	ETоin_07/05
HD	2020_Farmington	134	ETоin_07/06
HD	2020_Farmington	134	ETоin_07/07
HD	2020_Farmington	134	ETоin_07/08
HD	2020_Farmington	134	ETrin_12/12
HD	2020_Farmington	134	ETrin_04/14
HD	2020_Farmington	134	ETrin_04/15
HD	2020_Farmington	134	ETrin_04/16
HD	2020_Farmington	134	ETrin_04/18
HD	2020_Farmington	134	ETrin_05/15
HD	2020_Farmington	134	ETrin_05/16

HD	2020_Farmington	134	ETrin_05/26
HD	2020_Farmington	134	ETrin_05/27
HD	2020_Farmington	134	ETrin_06/21
HD	2020_Farmington	134	ETrin_06/22
HD	2020_Farmington	134	ETrin_06/23
HD	2020_Farmington	134	ETrin_06/24
HD	2020_Farmington	134	ETrin_06/25
HD	2020_Farmington	134	ETrin_06/26
HD	2020_Farmington	134	ETrin_06/29
HD	2020_Farmington	134	ETrin_06/30
HD	2020_Farmington	134	ETrin_07/01
HD	2020_Farmington	134	ETrin_07/02
HD	2020_Farmington	134	ETrin_07/03
HD	2020_Farmington	134	ETrin_07/04
HD	2020_Farmington	134	ETrin_07/05
HD	2020_Farmington	134	ETrin_07/06
HD	2020_Farmington	134	ETrin_07/07
HD	2020_Farmington	134	ETrin_07/08
HD	2020_Harrington	151	Min.F_10/24
HD	2020_Harrington	151	Min.F_11/27
HD	2020_Harrington	151	Min.F_12/02
HD	2020_Harrington	151	Min.F_12/23
HD	2020_Harrington	151	Min.F_01/19
HD	2020_Harrington	151	Min.F_03/23
HD	2020_Harrington	151	Min.F_06/22
HD	2020_Harrington	151	Min.F_06/23
HD	2020_Harrington	151	Min.F_06/26
HD	2020_Harrington	151	Min.F_06/29
HD	2020_Harrington	151	Min.F_06/30
HD	2020_Harrington	151	Min.F_07/01
HD	2020_Harrington	151	Min.F_07/02
HD	2020_Harrington	151	Avg.F_04/15
HD	2020_Harrington	151	Avg.F_05/16
HD	2020_Harrington	151	Avg.F_05/17
HD	2020_Harrington	151	Avg.F_06/19
HD	2020_Harrington	151	Avg.F_06/22
HD	2020_Harrington	151	Avg.F_06/23
HD	2020_Harrington	151	Avg.F_06/25
HD	2020_Harrington	151	Avg.F_06/26
HD	2020_Harrington	151	Avg.F_06/29
HD	2020_Harrington	151	Avg.F_06/30
HD	2020_Harrington	151	Avg.F_07/03
HD	2020_Harrington	151	Avg.F_07/04
HD	2020_Harrington	151	Avg.F_07/05

HD	2020_Harrington	151	Avg.F_07/06
HD	2020_Harrington	151	Avg.F_07/07
HD	2020_Harrington	151	Max.F_04/06
HD	2020_Harrington	151	Max.F_04/15
HD	2020_Harrington	151	Max.F_04/16
HD	2020_Harrington	151	Max.F_04/17
HD	2020_Harrington	151	Max.F_04/30
HD	2020_Harrington	151	Max.F_05/16
HD	2020_Harrington	151	Max.F_06/03
HD	2020_Harrington	151	Max.F_06/19
HD	2020_Harrington	151	Max.F_06/20
HD	2020_Harrington	151	Max.F_06/22
HD	2020_Harrington	151	Max.F_06/24
HD	2020_Harrington	151	Max.F_06/25
HD	2020_Harrington	151	Max.F_06/26
HD	2020_Harrington	151	Max.F_06/29
HD	2020_Harrington	151	Max.F_07/03
HD	2020_Harrington	151	Max.F_07/04
HD	2020_Harrington	151	Max.F_07/06
HD	2020_Harrington	151	Max.F_07/07
HD	2020_Harrington	151	Avg1.5m.DP.F_10/24
HD	2020_Harrington	151	Avg1.5m.DP.F_01/04
HD	2020_Harrington	151	Avg1.5m.DP.F_01/12
HD	2020_Harrington	151	Avg1.5m.DP.F_02/12
HD	2020_Harrington	151	Avg1.5m.DP.F_03/29
HD	2020_Harrington	151	Avg1.5m.DP.F_05/27
HD	2020_Harrington	151	Avg1.5m.DP.F_06/06
HD	2020_Harrington	151	Avg1.5m.DP.F_06/14
HD	2020_Harrington	151	Avg1.5m.DP.F_06/17
HD	2020_Harrington	151	Avg1.5m.RH._11/26
HD	2020_Harrington	151	Avg1.5m.RH._02/27
HD	2020_Harrington	151	Avg1.5m.RH._04/10
HD	2020_Harrington	151	Avg1.5m.RH._04/18
HD	2020_Harrington	151	Avg1.5m.RH._04/19
HD	2020_Harrington	151	Avg1.5m.RH._04/21
HD	2020_Harrington	151	Avg1.5m.RH._05/15
HD	2020_Harrington	151	Avg1.5m.RH._05/16
HD	2020_Harrington	151	Avg1.5m.RH._05/21
HD	2020_Harrington	151	Avg1.5m.RH._05/27
HD	2020_Harrington	151	Avg1.5m.RH._06/02
HD	2020_Harrington	151	Avg1.5m.RH._06/06
HD	2020_Harrington	151	Avg1.5m.RH._06/07
HD	2020_Harrington	151	Avg1.5m.RH._06/08
HD	2020_Harrington	151	Avg1.5m.RH._06/21

HD	2020_Harrington	151	Avg1.5m.RH._06/22
HD	2020_Harrington	151	Avg1.5m.RH._06/25
HD	2020_Harrington	151	Avg1.5m.RH._06/30
HD	2020_Harrington	151	Avg1.5m.RH._07/01
HD	2020_Harrington	151	Avg1.5m.RH._07/02
HD	2020_Harrington	151	Avg1.5m.RH._07/03
HD	2020_Harrington	151	Avg1.5m.RH._07/04
HD	2020_Harrington	151	Avg1.5m.RH._07/05
HD	2020_Harrington	151	Avg1.5m.RH._07/06
HD	2020_Harrington	151	Avg1.5m.RH._07/07
HD	2020_Harrington	151	Avg1.5m.RH._07/08
HD	2020_Harrington	151	Min.F.1_06/22
HD	2020_Harrington	151	Min.F.1_06/23
HD	2020_Harrington	151	Min.F.1_06/24
HD	2020_Harrington	151	Min.F.1_06/26
HD	2020_Harrington	151	Min.F.1_06/27
HD	2020_Harrington	151	Min.F.1_06/29
HD	2020_Harrington	151	Min.F.1_06/30
HD	2020_Harrington	151	Min.F.1_07/04
HD	2020_Harrington	151	Min.F.1_07/05
HD	2020_Harrington	151	Avg.F.1_06/21
HD	2020_Harrington	151	Avg.F.1_06/22
HD	2020_Harrington	151	Avg.F.1_06/23
HD	2020_Harrington	151	Avg.F.1_06/24
HD	2020_Harrington	151	Avg.F.1_06/25
HD	2020_Harrington	151	Avg.F.1_06/26
HD	2020_Harrington	151	Avg.F.1_06/27
HD	2020_Harrington	151	Avg.F.1_06/28
HD	2020_Harrington	151	Avg.F.1_06/29
HD	2020_Harrington	151	Avg.F.1_06/30
HD	2020_Harrington	151	Avg.F.1_07/05
HD	2020_Harrington	151	TotPrecin_01/01
HD	2020_Harrington	151	TotPrecin_06/05
HD	2020_Harrington	151	TotalSolarRadMJ.m._11/08
HD	2020_Harrington	151	EToin_12/28
HD	2020_Harrington	151	EToin_04/12
HD	2020_Harrington	151	EToin_04/14
HD	2020_Harrington	151	EToin_04/15
HD	2020_Harrington	151	EToin_04/16
HD	2020_Harrington	151	EToin_04/18
HD	2020_Harrington	151	EToin_05/15
HD	2020_Harrington	151	EToin_05/16
HD	2020_Harrington	151	EToin_06/20
HD	2020_Harrington	151	EToin_06/22

HD	2020_Harrington	151	EToin_06/23
HD	2020_Harrington	151	EToin_06/24
HD	2020_Harrington	151	EToin_06/25
HD	2020_Harrington	151	EToin_06/29
HD	2020_Harrington	151	EToin_06/30
HD	2020_Harrington	151	EToin_07/02
HD	2020_Harrington	151	EToin_07/03
HD	2020_Harrington	151	EToin_07/04
HD	2020_Harrington	151	EToin_07/05
HD	2020_Harrington	151	EToin_07/06
HD	2020_Harrington	151	EToin_07/07
HD	2020_Harrington	151	EToin_07/08
HD	2020_Harrington	151	ETrin_04/14
HD	2020_Harrington	151	ETrin_04/15
HD	2020_Harrington	151	ETrin_04/16
HD	2020_Harrington	151	ETrin_04/18
HD	2020_Harrington	151	ETrin_05/15
HD	2020_Harrington	151	ETrin_05/16
HD	2020_Harrington	151	ETrin_05/26
HD	2020_Harrington	151	ETrin_05/27
HD	2020_Harrington	151	ETrin_06/20
HD	2020_Harrington	151	ETrin_06/21
HD	2020_Harrington	151	ETrin_06/22
HD	2020_Harrington	151	ETrin_06/23
HD	2020_Harrington	151	ETrin_06/24
HD	2020_Harrington	151	ETrin_06/25
HD	2020_Harrington	151	ETrin_06/26
HD	2020_Harrington	151	ETrin_06/29
HD	2020_Harrington	151	ETrin_06/30
HD	2020_Harrington	151	ETrin_07/01
HD	2020_Harrington	151	ETrin_07/02
HD	2020_Harrington	151	ETrin_07/03
HD	2020_Harrington	151	ETrin_07/04
HD	2020_Harrington	151	ETrin_07/05
HD	2020_Harrington	151	ETrin_07/06
HD	2020_Harrington	151	ETrin_07/07
HD	2020_Harrington	151	ETrin_07/08
HD	2020_Kincaid	216	Min.F_10/24
HD	2020_Kincaid	216	Min.F_10/27
HD	2020_Kincaid	216	Min.F_11/03
HD	2020_Kincaid	216	Min.F_11/27
HD	2020_Kincaid	216	Min.F_12/02
HD	2020_Kincaid	216	Min.F_01/18
HD	2020_Kincaid	216	Min.F_01/19

HD	2020_Kincaid	216	Min.F_01/30
HD	2020_Kincaid	216	Min.F_02/22
HD	2020_Kincaid	216	Min.F_02/23
HD	2020_Kincaid	216	Min.F_03/23
HD	2020_Kincaid	216	Min.F_06/20
HD	2020_Kincaid	216	Min.F_06/23
HD	2020_Kincaid	216	Min.F_06/24
HD	2020_Kincaid	216	Min.F_06/25
HD	2020_Kincaid	216	Min.F_06/26
HD	2020_Kincaid	216	Min.F_06/30
HD	2020_Kincaid	216	Avg.F_10/27
HD	2020_Kincaid	216	Avg.F_11/27
HD	2020_Kincaid	216	Avg.F_02/22
HD	2020_Kincaid	216	Avg.F_02/25
HD	2020_Kincaid	216	Avg.F_04/15
HD	2020_Kincaid	216	Avg.F_04/29
HD	2020_Kincaid	216	Avg.F_05/16
HD	2020_Kincaid	216	Avg.F_05/17
HD	2020_Kincaid	216	Avg.F_06/19
HD	2020_Kincaid	216	Avg.F_06/20
HD	2020_Kincaid	216	Avg.F_06/22
HD	2020_Kincaid	216	Avg.F_06/23
HD	2020_Kincaid	216	Avg.F_06/24
HD	2020_Kincaid	216	Avg.F_06/25
HD	2020_Kincaid	216	Avg.F_06/26
HD	2020_Kincaid	216	Avg.F_06/29
HD	2020_Kincaid	216	Avg.F_07/03
HD	2020_Kincaid	216	Avg.F_07/04
HD	2020_Kincaid	216	Avg.F_07/05
HD	2020_Kincaid	216	Avg.F_07/06
HD	2020_Kincaid	216	Max.F_02/22
HD	2020_Kincaid	216	Max.F_02/24
HD	2020_Kincaid	216	Max.F_02/25
HD	2020_Kincaid	216	Max.F_04/06
HD	2020_Kincaid	216	Max.F_04/14
HD	2020_Kincaid	216	Max.F_04/15
HD	2020_Kincaid	216	Max.F_04/16
HD	2020_Kincaid	216	Max.F_04/17
HD	2020_Kincaid	216	Max.F_04/21
HD	2020_Kincaid	216	Max.F_04/27
HD	2020_Kincaid	216	Max.F_04/28
HD	2020_Kincaid	216	Max.F_04/29
HD	2020_Kincaid	216	Max.F_04/30
HD	2020_Kincaid	216	Max.F_05/01

HD	2020_Kincaid	216	Max.F_05/16
HD	2020_Kincaid	216	Max.F_05/17
HD	2020_Kincaid	216	Max.F_06/03
HD	2020_Kincaid	216	Max.F_06/19
HD	2020_Kincaid	216	Max.F_06/20
HD	2020_Kincaid	216	Max.F_06/22
HD	2020_Kincaid	216	Max.F_06/23
HD	2020_Kincaid	216	Max.F_06/24
HD	2020_Kincaid	216	Max.F_06/25
HD	2020_Kincaid	216	Max.F_06/26
HD	2020_Kincaid	216	Max.F_06/29
HD	2020_Kincaid	216	Max.F_07/03
HD	2020_Kincaid	216	Max.F_07/06
HD	2020_Kincaid	216	Max.F_07/07
HD	2020_Kincaid	216	Avg1.5m.DP.F_10/24
HD	2020_Kincaid	216	Avg1.5m.DP.F_10/27
HD	2020_Kincaid	216	Avg1.5m.DP.F_11/17
HD	2020_Kincaid	216	Avg1.5m.DP.F_12/02
HD	2020_Kincaid	216	Avg1.5m.DP.F_01/02
HD	2020_Kincaid	216	Avg1.5m.DP.F_01/03
HD	2020_Kincaid	216	Avg1.5m.DP.F_01/04
HD	2020_Kincaid	216	Avg1.5m.DP.F_01/06
HD	2020_Kincaid	216	Avg1.5m.DP.F_01/12
HD	2020_Kincaid	216	Avg1.5m.DP.F_01/30
HD	2020_Kincaid	216	Avg1.5m.DP.F_02/22
HD	2020_Kincaid	216	Avg1.5m.DP.F_03/23
HD	2020_Kincaid	216	Avg1.5m.DP.F_05/27
HD	2020_Kincaid	216	Avg1.5m.DP.F_06/06
HD	2020_Kincaid	216	Avg1.5m.DP.F_06/16
HD	2020_Kincaid	216	Avg1.5m.DP.F_06/17
HD	2020_Kincaid	216	Avg1.5m.RH._10/30
HD	2020_Kincaid	216	Avg1.5m.RH._11/16
HD	2020_Kincaid	216	Avg1.5m.RH._11/26
HD	2020_Kincaid	216	Avg1.5m.RH._02/27
HD	2020_Kincaid	216	Avg1.5m.RH._03/05
HD	2020_Kincaid	216	Avg1.5m.RH._03/23
HD	2020_Kincaid	216	Avg1.5m.RH._04/10
HD	2020_Kincaid	216	Avg1.5m.RH._04/11
HD	2020_Kincaid	216	Avg1.5m.RH._04/12
HD	2020_Kincaid	216	Avg1.5m.RH._04/14
HD	2020_Kincaid	216	Avg1.5m.RH._04/15
HD	2020_Kincaid	216	Avg1.5m.RH._04/16
HD	2020_Kincaid	216	Avg1.5m.RH._04/17
HD	2020_Kincaid	216	Avg1.5m.RH._04/18

HD	2020_Kincaid	216	Avg1.5m.RH._04/19
HD	2020_Kincaid	216	Avg1.5m.RH._04/21
HD	2020_Kincaid	216	Avg1.5m.RH._05/15
HD	2020_Kincaid	216	Avg1.5m.RH._05/16
HD	2020_Kincaid	216	Avg1.5m.RH._05/21
HD	2020_Kincaid	216	Avg1.5m.RH._05/27
HD	2020_Kincaid	216	Avg1.5m.RH._06/01
HD	2020_Kincaid	216	Avg1.5m.RH._06/02
HD	2020_Kincaid	216	Avg1.5m.RH._06/06
HD	2020_Kincaid	216	Avg1.5m.RH._06/08
HD	2020_Kincaid	216	Avg1.5m.RH._06/21
HD	2020_Kincaid	216	Avg1.5m.RH._06/22
HD	2020_Kincaid	216	Avg1.5m.RH._06/25
HD	2020_Kincaid	216	Avg1.5m.RH._06/29
HD	2020_Kincaid	216	Avg1.5m.RH._06/30
HD	2020_Kincaid	216	Avg1.5m.RH._07/02
HD	2020_Kincaid	216	Avg1.5m.RH._07/03
HD	2020_Kincaid	216	Avg1.5m.RH._07/04
HD	2020_Kincaid	216	Avg1.5m.RH._07/05
HD	2020_Kincaid	216	Avg1.5m.RH._07/06
HD	2020_Kincaid	216	Avg1.5m.RH._07/07
HD	2020_Kincaid	216	Avg1.5m.RH._07/08
HD	2020_Kincaid	216	Min.F.1_10/29
HD	2020_Kincaid	216	Min.F.1_11/03
HD	2020_Kincaid	216	Min.F.1_06/23
HD	2020_Kincaid	216	Min.F.1_06/24
HD	2020_Kincaid	216	Min.F.1_06/25
HD	2020_Kincaid	216	Min.F.1_06/26
HD	2020_Kincaid	216	Min.F.1_06/27
HD	2020_Kincaid	216	Avg.F.1_10/28
HD	2020_Kincaid	216	Avg.F.1_10/29
HD	2020_Kincaid	216	Avg.F.1_06/21
HD	2020_Kincaid	216	Avg.F.1_06/22
HD	2020_Kincaid	216	Avg.F.1_06/23
HD	2020_Kincaid	216	Avg.F.1_06/24
HD	2020_Kincaid	216	Avg.F.1_06/25
HD	2020_Kincaid	216	Avg.F.1_06/26
HD	2020_Kincaid	216	Avg.F.1_06/27
HD	2020_Kincaid	216	TotPrecin_11/04
HD	2020_Kincaid	216	TotPrecin_01/01
HD	2020_Kincaid	216	TotPrecin_04/19
HD	2020_Kincaid	216	TotPrecin_06/05
HD	2020_Kincaid	216	TotalSolarRadMJ.m._10/26
HD	2020_Kincaid	216	TotalSolarRadMJ.m._11/08

HD	2020_Kincaid	216	TotalSolarRadMJ.m._12/11
HD	2020_Kincaid	216	TotalSolarRadMJ.m._01/20
HD	2020_Kincaid	216	TotalSolarRadMJ.m._01/31
HD	2020_Kincaid	216	TotalSolarRadMJ.m._02/09
HD	2020_Kincaid	216	TotalSolarRadMJ.m._02/27
HD	2020_Kincaid	216	TotalSolarRadMJ.m._03/05
HD	2020_Kincaid	216	TotalSolarRadMJ.m._04/11
HD	2020_Kincaid	216	TotalSolarRadMJ.m._04/24
HD	2020_Kincaid	216	EToin_11/14
HD	2020_Kincaid	216	EToin_03/02
HD	2020_Kincaid	216	EToin_03/03
HD	2020_Kincaid	216	EToin_03/04
HD	2020_Kincaid	216	EToin_04/12
HD	2020_Kincaid	216	EToin_04/13
HD	2020_Kincaid	216	EToin_04/14
HD	2020_Kincaid	216	EToin_04/15
HD	2020_Kincaid	216	EToin_04/16
HD	2020_Kincaid	216	EToin_04/17
HD	2020_Kincaid	216	EToin_04/18
HD	2020_Kincaid	216	EToin_04/20
HD	2020_Kincaid	216	EToin_04/28
HD	2020_Kincaid	216	EToin_04/29
HD	2020_Kincaid	216	EToin_05/15
HD	2020_Kincaid	216	EToin_05/16
HD	2020_Kincaid	216	EToin_05/21
HD	2020_Kincaid	216	EToin_05/26
HD	2020_Kincaid	216	EToin_05/27
HD	2020_Kincaid	216	EToin_06/20
HD	2020_Kincaid	216	EToin_06/22
HD	2020_Kincaid	216	EToin_06/23
HD	2020_Kincaid	216	EToin_06/24
HD	2020_Kincaid	216	EToin_06/25
HD	2020_Kincaid	216	EToin_06/26
HD	2020_Kincaid	216	EToin_06/29
HD	2020_Kincaid	216	EToin_06/30
HD	2020_Kincaid	216	EToin_07/02
HD	2020_Kincaid	216	EToin_07/03
HD	2020_Kincaid	216	EToin_07/04
HD	2020_Kincaid	216	EToin_07/05
HD	2020_Kincaid	216	EToin_07/06
HD	2020_Kincaid	216	EToin_07/07
HD	2020_Kincaid	216	EToin_07/08
HD	2020_Kincaid	216	ETrin_11/14
HD	2020_Kincaid	216	ETrin_03/02

HD	2020_Kincaid	216	ETrin_03/03
HD	2020_Kincaid	216	ETrin_03/04
HD	2020_Kincaid	216	ETrin_04/12
HD	2020_Kincaid	216	ETrin_04/14
HD	2020_Kincaid	216	ETrin_04/15
HD	2020_Kincaid	216	ETrin_04/16
HD	2020_Kincaid	216	ETrin_04/18
HD	2020_Kincaid	216	ETrin_04/20
HD	2020_Kincaid	216	ETrin_04/24
HD	2020_Kincaid	216	ETrin_04/29
HD	2020_Kincaid	216	ETrin_05/15
HD	2020_Kincaid	216	ETrin_05/16
HD	2020_Kincaid	216	ETrin_05/21
HD	2020_Kincaid	216	ETrin_05/26
HD	2020_Kincaid	216	ETrin_05/27
HD	2020_Kincaid	216	ETrin_06/08
HD	2020_Kincaid	216	ETrin_06/20
HD	2020_Kincaid	216	ETrin_06/21
HD	2020_Kincaid	216	ETrin_06/22
HD	2020_Kincaid	216	ETrin_06/23
HD	2020_Kincaid	216	ETrin_06/24
HD	2020_Kincaid	216	ETrin_06/25
HD	2020_Kincaid	216	ETrin_06/26
HD	2020_Kincaid	216	ETrin_06/29
HD	2020_Kincaid	216	ETrin_06/30
HD	2020_Kincaid	216	ETrin_07/01
HD	2020_Kincaid	216	ETrin_07/02
HD	2020_Kincaid	216	ETrin_07/03
HD	2020_Kincaid	216	ETrin_07/04
HD	2020_Kincaid	216	ETrin_07/05
HD	2020_Kincaid	216	ETrin_07/06
HD	2020_Kincaid	216	ETrin_07/07
HD	2020_Kincaid	216	ETrin_07/08
HD	2020_Ritzville	140	Min.F_10/24
HD	2020_Ritzville	140	Min.F_11/27
HD	2020_Ritzville	140	Min.F_12/02
HD	2020_Ritzville	140	Min.F_12/23
HD	2020_Ritzville	140	Min.F_01/19
HD	2020_Ritzville	140	Min.F_03/23
HD	2020_Ritzville	140	Min.F_06/22
HD	2020_Ritzville	140	Min.F_06/23
HD	2020_Ritzville	140	Min.F_06/26
HD	2020_Ritzville	140	Min.F_06/29
HD	2020_Ritzville	140	Min.F_06/30

HD	2020_Ritzville	140	Min.F_07/01
HD	2020_Ritzville	140	Avg.F_04/15
HD	2020_Ritzville	140	Avg.F_05/16
HD	2020_Ritzville	140	Avg.F_05/17
HD	2020_Ritzville	140	Avg.F_06/19
HD	2020_Ritzville	140	Avg.F_06/22
HD	2020_Ritzville	140	Avg.F_06/23
HD	2020_Ritzville	140	Avg.F_06/25
HD	2020_Ritzville	140	Avg.F_06/26
HD	2020_Ritzville	140	Avg.F_06/29
HD	2020_Ritzville	140	Avg.F_06/30
HD	2020_Ritzville	140	Avg.F_07/03
HD	2020_Ritzville	140	Avg.F_07/04
HD	2020_Ritzville	140	Avg.F_07/05
HD	2020_Ritzville	140	Avg.F_07/06
HD	2020_Ritzville	140	Max.F_04/06
HD	2020_Ritzville	140	Max.F_04/15
HD	2020_Ritzville	140	Max.F_04/16
HD	2020_Ritzville	140	Max.F_04/30
HD	2020_Ritzville	140	Max.F_05/16
HD	2020_Ritzville	140	Max.F_06/03
HD	2020_Ritzville	140	Max.F_06/19
HD	2020_Ritzville	140	Max.F_06/22
HD	2020_Ritzville	140	Max.F_06/24
HD	2020_Ritzville	140	Max.F_06/25
HD	2020_Ritzville	140	Max.F_06/29
HD	2020_Ritzville	140	Max.F_07/03
HD	2020_Ritzville	140	Max.F_07/04
HD	2020_Ritzville	140	Max.F_07/06
HD	2020_Ritzville	140	Max.F_07/07
HD	2020_Ritzville	140	Avg1.5m.DP.F_10/24
HD	2020_Ritzville	140	Avg1.5m.DP.F_01/04
HD	2020_Ritzville	140	Avg1.5m.DP.F_01/12
HD	2020_Ritzville	140	Avg1.5m.DP.F_05/27
HD	2020_Ritzville	140	Avg1.5m.DP.F_06/06
HD	2020_Ritzville	140	Avg1.5m.DP.F_06/17
HD	2020_Ritzville	140	Avg1.5m.RH._11/26
HD	2020_Ritzville	140	Avg1.5m.RH._02/27
HD	2020_Ritzville	140	Avg1.5m.RH._04/10
HD	2020_Ritzville	140	Avg1.5m.RH._04/18
HD	2020_Ritzville	140	Avg1.5m.RH._04/19
HD	2020_Ritzville	140	Avg1.5m.RH._04/21
HD	2020_Ritzville	140	Avg1.5m.RH._05/15
HD	2020_Ritzville	140	Avg1.5m.RH._05/16

HD	2020_Ritzville	140	Avg1.5m.RH._05/21
HD	2020_Ritzville	140	Avg1.5m.RH._05/27
HD	2020_Ritzville	140	Avg1.5m.RH._06/02
HD	2020_Ritzville	140	Avg1.5m.RH._06/06
HD	2020_Ritzville	140	Avg1.5m.RH._06/08
HD	2020_Ritzville	140	Avg1.5m.RH._06/21
HD	2020_Ritzville	140	Avg1.5m.RH._06/22
HD	2020_Ritzville	140	Avg1.5m.RH._06/25
HD	2020_Ritzville	140	Avg1.5m.RH._06/30
HD	2020_Ritzville	140	Avg1.5m.RH._07/02
HD	2020_Ritzville	140	Avg1.5m.RH._07/03
HD	2020_Ritzville	140	Avg1.5m.RH._07/04
HD	2020_Ritzville	140	Avg1.5m.RH._07/05
HD	2020_Ritzville	140	Avg1.5m.RH._07/06
HD	2020_Ritzville	140	Avg1.5m.RH._07/07
HD	2020_Ritzville	140	Avg1.5m.RH._07/08
HD	2020_Ritzville	140	Min.F.1_06/22
HD	2020_Ritzville	140	Min.F.1_06/23
HD	2020_Ritzville	140	Min.F.1_06/24
HD	2020_Ritzville	140	Min.F.1_06/25
HD	2020_Ritzville	140	Min.F.1_06/26
HD	2020_Ritzville	140	Min.F.1_06/27
HD	2020_Ritzville	140	Min.F.1_06/29
HD	2020_Ritzville	140	Min.F.1_06/30
HD	2020_Ritzville	140	Min.F.1_07/04
HD	2020_Ritzville	140	Min.F.1_07/05
HD	2020_Ritzville	140	Avg.F.1_06/21
HD	2020_Ritzville	140	Avg.F.1_06/22
HD	2020_Ritzville	140	Avg.F.1_06/23
HD	2020_Ritzville	140	Avg.F.1_06/24
HD	2020_Ritzville	140	Avg.F.1_06/25
HD	2020_Ritzville	140	Avg.F.1_06/26
HD	2020_Ritzville	140	Avg.F.1_06/27
HD	2020_Ritzville	140	Avg.F.1_06/28
HD	2020_Ritzville	140	Avg.F.1_06/29
HD	2020_Ritzville	140	Avg.F.1_06/30
HD	2020_Ritzville	140	Avg.F.1_07/05
HD	2020_Ritzville	140	TotPrecin_01/01
HD	2020_Ritzville	140	TotPrecin_06/05
HD	2020_Ritzville	140	TotalSolarRadMJ.m._11/08
HD	2020_Ritzville	140	EToin_04/12
HD	2020_Ritzville	140	EToin_04/14
HD	2020_Ritzville	140	EToin_04/15
HD	2020_Ritzville	140	EToin_04/16

HD	2020_Ritzville	140	EToin_04/18
HD	2020_Ritzville	140	EToin_05/15
HD	2020_Ritzville	140	EToin_05/16
HD	2020_Ritzville	140	EToin_06/20
HD	2020_Ritzville	140	EToin_06/22
HD	2020_Ritzville	140	EToin_06/23
HD	2020_Ritzville	140	EToin_06/24
HD	2020_Ritzville	140	EToin_06/25
HD	2020_Ritzville	140	EToin_06/29
HD	2020_Ritzville	140	EToin_06/30
HD	2020_Ritzville	140	EToin_07/02
HD	2020_Ritzville	140	EToin_07/03
HD	2020_Ritzville	140	EToin_07/04
HD	2020_Ritzville	140	EToin_07/05
HD	2020_Ritzville	140	EToin_07/06
HD	2020_Ritzville	140	EToin_07/07
HD	2020_Ritzville	140	EToin_07/08
HD	2020_Ritzville	140	ETrin_04/14
HD	2020_Ritzville	140	ETrin_04/15
HD	2020_Ritzville	140	ETrin_04/16
HD	2020_Ritzville	140	ETrin_04/18
HD	2020_Ritzville	140	ETrin_05/15
HD	2020_Ritzville	140	ETrin_05/16
HD	2020_Ritzville	140	ETrin_05/26
HD	2020_Ritzville	140	ETrin_05/27
HD	2020_Ritzville	140	ETrin_06/21
HD	2020_Ritzville	140	ETrin_06/22
HD	2020_Ritzville	140	ETrin_06/23
HD	2020_Ritzville	140	ETrin_06/24
HD	2020_Ritzville	140	ETrin_06/25
HD	2020_Ritzville	140	ETrin_06/26
HD	2020_Ritzville	140	ETrin_06/29
HD	2020_Ritzville	140	ETrin_06/30
HD	2020_Ritzville	140	ETrin_07/01
HD	2020_Ritzville	140	ETrin_07/02
HD	2020_Ritzville	140	ETrin_07/03
HD	2020_Ritzville	140	ETrin_07/04
HD	2020_Ritzville	140	ETrin_07/05
HD	2020_Ritzville	140	ETrin_07/06
HD	2020_Ritzville	140	ETrin_07/07
HD	2020_Ritzville	140	ETrin_07/08
HD	2020_Walla_Walla	136	Min.F_11/27
HD	2020_Walla_Walla	136	Min.F_12/02
HD	2020_Walla_Walla	136	Min.F_12/23

HD	2020_Walla_Walla	136	Min.F_01/19
HD	2020_Walla_Walla	136	Min.F_03/23
HD	2020_Walla_Walla	136	Min.F_06/06
HD	2020_Walla_Walla	136	Min.F_06/23
HD	2020_Walla_Walla	136	Min.F_06/26
HD	2020_Walla_Walla	136	Avg.F_04/15
HD	2020_Walla_Walla	136	Avg.F_05/16
HD	2020_Walla_Walla	136	Avg.F_05/17
HD	2020_Walla_Walla	136	Avg.F_06/19
HD	2020_Walla_Walla	136	Avg.F_06/22
HD	2020_Walla_Walla	136	Avg.F_06/23
HD	2020_Walla_Walla	136	Avg.F_06/25
HD	2020_Walla_Walla	136	Avg.F_06/26
HD	2020_Walla_Walla	136	Avg.F_06/29
HD	2020_Walla_Walla	136	Avg.F_06/30
HD	2020_Walla_Walla	136	Avg.F_07/03
HD	2020_Walla_Walla	136	Avg.F_07/04
HD	2020_Walla_Walla	136	Avg.F_07/06
HD	2020_Walla_Walla	136	Max.F_04/06
HD	2020_Walla_Walla	136	Max.F_04/15
HD	2020_Walla_Walla	136	Max.F_04/16
HD	2020_Walla_Walla	136	Max.F_04/30
HD	2020_Walla_Walla	136	Max.F_05/16
HD	2020_Walla_Walla	136	Max.F_06/03
HD	2020_Walla_Walla	136	Max.F_06/19
HD	2020_Walla_Walla	136	Max.F_06/22
HD	2020_Walla_Walla	136	Max.F_06/24
HD	2020_Walla_Walla	136	Max.F_06/25
HD	2020_Walla_Walla	136	Max.F_06/29
HD	2020_Walla_Walla	136	Max.F_07/03
HD	2020_Walla_Walla	136	Max.F_07/06
HD	2020_Walla_Walla	136	Max.F_07/07
HD	2020_Walla_Walla	136	Avg1.5m.DP.F_10/24
HD	2020_Walla_Walla	136	Avg1.5m.DP.F_01/12
HD	2020_Walla_Walla	136	Avg1.5m.DP.F_02/12
HD	2020_Walla_Walla	136	Avg1.5m.DP.F_02/14
HD	2020_Walla_Walla	136	Avg1.5m.DP.F_03/29
HD	2020_Walla_Walla	136	Avg1.5m.DP.F_05/07
HD	2020_Walla_Walla	136	Avg1.5m.DP.F_05/26
HD	2020_Walla_Walla	136	Avg1.5m.DP.F_05/27
HD	2020_Walla_Walla	136	Avg1.5m.DP.F_06/06
HD	2020_Walla_Walla	136	Avg1.5m.DP.F_06/17
HD	2020_Walla_Walla	136	Avg1.5m.RH._11/26
HD	2020_Walla_Walla	136	Avg1.5m.RH._02/27

HD	2020_Walla_Walla	136	Avg1.5m.RH._04/10
HD	2020_Walla_Walla	136	Avg1.5m.RH._04/15
HD	2020_Walla_Walla	136	Avg1.5m.RH._04/19
HD	2020_Walla_Walla	136	Avg1.5m.RH._04/21
HD	2020_Walla_Walla	136	Avg1.5m.RH._05/15
HD	2020_Walla_Walla	136	Avg1.5m.RH._05/16
HD	2020_Walla_Walla	136	Avg1.5m.RH._05/21
HD	2020_Walla_Walla	136	Avg1.5m.RH._05/27
HD	2020_Walla_Walla	136	Avg1.5m.RH._05/28
HD	2020_Walla_Walla	136	Avg1.5m.RH._05/29
HD	2020_Walla_Walla	136	Avg1.5m.RH._06/02
HD	2020_Walla_Walla	136	Avg1.5m.RH._06/06
HD	2020_Walla_Walla	136	Avg1.5m.RH._06/08
HD	2020_Walla_Walla	136	Avg1.5m.RH._06/21
HD	2020_Walla_Walla	136	Avg1.5m.RH._06/22
HD	2020_Walla_Walla	136	Avg1.5m.RH._06/25
HD	2020_Walla_Walla	136	Avg1.5m.RH._07/02
HD	2020_Walla_Walla	136	Avg1.5m.RH._07/03
HD	2020_Walla_Walla	136	Avg1.5m.RH._07/04
HD	2020_Walla_Walla	136	Avg1.5m.RH._07/05
HD	2020_Walla_Walla	136	Avg1.5m.RH._07/06
HD	2020_Walla_Walla	136	Avg1.5m.RH._07/07
HD	2020_Walla_Walla	136	Avg1.5m.RH._07/08
HD	2020_Walla_Walla	136	Min.F.1_06/23
HD	2020_Walla_Walla	136	Min.F.1_06/24
HD	2020_Walla_Walla	136	Min.F.1_06/26
HD	2020_Walla_Walla	136	Min.F.1_06/27
HD	2020_Walla_Walla	136	Avg.F.1_06/23
HD	2020_Walla_Walla	136	Avg.F.1_06/24
HD	2020_Walla_Walla	136	Avg.F.1_06/25
HD	2020_Walla_Walla	136	Avg.F.1_06/26
HD	2020_Walla_Walla	136	Avg.F.1_06/27
HD	2020_Walla_Walla	136	TotPrecin_01/01
HD	2020_Walla_Walla	136	TotPrecin_06/05
HD	2020_Walla_Walla	136	TotalSolarRadMJ.m._11/08
HD	2020_Walla_Walla	136	TotalSolarRadMJ.m._11/16
HD	2020_Walla_Walla	136	TotalSolarRadMJ.m._03/05
HD	2020_Walla_Walla	136	EToin_10/30
HD	2020_Walla_Walla	136	EToin_12/18
HD	2020_Walla_Walla	136	EToin_04/12
HD	2020_Walla_Walla	136	EToin_04/14
HD	2020_Walla_Walla	136	EToin_04/15
HD	2020_Walla_Walla	136	EToin_04/16
HD	2020_Walla_Walla	136	EToin_04/18

HD	2020_Walla_Walla	136	EToin_05/15
HD	2020_Walla_Walla	136	EToin_05/16
HD	2020_Walla_Walla	136	EToin_06/20
HD	2020_Walla_Walla	136	EToin_06/22
HD	2020_Walla_Walla	136	EToin_06/23
HD	2020_Walla_Walla	136	EToin_06/24
HD	2020_Walla_Walla	136	EToin_06/25
HD	2020_Walla_Walla	136	EToin_06/26
HD	2020_Walla_Walla	136	EToin_06/29
HD	2020_Walla_Walla	136	EToin_06/30
HD	2020_Walla_Walla	136	EToin_07/02
HD	2020_Walla_Walla	136	EToin_07/03
HD	2020_Walla_Walla	136	EToin_07/04
HD	2020_Walla_Walla	136	EToin_07/05
HD	2020_Walla_Walla	136	EToin_07/06
HD	2020_Walla_Walla	136	EToin_07/07
HD	2020_Walla_Walla	136	EToin_07/08
HD	2020_Walla_Walla	136	ETrin_10/30
HD	2020_Walla_Walla	136	ETrin_12/18
HD	2020_Walla_Walla	136	ETrin_01/13
HD	2020_Walla_Walla	136	ETrin_04/14
HD	2020_Walla_Walla	136	ETrin_04/15
HD	2020_Walla_Walla	136	ETrin_04/16
HD	2020_Walla_Walla	136	ETrin_04/18
HD	2020_Walla_Walla	136	ETrin_05/15
HD	2020_Walla_Walla	136	ETrin_05/16
HD	2020_Walla_Walla	136	ETrin_05/26
HD	2020_Walla_Walla	136	ETrin_05/27
HD	2020_Walla_Walla	136	ETrin_06/20
HD	2020_Walla_Walla	136	ETrin_06/21
HD	2020_Walla_Walla	136	ETrin_06/22
HD	2020_Walla_Walla	136	ETrin_06/23
HD	2020_Walla_Walla	136	ETrin_06/24
HD	2020_Walla_Walla	136	ETrin_06/25
HD	2020_Walla_Walla	136	ETrin_06/26
HD	2020_Walla_Walla	136	ETrin_06/29
HD	2020_Walla_Walla	136	ETrin_06/30
HD	2020_Walla_Walla	136	ETrin_07/01
HD	2020_Walla_Walla	136	ETrin_07/02
HD	2020_Walla_Walla	136	ETrin_07/03
HD	2020_Walla_Walla	136	ETrin_07/04
HD	2020_Walla_Walla	136	ETrin_07/05
HD	2020_Walla_Walla	136	ETrin_07/06
HD	2020_Walla_Walla	136	ETrin_07/07

HD	2020_Walla_Walla	136	ETrin_07/08
HD	2021_Davenport	36	Min.F_06/23
HD	2021_Davenport	36	Avg.F_05/16
HD	2021_Davenport	36	Avg.F_06/19
HD	2021_Davenport	36	Avg.F_06/23
HD	2021_Davenport	36	Avg.F_07/04
HD	2021_Davenport	36	Max.F_04/06
HD	2021_Davenport	36	Max.F_04/30
HD	2021_Davenport	36	Max.F_05/16
HD	2021_Davenport	36	Max.F_06/25
HD	2021_Davenport	36	Max.F_07/03
HD	2021_Davenport	36	Avg1.5m.RH._05/27
HD	2021_Davenport	36	Avg1.5m.RH._06/06
HD	2021_Davenport	36	Avg1.5m.RH._06/08
HD	2021_Davenport	36	Avg1.5m.RH._06/22
HD	2021_Davenport	36	Avg1.5m.RH._07/03
HD	2021_Davenport	36	Avg1.5m.RH._07/04
HD	2021_Davenport	36	Avg1.5m.RH._07/05
HD	2021_Davenport	36	Avg1.5m.RH._07/06
HD	2021_Davenport	36	Min.F.1_06/24
HD	2021_Davenport	36	Min.F.1_06/26
HD	2021_Davenport	36	Avg.F.1_06/24
HD	2021_Davenport	36	Avg.F.1_06/26
HD	2021_Davenport	36	EToin_06/23
HD	2021_Davenport	36	EToin_06/24
HD	2021_Davenport	36	EToin_06/30
HD	2021_Davenport	36	EToin_07/03
HD	2021_Davenport	36	EToin_07/05
HD	2021_Davenport	36	EToin_07/06
HD	2021_Davenport	36	EToin_07/07
HD	2021_Davenport	36	ETrin_06/24
HD	2021_Davenport	36	ETrin_06/26
HD	2021_Davenport	36	ETrin_06/30
HD	2021_Davenport	36	ETrin_07/03
HD	2021_Davenport	36	ETrin_07/04
HD	2021_Davenport	36	ETrin_07/05
HD	2021_Davenport	36	ETrin_07/07
HD	2021_Harrington	95	Min.F_11/27
HD	2021_Harrington	95	Min.F_12/02
HD	2021_Harrington	95	Min.F_01/19
HD	2021_Harrington	95	Min.F_03/23
HD	2021_Harrington	95	Min.F_06/23
HD	2021_Harrington	95	Min.F_06/26
HD	2021_Harrington	95	Avg.F_04/15

HD	2021_Harrington	95	Avg.F_05/16
HD	2021_Harrington	95	Avg.F_05/17
HD	2021_Harrington	95	Avg.F_06/19
HD	2021_Harrington	95	Avg.F_06/23
HD	2021_Harrington	95	Avg.F_06/25
HD	2021_Harrington	95	Avg.F_06/26
HD	2021_Harrington	95	Avg.F_06/29
HD	2021_Harrington	95	Avg.F_07/03
HD	2021_Harrington	95	Avg.F_07/04
HD	2021_Harrington	95	Avg.F_07/06
HD	2021_Harrington	95	Max.F_04/15
HD	2021_Harrington	95	Max.F_04/16
HD	2021_Harrington	95	Max.F_04/30
HD	2021_Harrington	95	Max.F_05/16
HD	2021_Harrington	95	Max.F_06/03
HD	2021_Harrington	95	Max.F_06/22
HD	2021_Harrington	95	Max.F_06/24
HD	2021_Harrington	95	Max.F_06/25
HD	2021_Harrington	95	Max.F_06/29
HD	2021_Harrington	95	Max.F_07/03
HD	2021_Harrington	95	Max.F_07/06
HD	2021_Harrington	95	Avg1.5m.DP.F_01/12
HD	2021_Harrington	95	Avg1.5m.DP.F_05/27
HD	2021_Harrington	95	Avg1.5m.DP.F_06/06
HD	2021_Harrington	95	Avg1.5m.RH._11/26
HD	2021_Harrington	95	Avg1.5m.RH._04/10
HD	2021_Harrington	95	Avg1.5m.RH._04/19
HD	2021_Harrington	95	Avg1.5m.RH._04/21
HD	2021_Harrington	95	Avg1.5m.RH._05/15
HD	2021_Harrington	95	Avg1.5m.RH._05/21
HD	2021_Harrington	95	Avg1.5m.RH._05/27
HD	2021_Harrington	95	Avg1.5m.RH._06/06
HD	2021_Harrington	95	Avg1.5m.RH._06/08
HD	2021_Harrington	95	Avg1.5m.RH._06/21
HD	2021_Harrington	95	Avg1.5m.RH._06/22
HD	2021_Harrington	95	Avg1.5m.RH._07/02
HD	2021_Harrington	95	Avg1.5m.RH._07/03
HD	2021_Harrington	95	Avg1.5m.RH._07/04
HD	2021_Harrington	95	Avg1.5m.RH._07/05
HD	2021_Harrington	95	Avg1.5m.RH._07/06
HD	2021_Harrington	95	Avg1.5m.RH._07/07
HD	2021_Harrington	95	Avg1.5m.RH._07/08
HD	2021_Harrington	95	Min.F.1_06/26
HD	2021_Harrington	95	Min.F.1_06/27

HD	2021_Harrington	95	Avg.F.1_06/23
HD	2021_Harrington	95	Avg.F.1_06/24
HD	2021_Harrington	95	Avg.F.1_06/25
HD	2021_Harrington	95	Avg.F.1_06/26
HD	2021_Harrington	95	Avg.F.1_06/27
HD	2021_Harrington	95	TotPrecin_06/05
HD	2021_Harrington	95	TotalSolarRadMJ.m._11/08
HD	2021_Harrington	95	ETоin_12/28
HD	2021_Harrington	95	EToin_04/14
HD	2021_Harrington	95	EToin_04/16
HD	2021_Harrington	95	EToin_05/16
HD	2021_Harrington	95	EToin_06/22
HD	2021_Harrington	95	EToin_06/23
HD	2021_Harrington	95	EToin_06/24
HD	2021_Harrington	95	EToin_06/25
HD	2021_Harrington	95	EToin_06/29
HD	2021_Harrington	95	EToin_06/30
HD	2021_Harrington	95	EToin_07/02
HD	2021_Harrington	95	EToin_07/03
HD	2021_Harrington	95	EToin_07/04
HD	2021_Harrington	95	EToin_07/05
HD	2021_Harrington	95	EToin_07/06
HD	2021_Harrington	95	EToin_07/07
HD	2021_Harrington	95	ETrin_04/14
HD	2021_Harrington	95	ETrin_04/16
HD	2021_Harrington	95	ETrin_04/18
HD	2021_Harrington	95	ETrin_05/16
HD	2021_Harrington	95	ETrin_05/26
HD	2021_Harrington	95	ETrin_05/27
HD	2021_Harrington	95	ETrin_06/21
HD	2021_Harrington	95	ETrin_06/22
HD	2021_Harrington	95	ETrin_06/23
HD	2021_Harrington	95	ETrin_06/24
HD	2021_Harrington	95	ETrin_06/25
HD	2021_Harrington	95	ETrin_06/26
HD	2021_Harrington	95	ETrin_06/29
HD	2021_Harrington	95	ETrin_06/30
HD	2021_Harrington	95	ETrin_07/02
HD	2021_Harrington	95	ETrin_07/03
HD	2021_Harrington	95	ETrin_07/04
HD	2021_Harrington	95	ETrin_07/05
HD	2021_Harrington	95	ETrin_07/06
HD	2021_Harrington	95	ETrin_07/07
HD	2021_Harrington	95	ETrin_07/08

HD	2021_Kahlotus	93	Min.F_11/27
HD	2021_Kahlotus	93	Min.F_12/02
HD	2021_Kahlotus	93	Min.F_12/23
HD	2021_Kahlotus	93	Min.F_01/19
HD	2021_Kahlotus	93	Min.F_03/23
HD	2021_Kahlotus	93	Min.F_06/23
HD	2021_Kahlotus	93	Min.F_06/26
HD	2021_Kahlotus	93	Avg.F_04/15
HD	2021_Kahlotus	93	Avg.F_05/16
HD	2021_Kahlotus	93	Avg.F_05/17
HD	2021_Kahlotus	93	Avg.F_06/19
HD	2021_Kahlotus	93	Avg.F_06/23
HD	2021_Kahlotus	93	Avg.F_06/25
HD	2021_Kahlotus	93	Avg.F_06/29
HD	2021_Kahlotus	93	Avg.F_07/03
HD	2021_Kahlotus	93	Avg.F_07/04
HD	2021_Kahlotus	93	Avg.F_07/06
HD	2021_Kahlotus	93	Max.F_04/15
HD	2021_Kahlotus	93	Max.F_04/16
HD	2021_Kahlotus	93	Max.F_04/30
HD	2021_Kahlotus	93	Max.F_05/16
HD	2021_Kahlotus	93	Max.F_06/03
HD	2021_Kahlotus	93	Max.F_06/22
HD	2021_Kahlotus	93	Max.F_06/24
HD	2021_Kahlotus	93	Max.F_06/25
HD	2021_Kahlotus	93	Max.F_06/29
HD	2021_Kahlotus	93	Max.F_07/03
HD	2021_Kahlotus	93	Max.F_07/06
HD	2021_Kahlotus	93	Avg1.5m.DP.F_10/24
HD	2021_Kahlotus	93	Avg1.5m.DP.F_01/12
HD	2021_Kahlotus	93	Avg1.5m.DP.F_05/27
HD	2021_Kahlotus	93	Avg1.5m.DP.F_06/06
HD	2021_Kahlotus	93	Avg1.5m.RH._11/16
HD	2021_Kahlotus	93	Avg1.5m.RH._11/26
HD	2021_Kahlotus	93	Avg1.5m.RH._04/10
HD	2021_Kahlotus	93	Avg1.5m.RH._04/21
HD	2021_Kahlotus	93	Avg1.5m.RH._05/15
HD	2021_Kahlotus	93	Avg1.5m.RH._05/21
HD	2021_Kahlotus	93	Avg1.5m.RH._05/27
HD	2021_Kahlotus	93	Avg1.5m.RH._06/06
HD	2021_Kahlotus	93	Avg1.5m.RH._06/08
HD	2021_Kahlotus	93	Avg1.5m.RH._06/21
HD	2021_Kahlotus	93	Avg1.5m.RH._06/22
HD	2021_Kahlotus	93	Avg1.5m.RH._07/02

HD	2021_Kahlotus	93	Avg1.5m.RH._07/03
HD	2021_Kahlotus	93	Avg1.5m.RH._07/04
HD	2021_Kahlotus	93	Avg1.5m.RH._07/05
HD	2021_Kahlotus	93	Avg1.5m.RH._07/06
HD	2021_Kahlotus	93	Avg1.5m.RH._07/07
HD	2021_Kahlotus	93	Avg1.5m.RH._07/08
HD	2021_Kahlotus	93	Min.F.1_06/26
HD	2021_Kahlotus	93	Avg.F.1_06/26
HD	2021_Kahlotus	93	TotPrecin_01/01
HD	2021_Kahlotus	93	TotPrecin_06/05
HD	2021_Kahlotus	93	TotalSolarRadMJ.m._11/08
HD	2021_Kahlotus	93	TotalSolarRadMJ.m._03/05
HD	2021_Kahlotus	93	ETоin_12/18
HD	2021_Kahlotus	93	EToin_04/14
HD	2021_Kahlotus	93	EToin_04/16
HD	2021_Kahlotus	93	EToin_05/16
HD	2021_Kahlotus	93	EToin_06/22
HD	2021_Kahlotus	93	EToin_06/23
HD	2021_Kahlotus	93	EToin_06/24
HD	2021_Kahlotus	93	EToin_06/25
HD	2021_Kahlotus	93	EToin_06/29
HD	2021_Kahlotus	93	EToin_06/30
HD	2021_Kahlotus	93	EToin_07/02
HD	2021_Kahlotus	93	EToin_07/03
HD	2021_Kahlotus	93	EToin_07/04
HD	2021_Kahlotus	93	EToin_07/05
HD	2021_Kahlotus	93	EToin_07/06
HD	2021_Kahlotus	93	EToin_07/07
HD	2021_Kahlotus	93	ETrin_12/18
HD	2021_Kahlotus	93	ETrin_04/14
HD	2021_Kahlotus	93	ETrin_04/16
HD	2021_Kahlotus	93	ETrin_04/18
HD	2021_Kahlotus	93	ETrin_05/16
HD	2021_Kahlotus	93	ETrin_05/27
HD	2021_Kahlotus	93	ETrin_06/21
HD	2021_Kahlotus	93	ETrin_06/22
HD	2021_Kahlotus	93	ETrin_06/23
HD	2021_Kahlotus	93	ETrin_06/24
HD	2021_Kahlotus	93	ETrin_06/25
HD	2021_Kahlotus	93	ETrin_06/26
HD	2021_Kahlotus	93	ETrin_06/29
HD	2021_Kahlotus	93	ETrin_06/30
HD	2021_Kahlotus	93	ETrin_07/02
HD	2021_Kahlotus	93	ETrin_07/03

HD	2021_Kahlotus	93	ETrin_07/04
HD	2021_Kahlotus	93	ETrin_07/05
HD	2021_Kahlotus	93	ETrin_07/06
HD	2021_Kahlotus	93	ETrin_07/07
HD	2021_Kahlotus	93	ETrin_07/08
HD	2021_Kincaid	91	Min.F_11/27
HD	2021_Kincaid	91	Min.F_12/02
HD	2021_Kincaid	91	Min.F_01/19
HD	2021_Kincaid	91	Min.F_06/22
HD	2021_Kincaid	91	Min.F_06/23
HD	2021_Kincaid	91	Min.F_06/26
HD	2021_Kincaid	91	Min.F_07/05
HD	2021_Kincaid	91	Avg.F_12/24
HD	2021_Kincaid	91	Avg.F_05/16
HD	2021_Kincaid	91	Avg.F_05/17
HD	2021_Kincaid	91	Avg.F_06/19
HD	2021_Kincaid	91	Avg.F_06/23
HD	2021_Kincaid	91	Avg.F_06/25
HD	2021_Kincaid	91	Avg.F_06/26
HD	2021_Kincaid	91	Avg.F_07/03
HD	2021_Kincaid	91	Avg.F_07/04
HD	2021_Kincaid	91	Avg.F_07/05
HD	2021_Kincaid	91	Avg.F_07/06
HD	2021_Kincaid	91	Max.F_04/16
HD	2021_Kincaid	91	Max.F_04/30
HD	2021_Kincaid	91	Max.F_05/16
HD	2021_Kincaid	91	Max.F_06/03
HD	2021_Kincaid	91	Max.F_06/19
HD	2021_Kincaid	91	Max.F_06/22
HD	2021_Kincaid	91	Max.F_06/24
HD	2021_Kincaid	91	Max.F_06/25
HD	2021_Kincaid	91	Max.F_07/03
HD	2021_Kincaid	91	Max.F_07/06
HD	2021_Kincaid	91	Avg1.5m.DP.F_01/06
HD	2021_Kincaid	91	Avg1.5m.DP.F_01/12
HD	2021_Kincaid	91	Avg1.5m.DP.F_05/27
HD	2021_Kincaid	91	Avg1.5m.DP.F_06/06
HD	2021_Kincaid	91	Avg1.5m.RH._11/16
HD	2021_Kincaid	91	Avg1.5m.RH._11/26
HD	2021_Kincaid	91	Avg1.5m.RH._12/05
HD	2021_Kincaid	91	Avg1.5m.RH._02/27
HD	2021_Kincaid	91	Avg1.5m.RH._04/10
HD	2021_Kincaid	91	Avg1.5m.RH._05/15
HD	2021_Kincaid	91	Avg1.5m.RH._05/21

HD	2021_Kincaid	91	Avg1.5m.RH._05/27
HD	2021_Kincaid	91	Avg1.5m.RH._06/02
HD	2021_Kincaid	91	Avg1.5m.RH._06/06
HD	2021_Kincaid	91	Avg1.5m.RH._06/08
HD	2021_Kincaid	91	Avg1.5m.RH._06/21
HD	2021_Kincaid	91	Avg1.5m.RH._06/22
HD	2021_Kincaid	91	Avg1.5m.RH._06/25
HD	2021_Kincaid	91	Avg1.5m.RH._07/02
HD	2021_Kincaid	91	Avg1.5m.RH._07/03
HD	2021_Kincaid	91	Avg1.5m.RH._07/04
HD	2021_Kincaid	91	Avg1.5m.RH._07/05
HD	2021_Kincaid	91	Avg1.5m.RH._07/06
HD	2021_Kincaid	91	Avg1.5m.RH._07/07
HD	2021_Kincaid	91	TotPrecin_01/01
HD	2021_Kincaid	91	TotalSolarRadMJ.m._11/08
HD	2021_Kincaid	91	TotalSolarRadMJ.m._01/18
HD	2021_Kincaid	91	ETоin_12/28
HD	2021_Kincaid	91	EToin_05/16
HD	2021_Kincaid	91	EToin_05/21
HD	2021_Kincaid	91	EToin_06/22
HD	2021_Kincaid	91	EToin_06/23
HD	2021_Kincaid	91	EToin_06/24
HD	2021_Kincaid	91	EToin_06/25
HD	2021_Kincaid	91	EToin_06/26
HD	2021_Kincaid	91	EToin_06/29
HD	2021_Kincaid	91	EToin_06/30
HD	2021_Kincaid	91	EToin_07/02
HD	2021_Kincaid	91	EToin_07/03
HD	2021_Kincaid	91	EToin_07/04
HD	2021_Kincaid	91	EToin_07/05
HD	2021_Kincaid	91	EToin_07/06
HD	2021_Kincaid	91	EToin_07/07
HD	2021_Kincaid	91	ETrin_12/18
HD	2021_Kincaid	91	ETrin_03/19
HD	2021_Kincaid	91	ETrin_04/16
HD	2021_Kincaid	91	ETrin_05/16
HD	2021_Kincaid	91	ETrin_05/21
HD	2021_Kincaid	91	ETrin_06/21
HD	2021_Kincaid	91	ETrin_06/22
HD	2021_Kincaid	91	ETrin_06/23
HD	2021_Kincaid	91	ETrin_06/24
HD	2021_Kincaid	91	ETrin_06/25
HD	2021_Kincaid	91	ETrin_06/26
HD	2021_Kincaid	91	ETrin_06/29

HD	2021_Kincaid	91	ETrin_06/30
HD	2021_Kincaid	91	ETrin_07/01
HD	2021_Kincaid	91	ETrin_07/02
HD	2021_Kincaid	91	ETrin_07/03
HD	2021_Kincaid	91	ETrin_07/04
HD	2021_Kincaid	91	ETrin_07/05
HD	2021_Kincaid	91	ETrin_07/06
HD	2021_Kincaid	91	ETrin_07/07
HD	2021_Pullman	339	Min.F_10/23
HD	2021_Pullman	339	Min.F_10/24
HD	2021_Pullman	339	Min.F_10/25
HD	2021_Pullman	339	Min.F_10/26
HD	2021_Pullman	339	Min.F_11/27
HD	2021_Pullman	339	Min.F_12/02
HD	2021_Pullman	339	Min.F_12/12
HD	2021_Pullman	339	Min.F_12/13
HD	2021_Pullman	339	Min.F_12/23
HD	2021_Pullman	339	Min.F_01/19
HD	2021_Pullman	339	Min.F_02/12
HD	2021_Pullman	339	Min.F_02/13
HD	2021_Pullman	339	Min.F_02/22
HD	2021_Pullman	339	Min.F_03/23
HD	2021_Pullman	339	Min.F_04/15
HD	2021_Pullman	339	Min.F_06/06
HD	2021_Pullman	339	Min.F_06/15
HD	2021_Pullman	339	Min.F_06/20
HD	2021_Pullman	339	Min.F_06/21
HD	2021_Pullman	339	Min.F_06/22
HD	2021_Pullman	339	Min.F_06/23
HD	2021_Pullman	339	Min.F_06/25
HD	2021_Pullman	339	Min.F_06/26
HD	2021_Pullman	339	Min.F_06/27
HD	2021_Pullman	339	Min.F_06/29
HD	2021_Pullman	339	Min.F_06/30
HD	2021_Pullman	339	Min.F_07/01
HD	2021_Pullman	339	Min.F_07/02
HD	2021_Pullman	339	Avg.F_10/23
HD	2021_Pullman	339	Avg.F_10/24
HD	2021_Pullman	339	Avg.F_10/25
HD	2021_Pullman	339	Avg.F_10/26
HD	2021_Pullman	339	Avg.F_11/27
HD	2021_Pullman	339	Avg.F_01/19
HD	2021_Pullman	339	Avg.F_02/12
HD	2021_Pullman	339	Avg.F_02/13

HD	2021_Pullman	339	Avg.F_02/14
HD	2021_Pullman	339	Avg.F_04/15
HD	2021_Pullman	339	Avg.F_04/30
HD	2021_Pullman	339	Avg.F_05/01
HD	2021_Pullman	339	Avg.F_05/06
HD	2021_Pullman	339	Avg.F_05/16
HD	2021_Pullman	339	Avg.F_05/17
HD	2021_Pullman	339	Avg.F_06/04
HD	2021_Pullman	339	Avg.F_06/19
HD	2021_Pullman	339	Avg.F_06/20
HD	2021_Pullman	339	Avg.F_06/21
HD	2021_Pullman	339	Avg.F_06/22
HD	2021_Pullman	339	Avg.F_06/23
HD	2021_Pullman	339	Avg.F_06/24
HD	2021_Pullman	339	Avg.F_06/25
HD	2021_Pullman	339	Avg.F_06/26
HD	2021_Pullman	339	Avg.F_06/28
HD	2021_Pullman	339	Avg.F_06/29
HD	2021_Pullman	339	Avg.F_06/30
HD	2021_Pullman	339	Avg.F_07/02
HD	2021_Pullman	339	Avg.F_07/03
HD	2021_Pullman	339	Avg.F_07/04
HD	2021_Pullman	339	Avg.F_07/05
HD	2021_Pullman	339	Avg.F_07/06
HD	2021_Pullman	339	Avg.F_07/07
HD	2021_Pullman	339	Avg.F_07/08
HD	2021_Pullman	339	Max.F_10/25
HD	2021_Pullman	339	Max.F_11/14
HD	2021_Pullman	339	Max.F_02/12
HD	2021_Pullman	339	Max.F_03/18
HD	2021_Pullman	339	Max.F_04/06
HD	2021_Pullman	339	Max.F_04/14
HD	2021_Pullman	339	Max.F_04/15
HD	2021_Pullman	339	Max.F_04/16
HD	2021_Pullman	339	Max.F_04/17
HD	2021_Pullman	339	Max.F_04/30
HD	2021_Pullman	339	Max.F_05/01
HD	2021_Pullman	339	Max.F_05/15
HD	2021_Pullman	339	Max.F_05/16
HD	2021_Pullman	339	Max.F_06/03
HD	2021_Pullman	339	Max.F_06/04
HD	2021_Pullman	339	Max.F_06/19
HD	2021_Pullman	339	Max.F_06/20
HD	2021_Pullman	339	Max.F_06/21

HD	2021_Pullman	339	Max.F_06/22
HD	2021_Pullman	339	Max.F_06/23
HD	2021_Pullman	339	Max.F_06/24
HD	2021_Pullman	339	Max.F_06/25
HD	2021_Pullman	339	Max.F_06/26
HD	2021_Pullman	339	Max.F_06/28
HD	2021_Pullman	339	Max.F_06/29
HD	2021_Pullman	339	Max.F_06/30
HD	2021_Pullman	339	Max.F_07/03
HD	2021_Pullman	339	Max.F_07/04
HD	2021_Pullman	339	Max.F_07/05
HD	2021_Pullman	339	Max.F_07/06
HD	2021_Pullman	339	Max.F_07/07
HD	2021_Pullman	339	Max.F_07/08
HD	2021_Pullman	339	Avg1.5m.DP.F_10/24
HD	2021_Pullman	339	Avg1.5m.DP.F_10/25
HD	2021_Pullman	339	Avg1.5m.DP.F_10/26
HD	2021_Pullman	339	Avg1.5m.DP.F_12/02
HD	2021_Pullman	339	Avg1.5m.DP.F_12/03
HD	2021_Pullman	339	Avg1.5m.DP.F_12/21
HD	2021_Pullman	339	Avg1.5m.DP.F_01/04
HD	2021_Pullman	339	Avg1.5m.DP.F_01/05
HD	2021_Pullman	339	Avg1.5m.DP.F_01/12
HD	2021_Pullman	339	Avg1.5m.DP.F_02/12
HD	2021_Pullman	339	Avg1.5m.DP.F_02/13
HD	2021_Pullman	339	Avg1.5m.DP.F_02/14
HD	2021_Pullman	339	Avg1.5m.DP.F_02/21
HD	2021_Pullman	339	Avg1.5m.DP.F_02/22
HD	2021_Pullman	339	Avg1.5m.DP.F_03/23
HD	2021_Pullman	339	Avg1.5m.DP.F_03/28
HD	2021_Pullman	339	Avg1.5m.DP.F_03/29
HD	2021_Pullman	339	Avg1.5m.DP.F_03/30
HD	2021_Pullman	339	Avg1.5m.DP.F_04/11
HD	2021_Pullman	339	Avg1.5m.DP.F_04/21
HD	2021_Pullman	339	Avg1.5m.DP.F_05/15
HD	2021_Pullman	339	Avg1.5m.DP.F_05/26
HD	2021_Pullman	339	Avg1.5m.DP.F_05/27
HD	2021_Pullman	339	Avg1.5m.DP.F_05/28
HD	2021_Pullman	339	Avg1.5m.DP.F_06/06
HD	2021_Pullman	339	Avg1.5m.DP.F_06/14
HD	2021_Pullman	339	Avg1.5m.DP.F_06/17
HD	2021_Pullman	339	Avg1.5m.DP.F_07/05
HD	2021_Pullman	339	Avg1.5m.RH._11/16
HD	2021_Pullman	339	Avg1.5m.RH._11/26

HD	2021_Pullman	339	Avg1.5m.RH._12/06
HD	2021_Pullman	339	Avg1.5m.RH._02/27
HD	2021_Pullman	339	Avg1.5m.RH._03/18
HD	2021_Pullman	339	Avg1.5m.RH._03/28
HD	2021_Pullman	339	Avg1.5m.RH._04/10
HD	2021_Pullman	339	Avg1.5m.RH._04/11
HD	2021_Pullman	339	Avg1.5m.RH._04/14
HD	2021_Pullman	339	Avg1.5m.RH._04/15
HD	2021_Pullman	339	Avg1.5m.RH._04/17
HD	2021_Pullman	339	Avg1.5m.RH._04/18
HD	2021_Pullman	339	Avg1.5m.RH._04/19
HD	2021_Pullman	339	Avg1.5m.RH._04/20
HD	2021_Pullman	339	Avg1.5m.RH._04/21
HD	2021_Pullman	339	Avg1.5m.RH._04/23
HD	2021_Pullman	339	Avg1.5m.RH._05/15
HD	2021_Pullman	339	Avg1.5m.RH._05/16
HD	2021_Pullman	339	Avg1.5m.RH._05/21
HD	2021_Pullman	339	Avg1.5m.RH._05/24
HD	2021_Pullman	339	Avg1.5m.RH._05/26
HD	2021_Pullman	339	Avg1.5m.RH._05/27
HD	2021_Pullman	339	Avg1.5m.RH._05/28
HD	2021_Pullman	339	Avg1.5m.RH._05/29
HD	2021_Pullman	339	Avg1.5m.RH._06/02
HD	2021_Pullman	339	Avg1.5m.RH._06/06
HD	2021_Pullman	339	Avg1.5m.RH._06/08
HD	2021_Pullman	339	Avg1.5m.RH._06/20
HD	2021_Pullman	339	Avg1.5m.RH._06/21
HD	2021_Pullman	339	Avg1.5m.RH._06/22
HD	2021_Pullman	339	Avg1.5m.RH._06/25
HD	2021_Pullman	339	Avg1.5m.RH._06/27
HD	2021_Pullman	339	Avg1.5m.RH._06/29
HD	2021_Pullman	339	Avg1.5m.RH._06/30
HD	2021_Pullman	339	Avg1.5m.RH._07/02
HD	2021_Pullman	339	Avg1.5m.RH._07/03
HD	2021_Pullman	339	Avg1.5m.RH._07/04
HD	2021_Pullman	339	Avg1.5m.RH._07/05
HD	2021_Pullman	339	Avg1.5m.RH._07/06
HD	2021_Pullman	339	Avg1.5m.RH._07/07
HD	2021_Pullman	339	Avg1.5m.RH._07/08
HD	2021_Pullman	339	Min.F.1_10/25
HD	2021_Pullman	339	Min.F.1_10/26
HD	2021_Pullman	339	Min.F.1_04/18
HD	2021_Pullman	339	Min.F.1_05/06
HD	2021_Pullman	339	Min.F.1_05/17

HD	2021_Pullman	339	Min.F.1_05/18
HD	2021_Pullman	339	Min.F.1_06/20
HD	2021_Pullman	339	Min.F.1_06/21
HD	2021_Pullman	339	Min.F.1_06/22
HD	2021_Pullman	339	Min.F.1_06/23
HD	2021_Pullman	339	Min.F.1_06/24
HD	2021_Pullman	339	Min.F.1_06/25
HD	2021_Pullman	339	Min.F.1_06/26
HD	2021_Pullman	339	Min.F.1_06/27
HD	2021_Pullman	339	Min.F.1_06/28
HD	2021_Pullman	339	Min.F.1_06/29
HD	2021_Pullman	339	Min.F.1_06/30
HD	2021_Pullman	339	Min.F.1_07/01
HD	2021_Pullman	339	Min.F.1_07/02
HD	2021_Pullman	339	Min.F.1_07/03
HD	2021_Pullman	339	Min.F.1_07/04
HD	2021_Pullman	339	Min.F.1_07/05
HD	2021_Pullman	339	Min.F.1_07/06
HD	2021_Pullman	339	Min.F.1_07/07
HD	2021_Pullman	339	Min.F.1_07/08
HD	2021_Pullman	339	Avg.F.1_10/25
HD	2021_Pullman	339	Avg.F.1_10/26
HD	2021_Pullman	339	Avg.F.1_04/17
HD	2021_Pullman	339	Avg.F.1_04/18
HD	2021_Pullman	339	Avg.F.1_05/01
HD	2021_Pullman	339	Avg.F.1_05/02
HD	2021_Pullman	339	Avg.F.1_05/03
HD	2021_Pullman	339	Avg.F.1_05/06
HD	2021_Pullman	339	Avg.F.1_05/17
HD	2021_Pullman	339	Avg.F.1_05/18
HD	2021_Pullman	339	Avg.F.1_06/20
HD	2021_Pullman	339	Avg.F.1_06/21
HD	2021_Pullman	339	Avg.F.1_06/22
HD	2021_Pullman	339	Avg.F.1_06/23
HD	2021_Pullman	339	Avg.F.1_06/24
HD	2021_Pullman	339	Avg.F.1_06/25
HD	2021_Pullman	339	Avg.F.1_06/26
HD	2021_Pullman	339	Avg.F.1_06/27
HD	2021_Pullman	339	Avg.F.1_06/28
HD	2021_Pullman	339	Avg.F.1_06/29
HD	2021_Pullman	339	Avg.F.1_06/30
HD	2021_Pullman	339	Avg.F.1_07/01
HD	2021_Pullman	339	Avg.F.1_07/02
HD	2021_Pullman	339	Avg.F.1_07/03

HD	2021_Pullman	339	Avg.F.1_07/04
HD	2021_Pullman	339	Avg.F.1_07/05
HD	2021_Pullman	339	Avg.F.1_07/06
HD	2021_Pullman	339	Avg.F.1_07/07
HD	2021_Pullman	339	Avg.F.1_07/08
HD	2021_Pullman	339	TotPrecin_12/09
HD	2021_Pullman	339	TotPrecin_12/15
HD	2021_Pullman	339	TotPrecin_12/21
HD	2021_Pullman	339	TotPrecin_01/01
HD	2021_Pullman	339	TotPrecin_01/04
HD	2021_Pullman	339	TotPrecin_01/12
HD	2021_Pullman	339	TotPrecin_06/05
HD	2021_Pullman	339	TotPrecin_06/15
HD	2021_Pullman	339	TotalSolarRadMJ.m._10/18
HD	2021_Pullman	339	TotalSolarRadMJ.m._10/26
HD	2021_Pullman	339	TotalSolarRadMJ.m._10/28
HD	2021_Pullman	339	TotalSolarRadMJ.m._11/08
HD	2021_Pullman	339	TotalSolarRadMJ.m._11/09
HD	2021_Pullman	339	TotalSolarRadMJ.m._11/16
HD	2021_Pullman	339	TotalSolarRadMJ.m._11/17
HD	2021_Pullman	339	TotalSolarRadMJ.m._11/27
HD	2021_Pullman	339	TotalSolarRadMJ.m._12/11
HD	2021_Pullman	339	TotalSolarRadMJ.m._12/23
HD	2021_Pullman	339	TotalSolarRadMJ.m._01/02
HD	2021_Pullman	339	TotalSolarRadMJ.m._01/03
HD	2021_Pullman	339	TotalSolarRadMJ.m._02/09
HD	2021_Pullman	339	TotalSolarRadMJ.m._02/18
HD	2021_Pullman	339	TotalSolarRadMJ.m._02/22
HD	2021_Pullman	339	TotalSolarRadMJ.m._03/05
HD	2021_Pullman	339	TotalSolarRadMJ.m._03/09
HD	2021_Pullman	339	TotalSolarRadMJ.m._04/14
HD	2021_Pullman	339	TotalSolarRadMJ.m._04/24
HD	2021_Pullman	339	TotalSolarRadMJ.m._04/27
HD	2021_Pullman	339	TotalSolarRadMJ.m._05/10
HD	2021_Pullman	339	TotalSolarRadMJ.m._05/15
HD	2021_Pullman	339	TotalSolarRadMJ.m._05/26
HD	2021_Pullman	339	EToin_10/23
HD	2021_Pullman	339	EToin_10/30
HD	2021_Pullman	339	EToin_11/09
HD	2021_Pullman	339	EToin_12/12
HD	2021_Pullman	339	EToin_01/13
HD	2021_Pullman	339	EToin_03/02
HD	2021_Pullman	339	EToin_03/18
HD	2021_Pullman	339	EToin_04/06

HD	2021_Pullman	339	EToin_04/12
HD	2021_Pullman	339	EToin_04/13
HD	2021_Pullman	339	EToin_04/14
HD	2021_Pullman	339	EToin_04/15
HD	2021_Pullman	339	EToin_04/16
HD	2021_Pullman	339	EToin_04/17
HD	2021_Pullman	339	EToin_04/18
HD	2021_Pullman	339	EToin_04/20
HD	2021_Pullman	339	EToin_04/24
HD	2021_Pullman	339	EToin_04/28
HD	2021_Pullman	339	EToin_04/29
HD	2021_Pullman	339	EToin_05/06
HD	2021_Pullman	339	EToin_05/15
HD	2021_Pullman	339	EToin_05/16
HD	2021_Pullman	339	EToin_05/26
HD	2021_Pullman	339	EToin_05/27
HD	2021_Pullman	339	EToin_06/08
HD	2021_Pullman	339	EToin_06/20
HD	2021_Pullman	339	EToin_06/21
HD	2021_Pullman	339	EToin_06/22
HD	2021_Pullman	339	EToin_06/23
HD	2021_Pullman	339	EToin_06/24
HD	2021_Pullman	339	EToin_06/25
HD	2021_Pullman	339	EToin_06/26
HD	2021_Pullman	339	EToin_06/27
HD	2021_Pullman	339	EToin_06/29
HD	2021_Pullman	339	EToin_06/30
HD	2021_Pullman	339	EToin_07/02
HD	2021_Pullman	339	EToin_07/03
HD	2021_Pullman	339	EToin_07/04
HD	2021_Pullman	339	EToin_07/05
HD	2021_Pullman	339	EToin_07/06
HD	2021_Pullman	339	EToin_07/07
HD	2021_Pullman	339	EToin_07/08
HD	2021_Pullman	339	ETrin_10/23
HD	2021_Pullman	339	ETrin_10/30
HD	2021_Pullman	339	ETrin_12/12
HD	2021_Pullman	339	ETrin_12/18
HD	2021_Pullman	339	ETrin_01/07
HD	2021_Pullman	339	ETrin_01/13
HD	2021_Pullman	339	ETrin_01/29
HD	2021_Pullman	339	ETrin_03/02
HD	2021_Pullman	339	ETrin_03/18
HD	2021_Pullman	339	ETrin_03/28

HD	2021_Pullman	339	ETrin_04/06
HD	2021_Pullman	339	ETrin_04/12
HD	2021_Pullman	339	ETrin_04/13
HD	2021_Pullman	339	ETrin_04/14
HD	2021_Pullman	339	ETrin_04/15
HD	2021_Pullman	339	ETrin_04/16
HD	2021_Pullman	339	ETrin_04/17
HD	2021_Pullman	339	ETrin_04/18
HD	2021_Pullman	339	ETrin_04/20
HD	2021_Pullman	339	ETrin_04/24
HD	2021_Pullman	339	ETrin_04/28
HD	2021_Pullman	339	ETrin_04/29
HD	2021_Pullman	339	ETrin_04/30
HD	2021_Pullman	339	ETrin_05/06
HD	2021_Pullman	339	ETrin_05/15
HD	2021_Pullman	339	ETrin_05/16
HD	2021_Pullman	339	ETrin_05/24
HD	2021_Pullman	339	ETrin_05/26
HD	2021_Pullman	339	ETrin_05/27
HD	2021_Pullman	339	ETrin_05/31
HD	2021_Pullman	339	ETrin_06/08
HD	2021_Pullman	339	ETrin_06/20
HD	2021_Pullman	339	ETrin_06/21
HD	2021_Pullman	339	ETrin_06/22
HD	2021_Pullman	339	ETrin_06/23
HD	2021_Pullman	339	ETrin_06/24
HD	2021_Pullman	339	ETrin_06/25
HD	2021_Pullman	339	ETrin_06/26
HD	2021_Pullman	339	ETrin_06/27
HD	2021_Pullman	339	ETrin_06/29
HD	2021_Pullman	339	ETrin_06/30
HD	2021_Pullman	339	ETrin_07/01
HD	2021_Pullman	339	ETrin_07/02
HD	2021_Pullman	339	ETrin_07/03
HD	2021_Pullman	339	ETrin_07/04
HD	2021_Pullman	339	ETrin_07/05
HD	2021_Pullman	339	ETrin_07/06
HD	2021_Pullman	339	ETrin_07/07
HD	2021_Pullman	339	ETrin_07/08
HD	2021_Ritzville	98	Min.F_11/27
HD	2021_Ritzville	98	Min.F_12/02
HD	2021_Ritzville	98	Min.F_01/19
HD	2021_Ritzville	98	Min.F_03/23
HD	2021_Ritzville	98	Min.F_06/23

HD	2021_Ritzville	98	Min.F_06/26
HD	2021_Ritzville	98	Avg.F_05/16
HD	2021_Ritzville	98	Avg.F_05/17
HD	2021_Ritzville	98	Avg.F_06/19
HD	2021_Ritzville	98	Avg.F_06/23
HD	2021_Ritzville	98	Avg.F_06/25
HD	2021_Ritzville	98	Avg.F_06/26
HD	2021_Ritzville	98	Avg.F_06/29
HD	2021_Ritzville	98	Avg.F_07/03
HD	2021_Ritzville	98	Avg.F_07/04
HD	2021_Ritzville	98	Avg.F_07/06
HD	2021_Ritzville	98	Max.F_04/15
HD	2021_Ritzville	98	Max.F_04/16
HD	2021_Ritzville	98	Max.F_04/30
HD	2021_Ritzville	98	Max.F_05/16
HD	2021_Ritzville	98	Max.F_06/03
HD	2021_Ritzville	98	Max.F_06/22
HD	2021_Ritzville	98	Max.F_06/24
HD	2021_Ritzville	98	Max.F_06/25
HD	2021_Ritzville	98	Max.F_06/29
HD	2021_Ritzville	98	Max.F_07/03
HD	2021_Ritzville	98	Max.F_07/06
HD	2021_Ritzville	98	Avg1.5m.DP.F_10/24
HD	2021_Ritzville	98	Avg1.5m.DP.F_01/12
HD	2021_Ritzville	98	Avg1.5m.DP.F_05/27
HD	2021_Ritzville	98	Avg1.5m.DP.F_06/06
HD	2021_Ritzville	98	Avg1.5m.RH._11/26
HD	2021_Ritzville	98	Avg1.5m.RH._04/10
HD	2021_Ritzville	98	Avg1.5m.RH._04/21
HD	2021_Ritzville	98	Avg1.5m.RH._05/15
HD	2021_Ritzville	98	Avg1.5m.RH._05/21
HD	2021_Ritzville	98	Avg1.5m.RH._05/27
HD	2021_Ritzville	98	Avg1.5m.RH._06/06
HD	2021_Ritzville	98	Avg1.5m.RH._06/08
HD	2021_Ritzville	98	Avg1.5m.RH._06/21
HD	2021_Ritzville	98	Avg1.5m.RH._06/22
HD	2021_Ritzville	98	Avg1.5m.RH._07/02
HD	2021_Ritzville	98	Avg1.5m.RH._07/03
HD	2021_Ritzville	98	Avg1.5m.RH._07/04
HD	2021_Ritzville	98	Avg1.5m.RH._07/05
HD	2021_Ritzville	98	Avg1.5m.RH._07/06
HD	2021_Ritzville	98	Avg1.5m.RH._07/07
HD	2021_Ritzville	98	Avg1.5m.RH._07/08
HD	2021_Ritzville	98	Min.F.1_06/23

HD	2021_Ritzville	98	Min.F.1_06/26
HD	2021_Ritzville	98	Min.F.1_06/27
HD	2021_Ritzville	98	Avg.F.1_06/23
HD	2021_Ritzville	98	Avg.F.1_06/24
HD	2021_Ritzville	98	Avg.F.1_06/25
HD	2021_Ritzville	98	Avg.F.1_06/26
HD	2021_Ritzville	98	Avg.F.1_06/27
HD	2021_Ritzville	98	TotPrecin_01/01
HD	2021_Ritzville	98	TotPrecin_06/05
HD	2021_Ritzville	98	TotalSolarRadMJ.m._11/08
HD	2021_Ritzville	98	TotalSolarRadMJ.m._03/05
HD	2021_Ritzville	98	ETpin_04/14
HD	2021_Ritzville	98	ETpin_04/16
HD	2021_Ritzville	98	ETpin_04/18
HD	2021_Ritzville	98	ETpin_05/16
HD	2021_Ritzville	98	ETpin_06/22
HD	2021_Ritzville	98	ETpin_06/23
HD	2021_Ritzville	98	ETpin_06/24
HD	2021_Ritzville	98	ETpin_06/25
HD	2021_Ritzville	98	ETpin_06/29
HD	2021_Ritzville	98	ETpin_06/30
HD	2021_Ritzville	98	ETpin_07/02
HD	2021_Ritzville	98	ETpin_07/03
HD	2021_Ritzville	98	ETpin_07/04
HD	2021_Ritzville	98	ETpin_07/05
HD	2021_Ritzville	98	ETpin_07/06
HD	2021_Ritzville	98	ETpin_07/07
HD	2021_Ritzville	98	ETrin_04/14
HD	2021_Ritzville	98	ETrin_04/16
HD	2021_Ritzville	98	ETrin_04/18
HD	2021_Ritzville	98	ETrin_05/15
HD	2021_Ritzville	98	ETrin_05/16
HD	2021_Ritzville	98	ETrin_05/26
HD	2021_Ritzville	98	ETrin_05/27
HD	2021_Ritzville	98	ETrin_06/21
HD	2021_Ritzville	98	ETrin_06/22
HD	2021_Ritzville	98	ETrin_06/23
HD	2021_Ritzville	98	ETrin_06/24
HD	2021_Ritzville	98	ETrin_06/25
HD	2021_Ritzville	98	ETrin_06/26
HD	2021_Ritzville	98	ETrin_06/29
HD	2021_Ritzville	98	ETrin_06/30
HD	2021_Ritzville	98	ETrin_07/02
HD	2021_Ritzville	98	ETrin_07/03

HD	2021_Ritzville	98	ETrin_07/04
HD	2021_Ritzville	98	ETrin_07/05
HD	2021_Ritzville	98	ETrin_07/06
HD	2021_Ritzville	98	ETrin_07/07
HD	2021_Ritzville	98	ETrin_07/08
HD	2021_Walla_Walla	96	Min.F_11/27
HD	2021_Walla_Walla	96	Min.F_01/19
HD	2021_Walla_Walla	96	Min.F_03/23
HD	2021_Walla_Walla	96	Min.F_06/23
HD	2021_Walla_Walla	96	Min.F_06/26
HD	2021_Walla_Walla	96	Avg.F_05/16
HD	2021_Walla_Walla	96	Avg.F_05/17
HD	2021_Walla_Walla	96	Avg.F_06/19
HD	2021_Walla_Walla	96	Avg.F_06/23
HD	2021_Walla_Walla	96	Avg.F_06/25
HD	2021_Walla_Walla	96	Avg.F_06/26
HD	2021_Walla_Walla	96	Avg.F_06/29
HD	2021_Walla_Walla	96	Avg.F_07/03
HD	2021_Walla_Walla	96	Avg.F_07/04
HD	2021_Walla_Walla	96	Avg.F_07/06
HD	2021_Walla_Walla	96	Max.F_04/06
HD	2021_Walla_Walla	96	Max.F_04/15
HD	2021_Walla_Walla	96	Max.F_04/16
HD	2021_Walla_Walla	96	Max.F_04/30
HD	2021_Walla_Walla	96	Max.F_05/16
HD	2021_Walla_Walla	96	Max.F_06/03
HD	2021_Walla_Walla	96	Max.F_06/22
HD	2021_Walla_Walla	96	Max.F_06/24
HD	2021_Walla_Walla	96	Max.F_06/25
HD	2021_Walla_Walla	96	Max.F_06/29
HD	2021_Walla_Walla	96	Max.F_07/03
HD	2021_Walla_Walla	96	Max.F_07/06
HD	2021_Walla_Walla	96	Avg1.5m.DP.F_10/24
HD	2021_Walla_Walla	96	Avg1.5m.DP.F_01/12
HD	2021_Walla_Walla	96	Avg1.5m.DP.F_05/27
HD	2021_Walla_Walla	96	Avg1.5m.DP.F_06/06
HD	2021_Walla_Walla	96	Avg1.5m.DP.F_06/17
HD	2021_Walla_Walla	96	Avg1.5m.RH._11/26
HD	2021_Walla_Walla	96	Avg1.5m.RH._04/10
HD	2021_Walla_Walla	96	Avg1.5m.RH._04/19
HD	2021_Walla_Walla	96	Avg1.5m.RH._04/21
HD	2021_Walla_Walla	96	Avg1.5m.RH._05/15
HD	2021_Walla_Walla	96	Avg1.5m.RH._05/21
HD	2021_Walla_Walla	96	Avg1.5m.RH._05/27

HD	2021_Walla_Walla	96	Avg1.5m.RH._06/02
HD	2021_Walla_Walla	96	Avg1.5m.RH._06/06
HD	2021_Walla_Walla	96	Avg1.5m.RH._06/08
HD	2021_Walla_Walla	96	Avg1.5m.RH._06/21
HD	2021_Walla_Walla	96	Avg1.5m.RH._06/22
HD	2021_Walla_Walla	96	Avg1.5m.RH._07/02
HD	2021_Walla_Walla	96	Avg1.5m.RH._07/03
HD	2021_Walla_Walla	96	Avg1.5m.RH._07/04
HD	2021_Walla_Walla	96	Avg1.5m.RH._07/05
HD	2021_Walla_Walla	96	Avg1.5m.RH._07/06
HD	2021_Walla_Walla	96	Avg1.5m.RH._07/07
HD	2021_Walla_Walla	96	Avg1.5m.RH._07/08
HD	2021_Walla_Walla	96	Min.F.1_06/26
HD	2021_Walla_Walla	96	Avg.F.1_06/26
HD	2021_Walla_Walla	96	TotPrecin_01/01
HD	2021_Walla_Walla	96	TotPrecin_01/25
HD	2021_Walla_Walla	96	TotPrecin_06/05
HD	2021_Walla_Walla	96	TotalSolarRadMJ.m._11/08
HD	2021_Walla_Walla	96	EToin_04/14
HD	2021_Walla_Walla	96	EToin_04/16
HD	2021_Walla_Walla	96	EToin_04/18
HD	2021_Walla_Walla	96	EToin_05/16
HD	2021_Walla_Walla	96	EToin_06/22
HD	2021_Walla_Walla	96	EToin_06/23
HD	2021_Walla_Walla	96	EToin_06/24
HD	2021_Walla_Walla	96	EToin_06/25
HD	2021_Walla_Walla	96	EToin_06/29
HD	2021_Walla_Walla	96	EToin_06/30
HD	2021_Walla_Walla	96	EToin_07/02
HD	2021_Walla_Walla	96	EToin_07/03
HD	2021_Walla_Walla	96	EToin_07/04
HD	2021_Walla_Walla	96	EToin_07/05
HD	2021_Walla_Walla	96	EToin_07/06
HD	2021_Walla_Walla	96	EToin_07/07
HD	2021_Walla_Walla	96	ETrin_04/14
HD	2021_Walla_Walla	96	ETrin_04/15
HD	2021_Walla_Walla	96	ETrin_04/16
HD	2021_Walla_Walla	96	ETrin_04/18
HD	2021_Walla_Walla	96	ETrin_05/16
HD	2021_Walla_Walla	96	ETrin_05/26
HD	2021_Walla_Walla	96	ETrin_05/27
HD	2021_Walla_Walla	96	ETrin_06/08
HD	2021_Walla_Walla	96	ETrin_06/21
HD	2021_Walla_Walla	96	ETrin_06/22

HD	2021_Walla_Walla	96	ETrin_06/23
HD	2021_Walla_Walla	96	ETrin_06/24
HD	2021_Walla_Walla	96	ETrin_06/25
HD	2021_Walla_Walla	96	ETrin_06/26
HD	2021_Walla_Walla	96	ETrin_06/29
HD	2021_Walla_Walla	96	ETrin_06/30
HD	2021_Walla_Walla	96	ETrin_07/02
HD	2021_Walla_Walla	96	ETrin_07/03
HD	2021_Walla_Walla	96	ETrin_07/04
HD	2021_Walla_Walla	96	ETrin_07/05
HD	2021_Walla_Walla	96	ETrin_07/06
HD	2021_Walla_Walla	96	ETrin_07/07
HD	2021_Walla_Walla	96	ETrin_07/08
HD	2022_Davenport	130	Min.F_11/27
HD	2022_Davenport	130	Min.F_12/02
HD	2022_Davenport	130	Min.F_12/23
HD	2022_Davenport	130	Min.F_01/19
HD	2022_Davenport	130	Min.F_03/23
HD	2022_Davenport	130	Min.F_06/22
HD	2022_Davenport	130	Min.F_06/23
HD	2022_Davenport	130	Min.F_06/26
HD	2022_Davenport	130	Min.F_06/30
HD	2022_Davenport	130	Min.F_07/01
HD	2022_Davenport	130	Avg.F_04/15
HD	2022_Davenport	130	Avg.F_05/16
HD	2022_Davenport	130	Avg.F_05/17
HD	2022_Davenport	130	Avg.F_06/19
HD	2022_Davenport	130	Avg.F_06/22
HD	2022_Davenport	130	Avg.F_06/23
HD	2022_Davenport	130	Avg.F_06/25
HD	2022_Davenport	130	Avg.F_06/26
HD	2022_Davenport	130	Avg.F_06/29
HD	2022_Davenport	130	Avg.F_07/03
HD	2022_Davenport	130	Avg.F_07/04
HD	2022_Davenport	130	Avg.F_07/06
HD	2022_Davenport	130	Max.F_04/06
HD	2022_Davenport	130	Max.F_04/15
HD	2022_Davenport	130	Max.F_04/16
HD	2022_Davenport	130	Max.F_04/30
HD	2022_Davenport	130	Max.F_05/16
HD	2022_Davenport	130	Max.F_06/03
HD	2022_Davenport	130	Max.F_06/19
HD	2022_Davenport	130	Max.F_06/20
HD	2022_Davenport	130	Max.F_06/22

HD	2022_Davenport	130	Max.F_06/24
HD	2022_Davenport	130	Max.F_06/25
HD	2022_Davenport	130	Max.F_06/29
HD	2022_Davenport	130	Max.F_07/03
HD	2022_Davenport	130	Max.F_07/06
HD	2022_Davenport	130	Max.F_07/07
HD	2022_Davenport	130	Avg1.5m.DP.F_10/24
HD	2022_Davenport	130	Avg1.5m.DP.F_01/12
HD	2022_Davenport	130	Avg1.5m.DP.F_05/27
HD	2022_Davenport	130	Avg1.5m.DP.F_06/06
HD	2022_Davenport	130	Avg1.5m.RH._11/16
HD	2022_Davenport	130	Avg1.5m.RH._11/26
HD	2022_Davenport	130	Avg1.5m.RH._02/27
HD	2022_Davenport	130	Avg1.5m.RH._04/10
HD	2022_Davenport	130	Avg1.5m.RH._04/19
HD	2022_Davenport	130	Avg1.5m.RH._04/21
HD	2022_Davenport	130	Avg1.5m.RH._05/15
HD	2022_Davenport	130	Avg1.5m.RH._05/16
HD	2022_Davenport	130	Avg1.5m.RH._05/21
HD	2022_Davenport	130	Avg1.5m.RH._05/27
HD	2022_Davenport	130	Avg1.5m.RH._06/02
HD	2022_Davenport	130	Avg1.5m.RH._06/06
HD	2022_Davenport	130	Avg1.5m.RH._06/08
HD	2022_Davenport	130	Avg1.5m.RH._06/21
HD	2022_Davenport	130	Avg1.5m.RH._06/22
HD	2022_Davenport	130	Avg1.5m.RH._06/25
HD	2022_Davenport	130	Avg1.5m.RH._06/26
HD	2022_Davenport	130	Avg1.5m.RH._07/02
HD	2022_Davenport	130	Avg1.5m.RH._07/03
HD	2022_Davenport	130	Avg1.5m.RH._07/04
HD	2022_Davenport	130	Avg1.5m.RH._07/05
HD	2022_Davenport	130	Avg1.5m.RH._07/06
HD	2022_Davenport	130	Avg1.5m.RH._07/07
HD	2022_Davenport	130	Avg1.5m.RH._07/08
HD	2022_Davenport	130	Min.F.1_06/23
HD	2022_Davenport	130	Min.F.1_06/24
HD	2022_Davenport	130	Min.F.1_06/26
HD	2022_Davenport	130	Min.F.1_06/27
HD	2022_Davenport	130	Min.F.1_06/30
HD	2022_Davenport	130	Avg.F.1_06/21
HD	2022_Davenport	130	Avg.F.1_06/22
HD	2022_Davenport	130	Avg.F.1_06/23
HD	2022_Davenport	130	Avg.F.1_06/24
HD	2022_Davenport	130	Avg.F.1_06/25

HD	2022_Davenport	130	Avg.F.1_06/26
HD	2022_Davenport	130	Avg.F.1_06/27
HD	2022_Davenport	130	TotPrecin_01/01
HD	2022_Davenport	130	TotPrecin_06/05
HD	2022_Davenport	130	TotalSolarRadMJ.m._11/08
HD	2022_Davenport	130	TotalSolarRadMJ.m._03/05
HD	2022_Davenport	130	TotalSolarRadMJ.m._05/26
HD	2022_Davenport	130	EToin_04/12
HD	2022_Davenport	130	EToin_04/14
HD	2022_Davenport	130	EToin_04/15
HD	2022_Davenport	130	EToin_04/16
HD	2022_Davenport	130	EToin_04/18
HD	2022_Davenport	130	EToin_05/15
HD	2022_Davenport	130	EToin_05/16
HD	2022_Davenport	130	EToin_05/26
HD	2022_Davenport	130	EToin_06/20
HD	2022_Davenport	130	EToin_06/22
HD	2022_Davenport	130	EToin_06/23
HD	2022_Davenport	130	EToin_06/24
HD	2022_Davenport	130	EToin_06/25
HD	2022_Davenport	130	EToin_06/26
HD	2022_Davenport	130	EToin_06/29
HD	2022_Davenport	130	EToin_06/30
HD	2022_Davenport	130	EToin_07/02
HD	2022_Davenport	130	EToin_07/03
HD	2022_Davenport	130	EToin_07/04
HD	2022_Davenport	130	EToin_07/05
HD	2022_Davenport	130	EToin_07/06
HD	2022_Davenport	130	EToin_07/07
HD	2022_Davenport	130	EToin_07/08
HD	2022_Davenport	130	ETrin_12/18
HD	2022_Davenport	130	ETrin_04/14
HD	2022_Davenport	130	ETrin_04/15
HD	2022_Davenport	130	ETrin_04/16
HD	2022_Davenport	130	ETrin_04/18
HD	2022_Davenport	130	ETrin_05/15
HD	2022_Davenport	130	ETrin_05/16
HD	2022_Davenport	130	ETrin_05/26
HD	2022_Davenport	130	ETrin_05/27
HD	2022_Davenport	130	ETrin_06/21
HD	2022_Davenport	130	ETrin_06/22
HD	2022_Davenport	130	ETrin_06/23
HD	2022_Davenport	130	ETrin_06/24
HD	2022_Davenport	130	ETrin_06/25

HD	2022_Davenport	130	ETrin_06/26
HD	2022_Davenport	130	ETrin_06/29
HD	2022_Davenport	130	ETrin_06/30
HD	2022_Davenport	130	ETrin_07/01
HD	2022_Davenport	130	ETrin_07/02
HD	2022_Davenport	130	ETrin_07/03
HD	2022_Davenport	130	ETrin_07/04
HD	2022_Davenport	130	ETrin_07/05
HD	2022_Davenport	130	ETrin_07/06
HD	2022_Davenport	130	ETrin_07/07
HD	2022_Davenport	130	ETrin_07/08
HD	2022_Prescott	129	Min.F_11/27
HD	2022_Prescott	129	Min.F_12/02
HD	2022_Prescott	129	Min.F_12/23
HD	2022_Prescott	129	Min.F_01/19
HD	2022_Prescott	129	Min.F_03/23
HD	2022_Prescott	129	Min.F_06/22
HD	2022_Prescott	129	Min.F_06/23
HD	2022_Prescott	129	Min.F_06/26
HD	2022_Prescott	129	Min.F_06/30
HD	2022_Prescott	129	Min.F_07/01
HD	2022_Prescott	129	Avg.F_11/27
HD	2022_Prescott	129	Avg.F_04/15
HD	2022_Prescott	129	Avg.F_05/16
HD	2022_Prescott	129	Avg.F_05/17
HD	2022_Prescott	129	Avg.F_06/19
HD	2022_Prescott	129	Avg.F_06/22
HD	2022_Prescott	129	Avg.F_06/23
HD	2022_Prescott	129	Avg.F_06/25
HD	2022_Prescott	129	Avg.F_06/26
HD	2022_Prescott	129	Avg.F_06/29
HD	2022_Prescott	129	Avg.F_07/03
HD	2022_Prescott	129	Avg.F_07/04
HD	2022_Prescott	129	Avg.F_07/06
HD	2022_Prescott	129	Max.F_04/06
HD	2022_Prescott	129	Max.F_04/15
HD	2022_Prescott	129	Max.F_04/16
HD	2022_Prescott	129	Max.F_04/17
HD	2022_Prescott	129	Max.F_04/30
HD	2022_Prescott	129	Max.F_05/16
HD	2022_Prescott	129	Max.F_06/03
HD	2022_Prescott	129	Max.F_06/19
HD	2022_Prescott	129	Max.F_06/20
HD	2022_Prescott	129	Max.F_06/22

HD	2022_Prescott	129	Max.F_06/24
HD	2022_Prescott	129	Max.F_06/25
HD	2022_Prescott	129	Max.F_06/29
HD	2022_Prescott	129	Max.F_07/03
HD	2022_Prescott	129	Max.F_07/06
HD	2022_Prescott	129	Max.F_07/07
HD	2022_Prescott	129	Avg1.5m.DP.F_10/24
HD	2022_Prescott	129	Avg1.5m.DP.F_01/12
HD	2022_Prescott	129	Avg1.5m.DP.F_05/27
HD	2022_Prescott	129	Avg1.5m.DP.F_06/06
HD	2022_Prescott	129	Avg1.5m.RH._11/16
HD	2022_Prescott	129	Avg1.5m.RH._11/26
HD	2022_Prescott	129	Avg1.5m.RH._02/27
HD	2022_Prescott	129	Avg1.5m.RH._04/10
HD	2022_Prescott	129	Avg1.5m.RH._04/19
HD	2022_Prescott	129	Avg1.5m.RH._04/21
HD	2022_Prescott	129	Avg1.5m.RH._05/15
HD	2022_Prescott	129	Avg1.5m.RH._05/21
HD	2022_Prescott	129	Avg1.5m.RH._05/27
HD	2022_Prescott	129	Avg1.5m.RH._06/02
HD	2022_Prescott	129	Avg1.5m.RH._06/06
HD	2022_Prescott	129	Avg1.5m.RH._06/08
HD	2022_Prescott	129	Avg1.5m.RH._06/21
HD	2022_Prescott	129	Avg1.5m.RH._06/22
HD	2022_Prescott	129	Avg1.5m.RH._06/25
HD	2022_Prescott	129	Avg1.5m.RH._07/02
HD	2022_Prescott	129	Avg1.5m.RH._07/03
HD	2022_Prescott	129	Avg1.5m.RH._07/04
HD	2022_Prescott	129	Avg1.5m.RH._07/05
HD	2022_Prescott	129	Avg1.5m.RH._07/06
HD	2022_Prescott	129	Avg1.5m.RH._07/07
HD	2022_Prescott	129	Avg1.5m.RH._07/08
HD	2022_Prescott	129	Min.F.1_06/23
HD	2022_Prescott	129	Min.F.1_06/24
HD	2022_Prescott	129	Min.F.1_06/25
HD	2022_Prescott	129	Min.F.1_06/26
HD	2022_Prescott	129	Min.F.1_06/27
HD	2022_Prescott	129	Min.F.1_06/30
HD	2022_Prescott	129	Avg.F.1_06/21
HD	2022_Prescott	129	Avg.F.1_06/22
HD	2022_Prescott	129	Avg.F.1_06/23
HD	2022_Prescott	129	Avg.F.1_06/24
HD	2022_Prescott	129	Avg.F.1_06/25
HD	2022_Prescott	129	Avg.F.1_06/26

HD	2022_Prescott	129	Avg.F.1_06/27
HD	2022_Prescott	129	TotPrecin_01/01
HD	2022_Prescott	129	TotPrecin_06/05
HD	2022_Prescott	129	TotalSolarRadMJ.m._11/08
HD	2022_Prescott	129	TotalSolarRadMJ.m._03/05
HD	2022_Prescott	129	EToin_04/12
HD	2022_Prescott	129	EToin_04/14
HD	2022_Prescott	129	EToin_04/15
HD	2022_Prescott	129	EToin_04/16
HD	2022_Prescott	129	EToin_04/18
HD	2022_Prescott	129	EToin_05/15
HD	2022_Prescott	129	EToin_05/16
HD	2022_Prescott	129	EToin_05/26
HD	2022_Prescott	129	EToin_06/20
HD	2022_Prescott	129	EToin_06/22
HD	2022_Prescott	129	EToin_06/23
HD	2022_Prescott	129	EToin_06/24
HD	2022_Prescott	129	EToin_06/25
HD	2022_Prescott	129	EToin_06/26
HD	2022_Prescott	129	EToin_06/29
HD	2022_Prescott	129	EToin_06/30
HD	2022_Prescott	129	EToin_07/02
HD	2022_Prescott	129	EToin_07/03
HD	2022_Prescott	129	EToin_07/04
HD	2022_Prescott	129	EToin_07/05
HD	2022_Prescott	129	EToin_07/06
HD	2022_Prescott	129	EToin_07/07
HD	2022_Prescott	129	EToin_07/08
HD	2022_Prescott	129	ETrin_04/14
HD	2022_Prescott	129	ETrin_04/15
HD	2022_Prescott	129	ETrin_04/16
HD	2022_Prescott	129	ETrin_04/18
HD	2022_Prescott	129	ETrin_05/15
HD	2022_Prescott	129	ETrin_05/16
HD	2022_Prescott	129	ETrin_05/26
HD	2022_Prescott	129	ETrin_05/27
HD	2022_Prescott	129	ETrin_06/21
HD	2022_Prescott	129	ETrin_06/22
HD	2022_Prescott	129	ETrin_06/23
HD	2022_Prescott	129	ETrin_06/24
HD	2022_Prescott	129	ETrin_06/25
HD	2022_Prescott	129	ETrin_06/26
HD	2022_Prescott	129	ETrin_06/29
HD	2022_Prescott	129	ETrin_06/30

HD	2022_Prescott	129	ETrin_07/01
HD	2022_Prescott	129	ETrin_07/02
HD	2022_Prescott	129	ETrin_07/03
HD	2022_Prescott	129	ETrin_07/04
HD	2022_Prescott	129	ETrin_07/05
HD	2022_Prescott	129	ETrin_07/06
HD	2022_Prescott	129	ETrin_07/07
HD	2022_Prescott	129	ETrin_07/08
HD	2022_Pullman	126	Min.F_12/02
HD	2022_Pullman	126	Min.F_01/19
HD	2022_Pullman	126	Min.F_03/23
HD	2022_Pullman	126	Min.F_06/22
HD	2022_Pullman	126	Min.F_06/23
HD	2022_Pullman	126	Min.F_06/26
HD	2022_Pullman	126	Min.F_06/27
HD	2022_Pullman	126	Min.F_06/29
HD	2022_Pullman	126	Min.F_07/03
HD	2022_Pullman	126	Min.F_07/04
HD	2022_Pullman	126	Min.F_07/07
HD	2022_Pullman	126	Avg.F_04/15
HD	2022_Pullman	126	Avg.F_05/16
HD	2022_Pullman	126	Avg.F_05/17
HD	2022_Pullman	126	Avg.F_06/19
HD	2022_Pullman	126	Avg.F_06/22
HD	2022_Pullman	126	Avg.F_06/23
HD	2022_Pullman	126	Avg.F_06/25
HD	2022_Pullman	126	Avg.F_06/26
HD	2022_Pullman	126	Avg.F_06/29
HD	2022_Pullman	126	Avg.F_07/03
HD	2022_Pullman	126	Avg.F_07/04
HD	2022_Pullman	126	Avg.F_07/06
HD	2022_Pullman	126	Avg.F_07/07
HD	2022_Pullman	126	Max.F_04/15
HD	2022_Pullman	126	Max.F_04/16
HD	2022_Pullman	126	Max.F_04/30
HD	2022_Pullman	126	Max.F_05/16
HD	2022_Pullman	126	Max.F_05/21
HD	2022_Pullman	126	Max.F_06/03
HD	2022_Pullman	126	Max.F_06/21
HD	2022_Pullman	126	Max.F_06/22
HD	2022_Pullman	126	Max.F_06/25
HD	2022_Pullman	126	Max.F_06/26
HD	2022_Pullman	126	Max.F_06/29
HD	2022_Pullman	126	Max.F_07/03

HD	2022_Pullman	126	Max.F_07/06
HD	2022_Pullman	126	Max.F_07/07
HD	2022_Pullman	126	Max.F_07/08
HD	2022_Pullman	126	Avg1.5m.DP.F_01/12
HD	2022_Pullman	126	Avg1.5m.DP.F_05/27
HD	2022_Pullman	126	Avg1.5m.DP.F_06/06
HD	2022_Pullman	126	Avg1.5m.RH._11/23
HD	2022_Pullman	126	Avg1.5m.RH._11/26
HD	2022_Pullman	126	Avg1.5m.RH._01/29
HD	2022_Pullman	126	Avg1.5m.RH._02/27
HD	2022_Pullman	126	Avg1.5m.RH._03/29
HD	2022_Pullman	126	Avg1.5m.RH._03/30
HD	2022_Pullman	126	Avg1.5m.RH._04/03
HD	2022_Pullman	126	Avg1.5m.RH._04/05
HD	2022_Pullman	126	Avg1.5m.RH._04/10
HD	2022_Pullman	126	Avg1.5m.RH._05/15
HD	2022_Pullman	126	Avg1.5m.RH._05/16
HD	2022_Pullman	126	Avg1.5m.RH._05/21
HD	2022_Pullman	126	Avg1.5m.RH._05/24
HD	2022_Pullman	126	Avg1.5m.RH._05/26
HD	2022_Pullman	126	Avg1.5m.RH._05/27
HD	2022_Pullman	126	Avg1.5m.RH._06/06
HD	2022_Pullman	126	Avg1.5m.RH._06/08
HD	2022_Pullman	126	Avg1.5m.RH._06/21
HD	2022_Pullman	126	Avg1.5m.RH._06/22
HD	2022_Pullman	126	Avg1.5m.RH._06/26
HD	2022_Pullman	126	Avg1.5m.RH._07/02
HD	2022_Pullman	126	Avg1.5m.RH._07/03
HD	2022_Pullman	126	Avg1.5m.RH._07/04
HD	2022_Pullman	126	Avg1.5m.RH._07/05
HD	2022_Pullman	126	Avg1.5m.RH._07/06
HD	2022_Pullman	126	Avg1.5m.RH._07/07
HD	2022_Pullman	126	TotPrecin_01/01
HD	2022_Pullman	126	TotPrecin_06/05
HD	2022_Pullman	126	TotalSolarRadMJ.m._11/08
HD	2022_Pullman	126	TotalSolarRadMJ.m._01/25
HD	2022_Pullman	126	TotalSolarRadMJ.m._03/05
HD	2022_Pullman	126	TotalSolarRadMJ.m._04/05
HD	2022_Pullman	126	TotalSolarRadMJ.m._05/26
HD	2022_Pullman	126	ETоin_12/12
HD	2022_Pullman	126	ETоin_02/27
HD	2022_Pullman	126	ETоin_04/06
HD	2022_Pullman	126	ETоin_04/14
HD	2022_Pullman	126	ETоin_04/16

HD	2022_Pullman	126	EToin_05/15
HD	2022_Pullman	126	EToin_05/16
HD	2022_Pullman	126	EToin_05/21
HD	2022_Pullman	126	EToin_05/26
HD	2022_Pullman	126	EToin_06/21
HD	2022_Pullman	126	EToin_06/22
HD	2022_Pullman	126	EToin_06/23
HD	2022_Pullman	126	EToin_06/24
HD	2022_Pullman	126	EToin_06/25
HD	2022_Pullman	126	EToin_06/26
HD	2022_Pullman	126	EToin_06/27
HD	2022_Pullman	126	EToin_06/29
HD	2022_Pullman	126	EToin_06/30
HD	2022_Pullman	126	EToin_07/02
HD	2022_Pullman	126	EToin_07/03
HD	2022_Pullman	126	EToin_07/04
HD	2022_Pullman	126	EToin_07/05
HD	2022_Pullman	126	EToin_07/06
HD	2022_Pullman	126	EToin_07/07
HD	2022_Pullman	126	ETrin_12/12
HD	2022_Pullman	126	ETrin_02/27
HD	2022_Pullman	126	ETrin_03/29
HD	2022_Pullman	126	ETrin_04/06
HD	2022_Pullman	126	ETrin_04/14
HD	2022_Pullman	126	ETrin_04/16
HD	2022_Pullman	126	ETrin_04/18
HD	2022_Pullman	126	ETrin_05/15
HD	2022_Pullman	126	ETrin_05/16
HD	2022_Pullman	126	ETrin_05/26
HD	2022_Pullman	126	ETrin_05/27
HD	2022_Pullman	126	ETrin_06/21
HD	2022_Pullman	126	ETrin_06/22
HD	2022_Pullman	126	ETrin_06/23
HD	2022_Pullman	126	ETrin_06/24
HD	2022_Pullman	126	ETrin_06/25
HD	2022_Pullman	126	ETrin_06/26
HD	2022_Pullman	126	ETrin_06/27
HD	2022_Pullman	126	ETrin_06/29
HD	2022_Pullman	126	ETrin_06/30
HD	2022_Pullman	126	ETrin_07/02
HD	2022_Pullman	126	ETrin_07/03
HD	2022_Pullman	126	ETrin_07/04
HD	2022_Pullman	126	ETrin_07/05
HD	2022_Pullman	126	ETrin_07/06

HD	2022_Pullman	126	ETrin_07/07
HD	2022_Pullman	126	ETrin_07/08
HD	2022_Ritzville	127	Min.F_11/27
HD	2022_Ritzville	127	Min.F_12/02
HD	2022_Ritzville	127	Min.F_12/23
HD	2022_Ritzville	127	Min.F_01/19
HD	2022_Ritzville	127	Min.F_03/23
HD	2022_Ritzville	127	Min.F_06/22
HD	2022_Ritzville	127	Min.F_06/23
HD	2022_Ritzville	127	Min.F_06/26
HD	2022_Ritzville	127	Min.F_06/30
HD	2022_Ritzville	127	Avg.F_04/15
HD	2022_Ritzville	127	Avg.F_05/16
HD	2022_Ritzville	127	Avg.F_05/17
HD	2022_Ritzville	127	Avg.F_06/19
HD	2022_Ritzville	127	Avg.F_06/22
HD	2022_Ritzville	127	Avg.F_06/23
HD	2022_Ritzville	127	Avg.F_06/25
HD	2022_Ritzville	127	Avg.F_06/26
HD	2022_Ritzville	127	Avg.F_06/29
HD	2022_Ritzville	127	Avg.F_07/03
HD	2022_Ritzville	127	Avg.F_07/04
HD	2022_Ritzville	127	Avg.F_07/06
HD	2022_Ritzville	127	Max.F_04/06
HD	2022_Ritzville	127	Max.F_04/15
HD	2022_Ritzville	127	Max.F_04/16
HD	2022_Ritzville	127	Max.F_04/30
HD	2022_Ritzville	127	Max.F_05/16
HD	2022_Ritzville	127	Max.F_06/03
HD	2022_Ritzville	127	Max.F_06/19
HD	2022_Ritzville	127	Max.F_06/20
HD	2022_Ritzville	127	Max.F_06/22
HD	2022_Ritzville	127	Max.F_06/24
HD	2022_Ritzville	127	Max.F_06/25
HD	2022_Ritzville	127	Max.F_06/26
HD	2022_Ritzville	127	Max.F_06/29
HD	2022_Ritzville	127	Max.F_07/03
HD	2022_Ritzville	127	Max.F_07/06
HD	2022_Ritzville	127	Max.F_07/07
HD	2022_Ritzville	127	Avg1.5m.DP.F_10/24
HD	2022_Ritzville	127	Avg1.5m.DP.F_01/12
HD	2022_Ritzville	127	Avg1.5m.DP.F_05/27
HD	2022_Ritzville	127	Avg1.5m.DP.F_06/06
HD	2022_Ritzville	127	Avg1.5m.RH._11/16

HD	2022_Ritzville	127	Avg1.5m.RH._11/26
HD	2022_Ritzville	127	Avg1.5m.RH._02/27
HD	2022_Ritzville	127	Avg1.5m.RH._04/10
HD	2022_Ritzville	127	Avg1.5m.RH._04/19
HD	2022_Ritzville	127	Avg1.5m.RH._04/21
HD	2022_Ritzville	127	Avg1.5m.RH._05/15
HD	2022_Ritzville	127	Avg1.5m.RH._05/21
HD	2022_Ritzville	127	Avg1.5m.RH._05/27
HD	2022_Ritzville	127	Avg1.5m.RH._06/02
HD	2022_Ritzville	127	Avg1.5m.RH._06/06
HD	2022_Ritzville	127	Avg1.5m.RH._06/08
HD	2022_Ritzville	127	Avg1.5m.RH._06/21
HD	2022_Ritzville	127	Avg1.5m.RH._06/22
HD	2022_Ritzville	127	Avg1.5m.RH._06/25
HD	2022_Ritzville	127	Avg1.5m.RH._07/02
HD	2022_Ritzville	127	Avg1.5m.RH._07/03
HD	2022_Ritzville	127	Avg1.5m.RH._07/04
HD	2022_Ritzville	127	Avg1.5m.RH._07/05
HD	2022_Ritzville	127	Avg1.5m.RH._07/06
HD	2022_Ritzville	127	Avg1.5m.RH._07/07
HD	2022_Ritzville	127	Avg1.5m.RH._07/08
HD	2022_Ritzville	127	Min.F.1_06/23
HD	2022_Ritzville	127	Min.F.1_06/24
HD	2022_Ritzville	127	Min.F.1_06/25
HD	2022_Ritzville	127	Min.F.1_06/26
HD	2022_Ritzville	127	Min.F.1_06/27
HD	2022_Ritzville	127	Min.F.1_06/30
HD	2022_Ritzville	127	Avg.F.1_06/21
HD	2022_Ritzville	127	Avg.F.1_06/22
HD	2022_Ritzville	127	Avg.F.1_06/23
HD	2022_Ritzville	127	Avg.F.1_06/24
HD	2022_Ritzville	127	Avg.F.1_06/25
HD	2022_Ritzville	127	Avg.F.1_06/26
HD	2022_Ritzville	127	Avg.F.1_06/27
HD	2022_Ritzville	127	TotPrecin_01/01
HD	2022_Ritzville	127	TotPrecin_06/05
HD	2022_Ritzville	127	TotalSolarRadMJ.m._11/08
HD	2022_Ritzville	127	TotalSolarRadMJ.m._03/05
HD	2022_Ritzville	127	EToin_04/12
HD	2022_Ritzville	127	EToin_04/14
HD	2022_Ritzville	127	EToin_04/15
HD	2022_Ritzville	127	EToin_04/16
HD	2022_Ritzville	127	EToin_04/18
HD	2022_Ritzville	127	EToin_05/15

HD	2022_Ritzville	127	EToin_05/16
HD	2022_Ritzville	127	EToin_05/26
HD	2022_Ritzville	127	EToin_06/20
HD	2022_Ritzville	127	EToin_06/22
HD	2022_Ritzville	127	EToin_06/23
HD	2022_Ritzville	127	EToin_06/24
HD	2022_Ritzville	127	EToin_06/25
HD	2022_Ritzville	127	EToin_06/26
HD	2022_Ritzville	127	EToin_06/29
HD	2022_Ritzville	127	EToin_06/30
HD	2022_Ritzville	127	EToin_07/02
HD	2022_Ritzville	127	EToin_07/03
HD	2022_Ritzville	127	EToin_07/04
HD	2022_Ritzville	127	EToin_07/05
HD	2022_Ritzville	127	EToin_07/06
HD	2022_Ritzville	127	EToin_07/07
HD	2022_Ritzville	127	EToin_07/08
HD	2022_Ritzville	127	ETrin_04/14
HD	2022_Ritzville	127	ETrin_04/15
HD	2022_Ritzville	127	ETrin_04/16
HD	2022_Ritzville	127	ETrin_04/18
HD	2022_Ritzville	127	ETrin_05/15
HD	2022_Ritzville	127	ETrin_05/16
HD	2022_Ritzville	127	ETrin_05/26
HD	2022_Ritzville	127	ETrin_05/27
HD	2022_Ritzville	127	ETrin_06/21
HD	2022_Ritzville	127	ETrin_06/22
HD	2022_Ritzville	127	ETrin_06/23
HD	2022_Ritzville	127	ETrin_06/24
HD	2022_Ritzville	127	ETrin_06/25
HD	2022_Ritzville	127	ETrin_06/26
HD	2022_Ritzville	127	ETrin_06/29
HD	2022_Ritzville	127	ETrin_06/30
HD	2022_Ritzville	127	ETrin_07/01
HD	2022_Ritzville	127	ETrin_07/02
HD	2022_Ritzville	127	ETrin_07/03
HD	2022_Ritzville	127	ETrin_07/04
HD	2022_Ritzville	127	ETrin_07/05
HD	2022_Ritzville	127	ETrin_07/06
HD	2022_Ritzville	127	ETrin_07/07
HD	2022_Ritzville	127	ETrin_07/08

Supplemental Figure 1

Figure S1. Locations from the Washington State University (WSU) winter wheat breeding program from which data on wheat lines were obtained and analyzed relating to datasets 1 to 5.

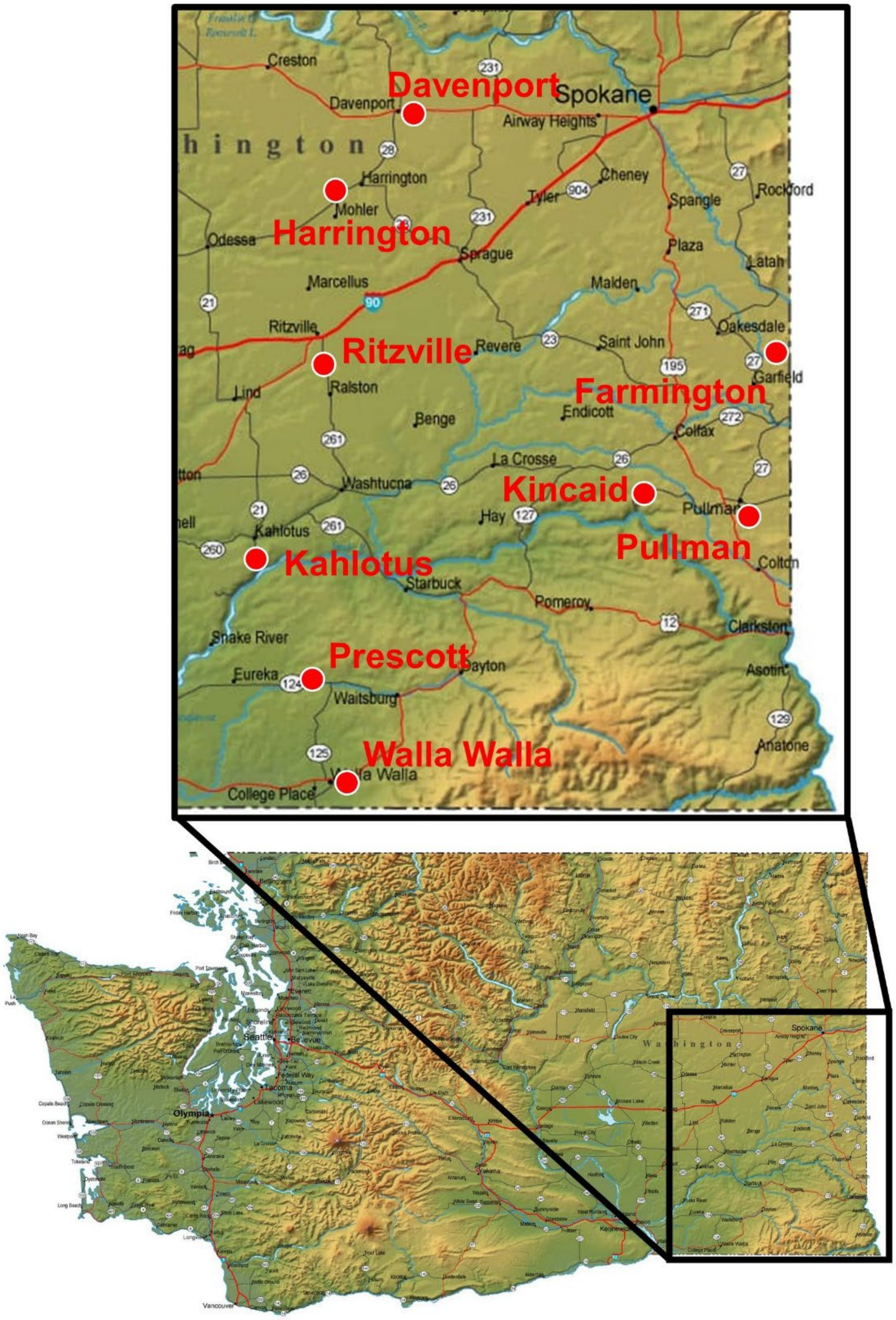

Supplement: Supplementary file 1 — Supplementary Material 1 [file 12864_2024_10438_MOESM1_ESM.pdf]
